# Human limits in next-generation fighter aviation: psychophysiological stressors, readiness, health, performance, and countermeasures

**DOI:** 10.3389/fphys.2026.1920799

**Published:** 2026-09-21

**Authors:** Jennifer F. Chan, Jing Zhang, Maria Y. Shiu, Fethi Bouak, Kaela Shea, Joan Saary, Oshin Vartanian, Shawn G. Rhind

**Affiliations:** 1 Defence Research and Development – Toronto Research Centre, Toronto, ON, Canada; 2 Canadian Forces Environmental Medicine Establishment, Toronto, ON, Canada; 3 Department of Medicine, Division of Occupational Medicine, University of Toronto, Toronto, ON, Canada; 4 Department of Psychology, University of Toronto, Toronto, ON, Canada; 5 Faculty of Kinesiology and Physical Education, University of Toronto, Toronto, ON, Canada

**Keywords:** aerospace medicine, autonomic regulation, biomarkers and biomonitoring, cognitive load, high-g exposure, hypobaria, military fighter pilots, unexplained physiological episodes

## Abstract

The transition to fifth- and sixth-generation fighter aircraft is increasing the psychophysiological demands placed on military aircrew. Next-generation fast-jet operations combine sustained and repetitive high-G loading; intermittent hypoxia, hyperoxia, and hypobaria associated with unexplained physiological episodes; prolonged single-seat missions; advanced helmet-mounted displays; acoustic and thermal stress; and high cognitive load from sensor fusion, automation, and manned-unmanned teaming. These exposures challenge autonomic regulation, cerebral and respiratory physiology, neurocognitive performance, sensory integration, and central nervous system resilience. This narrative review synthesizes evidence across cardiovascular-autonomic and hemodynamic function; respiratory physiology and unexplained physiological episodes in the on-board oxygen generating system era; cognitive workload, fatigue, and pilot experience; visual-oculomotor, vestibular, auditory, thermal, and musculoskeletal stress; biological markers of neural strain; and aircrew surveillance and countermeasures. The organizing construct is psychophysiological readiness: the integrated capacity to meet mission demands without disproportionate physiological cost or degraded decision quality. Evidence from direct fifth-generation studies, other fast-jet operations, simulators, and controlled aerospace analogues shows measurable changes in heart rate variability, cerebral oxygenation and perfusion, cortical activity, sensory function, and neurostructural biomarkers, but direct fifth-generation evidence remains limited and responses are strongly individualized and context dependent. Current surveillance remains largely episodic and domain-siloed. We therefore propose a staged monitoring framework combining validated cardiovascular-autonomic and hemodynamic measures with neurocognitive, sensory, wearable, and biomarker data, while treating AI/ML as an experimental analytic layer requiring prospective validation. Longitudinal, career-spanning monitoring could ultimately support earlier identification of vulnerability and evidence-based optimization of training, recovery, and countermeasures while preserving human aeromedical oversight.

## 1 Introduction

Military aviation has always existed at the intersection of human physiology and engineering ambition. From early descriptions of flying sickness and barometric physiology to modern combat aircraft, each technological generation has expanded both capability and human demand (Munroe, 1919; Marotte, 2006; Summerfield et al., 2018). Fifth-generation fighters combine low-observable design, high manoeuvrability, sensor fusion, networked information exchange, and helmet-mounted displays within an integrated combat system (Gertler, 2020; Gündüz, 2025; Svoboda et al., 2019; Richardson, 2019; Stensrud et al., 2020). The pilot therefore functions simultaneously as aircraft operator, sensor manager, tactical decision-maker, and physiological occupant of a flight envelope that routinely exceeds natural human tolerances (Clark, 2024; Elliott and Schmitt, 2019; Şengöz, 2026).

The F-35, operational with the U.S. Marine Corps since 2015 (U.S. Marine Corps, 2015), illustrates the global transition to fifth-generation systems and the accompanying aeromedical challenges spanning vision, audition, cognition, physical strain, life support, and autonomy (Gündüz, 2025; Keijsper, 2024). Sixth-generation concepts are projected to extend this trajectory through deeper AI integration, crewed-uncrewed teaming, and operation across broader altitude and mission envelopes; claims concerning these systems remain necessarily prospective because they are not yet operational in mature form (Hagos et al., 2024; Şengöz, 2026; Chattopadhyay, 2025; Elhefnawy, 2021). Some forward-looking aerospace concepts may also extend operations toward near-space altitudes (conventionally ~ 20–100 km above sea level, above most conventional aviation but below the lowest stable Earth orbits) although such operating environments remain prospective rather than established features of sixth-generation fighter operations. The defining issue is therefore convergence: each capability that expands aircraft reach also expands the physiological, cognitive, and operational envelope in which the pilot must perform (Coffey et al., 2026; Martins, 2016).

The central challenge addressed in this review is that aerospace physiology and health surveillance have not kept pace with this escalation. The Unexplained Physiological Episode (UPE) experience illustrates how life-support design assumptions can be outpaced by the interacting respiratory demands of high-G, altitude, pressure breathing, and workload, while the lack of cockpit-compatible physiological sensing can leave investigators without contemporaneous data on pilot state (Elliott and Schmitt, 2019; Jia et al., 2026; Lan et al., 2023; Lengersdorf et al., 2025).

Cognitive workload presents a parallel dilemma. Automation and sensor fusion can reduce manual tasks yet create supervisory, trust-calibration, and edge-case demands, with potential consequences for situational awareness and skill retention (Şengöz, 2026; Uhuegho et al., 2026; Gil et al., 2012). As crewed-uncrewed teaming matures, the pilot may increasingly command autonomous elements as well as fly the aircraft, making physiological and cognitive state part of the performance of a wider combat system (Korentsides et al., 2024; Yıldız, 2024). These demands interact in ways that siloed aerospace-medicine models may not adequately capture (Clark, 2024; Weigand and Embacher, 2024).

This review integrates simulator, in-flight, operational, and mechanistic evidence on next-generation fighter psychophysiology. We use the autonomic nervous system (ANS) as one important integrative lens while examining respiratory, cognitive, sensory, musculoskeletal, biomarker, sleep, and psychosocial domains in parallel (Bustamante-Sanchez et al., 2020; Fuentes-Garcia et al., 2021; Zhang et al., 2020). The organizing concept is psychophysiological readiness (Calado et al., 2025; Miller, 2025; Villafaina et al., 2021), developed below as a longitudinal, multidomain framework rather than a single physiological score.

Because psychophysiological readiness organizes the review that follows, we define it explicitly rather than leaving it as an evocative phrase. We use the term to denote the moment-to-moment and cumulative capacity of a pilot’s physiological and cognitive systems to meet the demands of a given mission phase without incurring disproportionate physiological cost or degraded decision quality. As we use it, readiness is deliberately broader than, but overlapping with, several related and better-operationalized constructs: workload describes the immediate demand placed on attentional and executive resources by a task, and is usually treated as an input to readiness rather than a synonym for it; resilience describes the capacity to recover function after a stressor and is one component of readiness rather than its entirety; fitness denotes baseline physical and physiological capacity, largely independent of the task at hand; fatigue describes a specific, time-dependent depletion of attentional or physical capacity; and allostatic load, introduced below, describes cumulative physiological wear from repeated stress-response activation across a career, of which readiness is best understood as the present-moment expression. We treat readiness as a multi-domain composite rather than a single latent variable: it spans autonomic and cardiovascular regulation, respiratory and oxygenation status, neurocognitive function, sensory and vestibular integration, sleep and circadian state, and psychological state, and no single measure reviewed in this paper, including HRV, is proposed as a sufficient index of it on its own.

This definition carries direct measurement implications, which we return to in Section 10. Quantifying readiness requires: (i) individualized baselines for each relevant domain, since a value representing significant deviation for one pilot may be unremarkable for another; (ii) repeated, ideally continuous or near-continuous, measurement capable of detecting within-person change over the course of a mission, a training cycle, or a career, rather than isolated snapshots compared only to population norms; (iii) a means of distinguishing adaptive activation, in which a physiological response is proportionate to demand and resolves with recovery, from maladaptive strain, in which the response is disproportionate, fails to resolve, or predicts subsequent performance or health decrements; and (iv) validation against independent outcomes, such as objectively measured performance, error rates, or downstream health events, before any composite readiness index is used to inform training, scheduling, or fitness-to-fly decisions. No existing measure or combination of measures reviewed here has been validated to this standard in fifth- or sixth-generation fighter populations; we treat psychophysiological readiness as a conceptual and organizing framework for this literature, not as a validated clinical or operational instrument, and we flag this explicitly in the Limitations of This Review section below.

### 1.1 Literature search and synthesis approach

This narrative review synthesizes open-literature and unclassified evidence from aerospace medicine, human factors, physiology, psychology, neuroscience, occupational health, and military aviation relevant to fifth- and emerging sixth-generation fighter operations. No classified, controlled, export-restricted, operationally sensitive, or proprietary technical information was used. Peer-reviewed articles, technical reports, aeromedical guidance, government and defence research publications, and authoritative reviews were identified through targeted searches of PubMed, Scopus, Web of Science, and Google Scholar, supplemented by reference-list screening, forward and backward citation chaining, and targeted retrieval of publicly available aerospace physiology and military aviation reports. Searches were conducted iteratively by the author team between 2025 and 18 August 2026, including a final currency check immediately before resubmission.

Search terms were used singly and in Boolean combinations and adapted to each database. They encompassed fighter and high-performance aviation; fifth- and sixth-generation aircraft; psychophysiological readiness; autonomic, cardiovascular, and cerebrovascular function; heart rate variability (HRV); electrocardiography, photoplethysmography, impedance cardiography, near-infrared spectroscopy (NIRS/fNIRS), electroencephalography (EEG), and electrodermal activity; wearable sensing and smart textiles; cognitive and mental workload; fatigue, sleep loss, and circadian disruption; high-G/+Gz acceleration and G-induced loss of consciousness (G-LOC); hypoxia, hyperoxia, hypobaria, decompression stress, on-board oxygen generating systems (OBOGS), and unexplained physiological episodes (UPEs); helmet-mounted displays, sensor fusion, automation, and human–machine teaming; visual, oculomotor, vestibular, auditory, thermal, and musculoskeletal stress; biomarkers, blood–brain barrier and neurovascular injury, white matter hyperintensities, and neuroimaging; and allostasis, resilience, pilot performance, aircrew health surveillance, training, and countermeasures.

Eligibility and synthesis followed principles consistent with SANRA guidance for narrative reviews, rather than a prospectively registered PRISMA-style systematic-review protocol. A narrative approach was selected because of the breadth of the topic and the substantial heterogeneity in study populations, aircraft platforms, exposure paradigms, methodologies, and outcomes. Sources were included when they reported primary data, validated methods, or authoritative synthesis relevant to psychophysiological stressors, monitoring, or countermeasures in military or civil aviation, spaceflight, or closely analogous high-performance/high-hazard settings. Sources were excluded when they lacked meaningful aircrew relevance, involved non-human models without translational relevance, or could not be verified against the original source. No language or study-design restriction was imposed *a priori*, although non-English studies were included only when sufficient English-language information was available for verification.

Evidence selection was conducted collaboratively by authors with expertise in aerospace physiology, occupational medicine, psychology, and biomedical engineering, with thematic responsibility and cross-checking across overlapping domains. Formal independent dual screening and a standardized risk-of-bias instrument were not used; instead, evidence was qualitatively weighted according to study design, sample size, peer-review status, methodological quality, and directness of relevance to next-generation fighter operations. This weighting is reflected in the language used throughout the review, including terms such as preliminary, small-sample, case-based, and hypothesis-generating. Because searches were iterative and not prospectively archived as a systematic-review protocol, exact historical record counts, duplicate-removal totals, and database-specific search strings cannot be reconstructed reliably; therefore, a PRISMA flow diagram is not presented. These methodological constraints are acknowledged further in Section 13.

A central challenge is that direct empirical evidence from fifth-generation fighter operations remains comparatively limited, while operational sixth-generation data do not yet exist. Accordingly, the review distinguishes, where possible, among five levels of evidence: direct findings from fifth-generation platforms and pilots; evidence from other military fast-jet or rotary-wing aircrew; controlled aerospace analogues such as centrifuge, altitude-chamber, and high-fidelity simulator studies; mechanistic extrapolation from relevant occupational, clinical, laboratory, or spaceflight populations; and forward-looking inference regarding anticipated sixth-generation systems. Claims in the latter category are explicitly framed as projections rather than demonstrated effects. Where a key conclusion rests primarily on a single study, the study population, platform, or sample size is generally identified in-text to allow readers to judge its evidentiary weight.

Overall, priority was given to evidence involving military fast-jet pilots, operational aircrew, centrifuge and hypoxia/hypobaria paradigms, high-fidelity simulators, and operational flight studies. Findings were synthesized narratively, with emphasis on convergence across autonomic, cardiovascular, neurocognitive, sensory, psychosocial, musculoskeletal, and biomarker domains, and on their relevance to pilot performance, health surveillance, training, and countermeasure development. Where direct next-generation evidence was unavailable, mechanistically relevant findings from legacy aviation and validated aerospace analogues were incorporated cautiously and identified as such. Representative empirical studies and the qualitative evidence maturity of candidate monitoring technologies are summarized in **Tables 1** and **2**.

**Table 1:** Representative empirical studies across major psychophysiological domains relevant to fighter aviation.

Study/population	Platform/exposure	Measures and principal finding	Principal limitation	Evidence tier*
Raff et al. (2025); n=20 experienced F-35 pilots	Operational F-35 flight-training exercise	Serial salivary cortisol and self-report; 35% showed no measurable flight-related cortisol rise, and pre-flight cortisol was inversely related to cumulative flight hours, consistent with experience-related HPA habituation.	Small, selected cohort; exploratory endocrine endpoint; diurnal and contextual influences limit individual interpretation.	I - direct fifth-generation
Jones et al. (2026); n=403 UK Armed Forces pilots	Operational military aviation; retrospective survey of +Gz-related altered consciousness	21.6% reported at least one G-related altered-consciousness event; recency of +Gz exposure was identified as an important modifiable factor.	Self-report and recall bias; heterogeneous platforms/exposure histories; does not establish prospective risk thresholds.	II - other operational aircrew
Nesci et al. (2026); n=20 Eurofighter Typhoon pilots	Real high-performance flight, up to +9Gz	Post-flight brachial flow-mediated dilation decreased with transient baseline vasodilation, supporting an acute vascular/endothelial response to high-performance flight.	Small sample; acute post-flight endpoint; uncertain persistence and clinical significance.	II - other fast-jet aircrew
Dussault et al. (2009); n=40 military pilots	Operational missions in Afghanistan	Post-flight autonomic recovery differed by mission profile; longer flights showed parasympathetic rebound whereas shorter, higher-intensity missions showed greater sympathetic predominance at landing.	Mission heterogeneity and post-flight sampling; HRV is non-specific and context dependent.	II - operational military aircrew
Kurihara et al. (2007); n=141 pilots	Human centrifuge/+Gz exposure to G-LOC	NIRS showed an ~ 15% fall in frontal cortical tissue oxygenation index at G-LOC, supporting cerebral oxygenation as a mechanistic marker of acceleration stress.	Controlled analogue rather than operational flight; endpoint is G-LOC physiology, not routine readiness classification.	III - controlled aerospace analogue
Reuten et al. (2026); n=14 experienced male F-16 pilots	Operationally relevant F-16 simulator mission	Subjective mental effort increased across mission segments, but EEG, heart-rate, and electrodermal measures did not systematically distinguish workload and explained only 2.2% of subjective workload variance.	Small male sample; simulator setting; limited task-separation may have reduced physiological discrimination.	III - high-fidelity simulator
Svensson et al. (1988); n=21 Swedish military attack pilots	Military flight missions	Urinary adrenaline varied with mission condition and experience, illustrating training/experience as a moderator of neuroendocrine stress response.	Older platform era; urinary biomarker has limited temporal resolution and uncertain transfer to next-generation systems.	II - other fast-jet aircrew

*Evidence tiers correspond to the hierarchy defined in Section 1.1: I, direct fifth-/sixth-generation evidence; II, other operational fast-jet or military aircrew; III, controlled aerospace analogue. This table is representative rather than exhaustive and is intended to make evidence provenance and limitations more visible; it is not a formal risk-of-bias assessment.

**Table 2:** Qualitative evidence maturity and operational readiness of candidate monitoring technologies for next-generation fighter aviation.

Technology/modality	Representative outputs	Evidence status	Key operational limitations	Near-term role
ECG/HRV	Heart rate, rhythm, HRV, recovery kinetics	Established measurement; moderate aerospace-operational evidence	Motion, posture, respiration, pressure breathing, context dependence; no universal readiness thresholds	Baseline and recovery profiling; adjunct workload/autonomic monitoring
Pulse oximetry/respiratory monitoring	SpO_2_, respiratory rate, oxygenation trends	Established sensors; moderate operational evidence	Motion and low-perfusion artefact; mask/pressure-system interactions; delayed or non-specific changes	Hypoxia/UPE contextual monitoring with respiratory and aircraft-system data
Electrical bioimpedance/ICG	Stroke volume, cardiac output, systolic time intervals, thoracic-fluid/hemodynamic trends	Mature non-invasive method; limited fighter-specific in-flight validation	Motion and respiration artefact, electrode placement, thoracic geometry, algorithm dependence; absolute accuracy varies	Within-subject hemodynamic trend monitoring alongside ECG/HRV
EEG/fNIRS/EDA/eye tracking	Cortical activity, cerebral oxygenation, arousal, gaze and oculomotor metrics	Strong experimental evidence; low-to-moderate operational readiness	Sensor burden, artefact, placement stability, calibration, environmental interference	Research-grade workload, fatigue, and sensory-state characterization
Neuroimaging/fluid biomarkers	WMH, neural injury, neurovascular, inflammatory and endocrine markers	Established laboratory/imaging methods; not continuous operational monitors	Sampling timing, specificity, cost, logistics, uncertain individual thresholds	Periodic longitudinal surveillance and mechanistic studies
Smart textiles/multimodal wearables	Integrated cardiac, respiratory, thermal, motion and biochemical signals	Emerging; heterogeneous validation	Durability, power, calibration drift, data loss, comfort, interoperability	Field research and staged validation in simulator/centrifuge/live flight
AI/ML multimodal fusion and adaptive decision support	State classification, trajectory prediction, composite readiness estimates	Promising experimental evidence; low readiness for safety-critical use	Small datasets, domain shift, external validation, explainability, false alarms, governance	Offline analytics and supervised decision support pending prospective validation

Categories reflect the evidence synthesized in this review and are not formal technology-readiness-level assignments.

## 2 Operational stressors in fifth- and sixth-generation fighter aviation

Next-generation fighter operations expose pilots to a convergent, interacting set of stressors (see **Figure 1**) that cannot be understood in isolation (Causse et al., 2024; Lejeune, 2026; Masi et al., 2023). Sustained, repetitive +Gz loading imposes cardiovascular, respiratory, musculoskeletal, and cognitive demands that extend beyond traditional acceleration tolerance (Burton et al., 1977; Gillingham, 1988; Shang et al., 2025; Summerfield et al., 2018). Emerging evidence suggests intermittent hypoxia, hyperoxia, and hypobaria in modern fast-jet aviation may present less as classical altitude sickness and more as UPEs, arising from non-linear interactions among OBOGS delivery, altered respiratory mechanics, tight-fitting flight equipment, workload, and +Gz exposure: oxygen-delivery dynamics influence ventilatory drive, anti-G straining increases breathing resistance, and workload or +Gz exposure raises oxygen demand while reducing physiological reserve (Konings et al., 2025; Powell and Garcia, 2000; Sheel, 2023).

**Figure 1:**
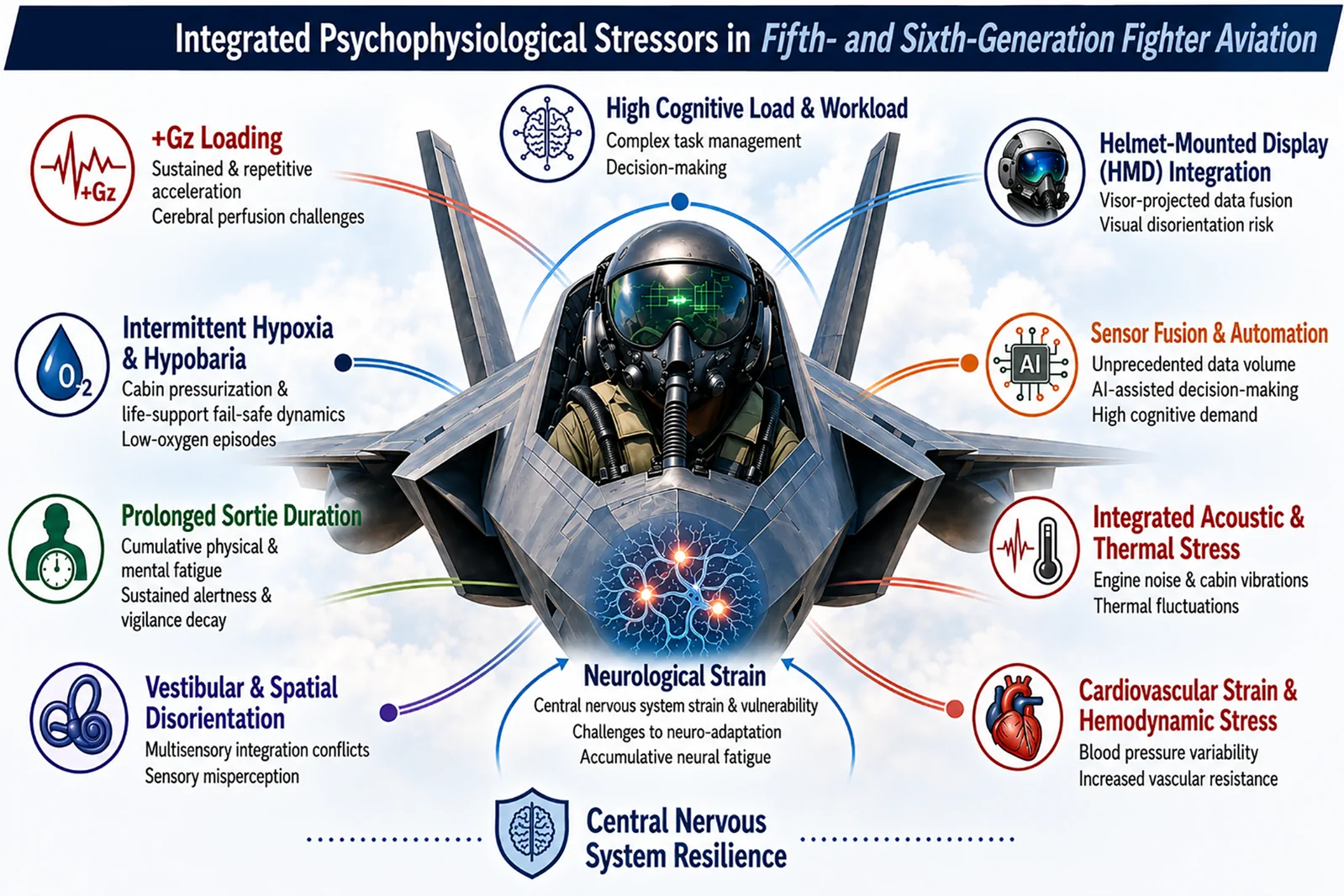
Integrated psychophysiological stressors in fighter aviation. Next-generation fighter operations, exemplified by platforms such as the F-35, expose pilots to interacting physiological, cognitive, sensory, and environmental stressors that converge on autonomic regulation, multisensory integration, and CNS resilience. Sustained +Gz loading challenges cerebral perfusion and cardiovascular control, while intermittent hypoxia and hypobaria create vulnerabilities in pressurization and life-support systems. Prolonged sorties, helmet-mounted displays, sensor fusion, automation, acoustic and thermal stress, and vestibular–spatial disorientation further increase allostatic load and physiological cost, often despite apparently preserved performance. This framework underscores the need for integrated, longitudinal monitoring of pilot health and performance in next-generation air combat.

Prolonged, high-altitude sorties enabled by modern aircraft endurance and aerial refuelling can produce cumulative fatigue that selectively impairs vigilance and sustained attention even when executive function appears preserved (Albers et al., 2025; Rosa et al., 2022), a dissociation that may increase operational risk by leaving pilots capable of complex task execution while fully engaged but more vulnerable to missed cues and delayed threat detection during extended or monotonous mission phases. Advanced HUD and sensor-fusion systems enhance situational awareness but also impose visual, vestibular, and cognitive demands that introduce new risk patterns (Compierchio et al., 2023; Morfitt, 2022; Morgan-Warren and Woodcock, 2014). Intense cockpit acoustic environments contribute to chronic hearing risk, auditory stress, and elevated listening workload, particularly when pilots must maintain speech intelligibility under high tempo and competing sensory demands (Danielson et al., 2019; Fan et al., 2022; Muhr et al., 2019; Rood and James, 2024; Shukla et al., 2025). Finally, the cognitive complexity associated with automation, teaming, and multi-domain information management challenges the pilot’s neurocognitive architecture in ways existing training, selection, and surveillance paradigms were not designed to address (Enriquez-Geppert et al., 2024; Schmidt, 2026).

Critically, these stressors interact and compound rather than operate independently (Lejeune, 2026; Tait et al., 2024): G-force amplifies hypoxia risk, and cognitive overload impairs the pilot’s capacity to recognize and respond to physiological symptoms, forming a positive feedback loop between stressor and stress response (Villafaina et al., 2021). Acute and recurrent stressors may accumulate across training and operational timelines, contributing to individualized patterns of neurological, cardiovascular, musculoskeletal, and psychological strain difficult to detect using conventional episodic aeromedical assessment alone (Summerfield et al., 2018; Tornero-Aguilera et al., 2025; Яковенко et al., 2025). **Table 3** summarises the principal stressor domains, their measurable signatures, and current and emerging monitoring and mitigation strategies.

**Table 3:** Psychophysiological stressors in next-generation fighter aviation: domains, measures, mitigation strategies.

Operational stressor	Primary domains affected	Representative measures	Operational relevance	Potential mitigation/monitoring
Sustained & repetitive +Gz	Autonomic, cardiovascular, neuromuscular, cognitive	HRV(time/frequency/nonlinear); BP surrogates; ICG-derived stroke volume/cardiac output; muscle fatigue; NIRS cerebral oxygenation	Reduced cerebral perfusion margin; G-LOC and A-LOC risk; degraded tolerance under cumulative load	Individualized G-tolerance profiling; ANS recovery monitoring; adaptive sortie scheduling; Auto-GCAS
Intermittent hypoxia/hypobaria & UPEs	Autonomic, cognitive, neural, respiratory	HRV suppression; cerebral oxygenation; SpO2; reaction time; psychomotor vigilance; eye tracking	Impaired insight into self-impairment; decision latency; acceleration atelectasis; UPE risk	Integrated hypoxia + workload monitoring; OBOGS redesign; real-time alerting; normobaric training
Prolonged sortie duration	Cognitive, autonomic, affective, vigilance	Vigilance metrics; HRV drift; fatigue indices (SPFI); emotional valence; PVT response time	Hidden fatigue; 7-h vigilance cliff; late-mission error risk; preserved executive but degraded sustained attention	Longitudinal fatigue profiling; adaptive task reallocation; exposure-recovery optimization
Sensor fusion & automation	Cognitive, executive control, trust calibration	Workload indices (NASA-TLX); task-switch cost; EEG/fNIRS proxies; eye fixation entropy	Automation surprise; trust miscalibration; complacency; skill degradation in degraded-mode operations	Adaptive automation; cognitive state-aware decision support; manual skill maintenance protocols
Helmet-mounted displays (HMDs)	Visual, oculomotor, cognitive, musculoskeletal	Eye-tracking; blink rate; saccadic velocity; fixation entropy; pupil diameter; NVG-associated depth perception	Visual fatigue; attentional tunneling; spatial disorientation; neck loading from HMD mass	Display ergonomics; visual load thresholds; HMD mass optimization; oculomotor fatigue screening
Vestibular conflict & spatial disorientation	Vestibular, autonomic, cognitive, spatial orientation	Postural instability; VOR gain; cortical arousal; autonomic activation during SD exposure	20-38% of fatal military accidents; SD-induced control reversal; HMD-specific illusions	Vestibular adaptation profiling; simulator-based SD training; conformal augmented reality displays
Acoustic load & communication demands	Auditory, cognitive, cardiovascular	Speech-in-noise performance; auditory fatigue markers; EEG alpha/beta/theta; ECG; EDA under noise	Delayed response to audio alerts; SA degradation; cumulative hearing loss; cognitive-auditory dual burden	Hearing-aware communication systems; active noise cancellation; 3D audio alerting; auditory workload monitoring
Musculoskeletal loading (cervical/lumbar)	Neuromuscular, structural, pain, performance	Cervical ROM; EMG; cervical disc height; FEM modelling; pain prevalence surveys	High prevalence of neck/back symptoms; potential effects on head mobility, fatigue, discomfort, and flight safety	Neck/trunk conditioning; HMD mass and mass-distribution optimization; ergonomic seat/restraint design; sex- and anthropometry-inclusive equipment standards
Cumulative operational exposure	Multi-system: all above domains	Longitudinal trends in HRV, hemodynamic indices (including ICG), WMH, cognitive performance, biomarkers, musculoskeletal status	Subclinical risk accumulation; career-span neurological burden; WMH associated with cognitive decline	Career-span psychophysiological monitoring; individualized baselines; early intervention triggers; AI-ML analytics
Thermal load	Cardiovascular, autonomic, cognitive, hydration	Cockpit/skin temperature; core-temperature proxy; HR/HRV; hydration status; perceived thermal strain	Additional cardiovascular and attentional burden; confounds physiological monitoring; may interact with hypoxia/workload	Environmental control; hydration; heat acclimation; cooling strategies; context-aware physiological interpretation

+Gz, positive acceleration; A-LOC, almost loss of consciousness; AI-ML, artificial intelligence–machine learning; ANS, autonomic nervous system; Auto-GCAS, automatic ground collision avoidance system; BP, blood pressure; ECG, electrocardiography; EDA, electrodermal activity; EEG, electroencephalography; EMG, electromyography; FEM, finite element modelling; fNIRS, functional near-infrared spectroscopy; G-LOC, G-induced loss of consciousness; HMD, helmet-mounted display; HRV, heart rate variability; NASA-TLX, National Aeronautics and Space Administration Task Load Index; NIRS, near-infrared spectroscopy; NVG, night vision goggles; OBOGS, on-board oxygen generating system; PVT, psychomotor vigilance task; ROM, range of motion; SA, situational awareness; SD, spatial disorientation; SPFI, Samn–Perelli Fatigue Index; SpO_2_, peripheral oxygen saturation; UPE, unexplained physiological episode; VOR, vestibulo-ocular reflex; WMH, white matter hyperintensity.

### 2.1 Thermal and musculoskeletal stress as cross-cutting loads

Thermal strain is a cross-cutting rather than merely comfort-related exposure. Cockpit heat, protective clothing, dehydration, and metabolic heat production can increase cardiovascular demand and fatigue while also altering HR, HRV, skin temperature, and other signals proposed for workload or readiness monitoring. Combined-stressor research has specifically examined heat load together with hypobaric hypoxia, underscoring that thermal effects should be interpreted in the context of concurrent oxygenation and cognitive demands rather than as an isolated environmental variable (Bottenheft et al., 2023). In next-generation aircraft, the practical implication is twofold: thermal state should be measured as an exposure and confounder when interpreting wearable physiology, and environmental-control, hydration, acclimation, and cooling strategies should be evaluated as performance countermeasures under mission-relevant combined exposures.

Musculoskeletal strain is similarly embedded within the wider psychophysiological system. Repeated +Gz loading, restraint geometry, prolonged static postures, and the mass and inertia of helmet/HMDS assemblies increase cervical and lumbar loading, particularly during tactically necessary head and trunk repositioning. Musculoskeletal symptoms may in turn degrade head mobility, visual acquisition, concentration, sleep, and tolerance of subsequent sorties, linking local tissue burden to cognitive and operational performance. Preliminary military-aviation biomarker work further suggests that inflammatory markers such as MCP-1 covary with neck and back pain frequency, although such findings remain associative rather than diagnostic (Bron et al., 2024). Countermeasures should therefore combine conditioning and movement-strategy training with engineering controls such as helmet mass-distribution optimization, seat/restraint ergonomics, and equipment fit across sex and anthropometric ranges; however, comparative trials defining optimal protocols for fifth-generation aircrew remain limited.

## 3 Autonomic–cardiovascular responses: HRV & the ANS as central integrators

The ANS innervates and regulates multiple organ systems through the dynamic balance between sympathetic (SNS) and parasympathetic (PNS) activity (Wehrwein et al., 2016). Psychological and physiological stressors trigger SNS activation and PNS withdrawal, and the proportionate balance of the two allows effective, adaptive responses to stress (Johnson, 2024; Oubaid, 2023). In fighter aviation, the ANS occupies a unique position as both a target of concurrent operational stressors and a sensitive readout of their cumulative impact on the pilot’s physiological state (Taha et al., 2025).

Allostasis and allostatic load provide a powerful framework for understanding cumulative physiological burden in high-performance aviation (**Figure 2**) (McEwen, 2006; Pfaltz and Schnyder, 2023; Summerfield et al., 2018). Allostasis refers to adaptive physiological stability under stress through HPA and SAM activation; when repeatedly activated without adequate recovery, allostatic load accumulates as multi-system wear and tear (Agorastos and Chrousos, 2022; Goldstein and McEwen, 2002; McEwen, 1998). The stressor profile of next-generation fighter operations maps closely onto this construct: repeated G-force activates cardiovascular and neuroendocrine pathways, intermittent hypoxia promotes oxidative and inflammatory burden, sleep restriction produces cumulative neurobiological strain, and organisational pressures add psychosocial load across the career span (Corrigan et al., 2021; Masi et al., 2023; McEwen and Karatsoreos, 2015). In 406 pilots, higher psychosocial stress was associated with greater sleep disturbance, fatigue, depression, and anxiety, with 44.7% reporting impaired wellbeing, 18.7% screening positive for depression, and 76% reporting severe or high fatigue despite being scheduled for only 60% of legally permitted duty hours (Venus, 2022), demonstrating that aviation-related allostatic burden translates into measurable population-level morbidity (Feigel et al., 2025b; Karatsoreos and McEwen, 2011; McEwen, 2017).

**Figure 2:**
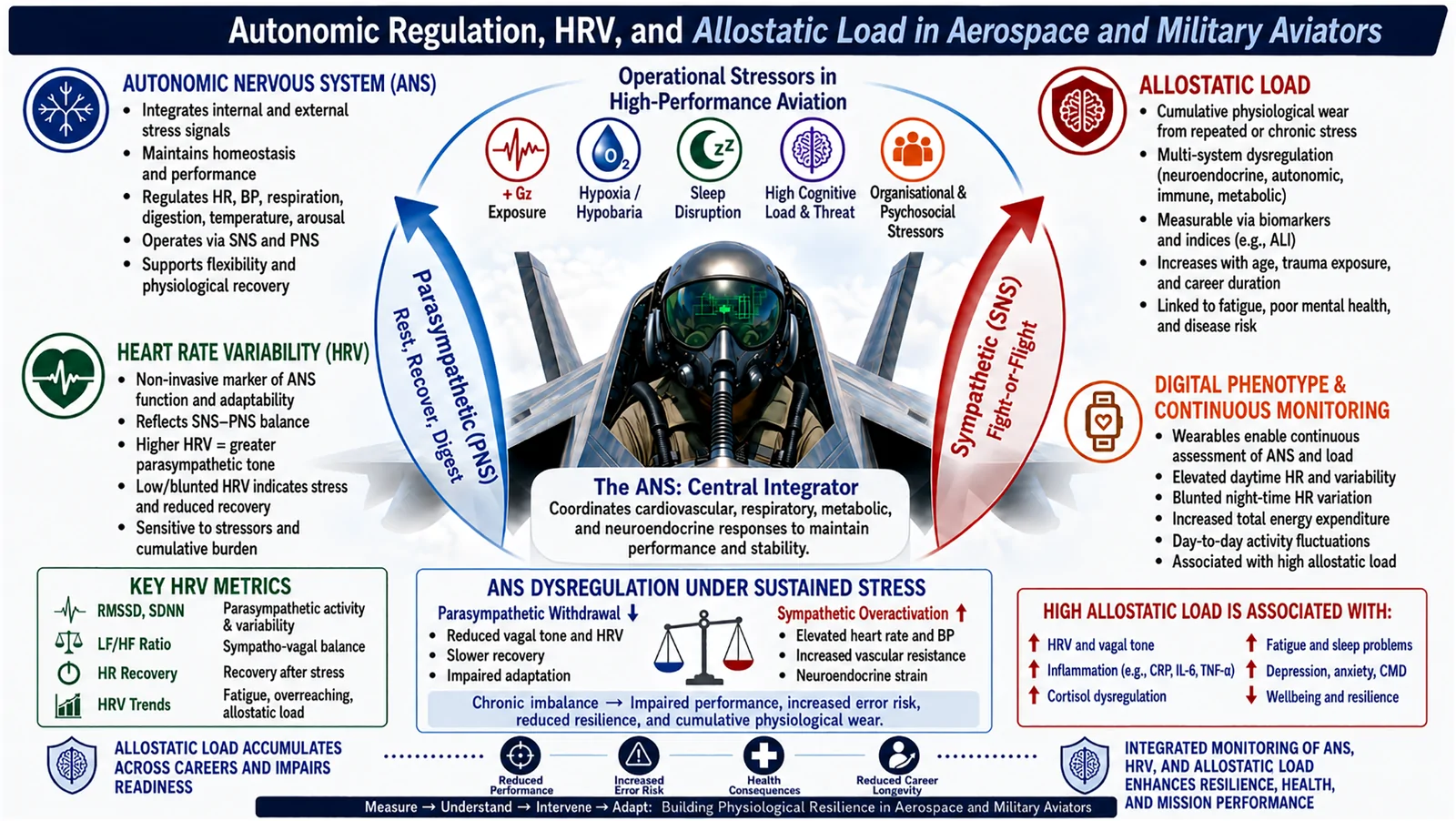
Autonomic regulation, heart rate variability, and allostatic load in military aviators. This figure presents a conceptual framework in which the autonomic nervous system (ANS) integrates internal and external demands during high-performance aviation. Acute sympathetic activation supports cardiovascular mobilization, arousal, and task engagement during stressors such as +Gz exposure, hypoxia/hypobaria, cognitive and threat load, sleep and circadian disruption, and organizational or psychosocial demands. In contrast, parasympathetic activity supports vagal regulation, recovery, and physiological adaptation. Heart rate variability (HRV) provides a non-invasive marker of ANS regulation and physiological flexibility, with reduced HRV, blunted HRV reactivity, or delayed heart-rate recovery potentially indicating acute strain, incomplete recovery, fatigue, or reduced resilience. With repeated exposure and insufficient recovery, imbalance between sympathetic activation and parasympathetic restoration may contribute to cumulative multisystem wear, conceptualized as allostatic load. The framework highlights the potential value of wearable, cockpit-embedded, and integrated monitoring approaches for tracking ANS and HRV dynamics, identifying maladaptive stress–recovery patterns earlier, and supporting individualized strategies for performance optimization, recovery, health protection, and long-term aviator readiness. RMSSD, root mean square of successive differences; SDNN, standard deviation of normal-to-normal intervals; LF/HF, low-frequency/high-frequency ratio; BP, blood pressure; HR, heart rate; ALI, allostatic load index. LF/HF is shown as a commonly reported HRV metric and should not be interpreted as a direct measure of sympathovagal balance.

The allostatic load index (ALI) integrates neuroendocrine, autonomic, and immune biomarkers into a composite measure of cumulative burden (Juster et al., 2010; Mauss et al., 2015; McCrory et al., 2023; Seeman et al., 2001). ALI increased significantly over a 10-week USMC Officer Candidates School course, with 88.2% of men classified as having high allostatic load by course completion (Feigel et al., 2025c), and high ALI is associated with a wearable-detectable digital phenotype characterized by elevated daytime heart rate and blunted sleep-related heart-rate variation, with each standard-deviation increase in these metrics associated with a 5-93% increase in the odds of high allostatic load (Feigel et al., 2025a; Hegarty-Craver et al., 2024). Allostatic load also increases progressively with age and cumulative trauma exposure across operational careers (Beckie et al., 2016).

HRV, the beat-to-beat variation in cardiac R-wave-to-R-wave intervals, has emerged as a widely used non-invasive marker of autonomic regulation and psychophysiological state and may contribute to multimodal assessment of cumulative stress burden in cockpit and operational environments (Chaikovsky et al., 2026; Soares et al., 2025), grounded in the concept that healthy adaptive cardiac control depends on dynamic interaction between sympathetic and parasympathetic branches (Costa et al., 2017). Under chronic stress, this regulation may become disrupted, producing altered HRV complexity and impaired autonomic flexibility associated with hyperarousal, impaired executive function, and poorer decision-making (Laborde et al., 2017; Thayer et al., 2009), so that HRV represents both an early measurable signature of ANS state and a potential mechanistic bridge between fighter aviation stressors and cumulative health and performance outcomes (Solano-Atehortua et al., 2026).

In fighter aviation, HRV-relevant stressors include +Gz loading, hypoxia, cognitive workload, emotional arousal, and sleep disruption (Masi et al., 2023; Shang et al., 2025). Cognitive demand produces progressive parasympathetic withdrawal proportional to workload (Koskelo et al., 2024), while acute and cumulative sleep loss systematically alter autonomic regulation (Dolev et al., 2025; Rosa et al., 2022). Baseline HRV may also be associated with subsequent training outcome: pre-training RMSSD was approximately 44% higher in candidates who subsequently completed Air Force Academy training (Kula et al., 2025a, b). In 40 military pilots flying missions in Afghanistan, post-flight autonomic recovery varied by mission profile, with longer flights producing a paradoxical increase in parasympathetic modulation consistent with post-arousal rebound, whereas shorter, higher-intensity missions were associated with sympathetic predominance at landing (Dussault et al., 2009), indicating that HRV should be assessed across the full mission arc rather than from isolated time points.

Significantly elevated heart rate and marked reductions in SDNN and RMSSD occur during live-fire combat training flights relative to pre-flight, and normobaric hypoxia amplifies HRV suppression during high-cognitive-load tasks, indicating that hypoxia and cognitive demand interact to compound autonomic burden (Wittels et al., 2026). HRV indices in student pilots similarly vary across pre-flight, in-flight, and post-flight phases, with distinct inter-individual response patterns (Li Volsi et al., 2023), supporting HRV as a sensitive marker of cardiovascular-autonomic adaptation while reinforcing the need for individualized interpretation. Complementary evidence shows elevated urinary adrenaline after aerobatic and air-to-air combat sorties (Otsuka et al., 2007, 2006), and HRV decline at the cognitive-load threshold beyond which landing accuracy deteriorates (Mansikka et al., 2016), supporting HRV as a dynamic marker of operational strain, particularly when interpreted alongside complementary biomarkers such as cytokines, salivary cortisol, and immunoglobulin A (Lee et al., 2025a; Verissimo Franca de Oliveira et al., 2025).

The value of HRV lies not only in reflecting current state but in tracking adaptation trajectories over time as a dual-use biomarker for both clinical health monitoring and operational performance optimization; the implications for surveillance reform are examined in Section 9. Repeated or continuous HRV monitoring may reveal autonomic changes before overt performance decline in some settings, while AI-augmented analytics may eventually support baseline-relative risk estimation rather than reliance on absolute population thresholds, though the LF/HF ratio should not be overinterpreted in operational settings (Burlacu et al., 2026; Soares et al., 2025). HRV is similarly recognized in aviation and space medicine as a multi-domain health marker and potential biofeedback modality, with individualized baselines and nocturnal RMSSD supporting longitudinal monitoring (Lindsay and Van den Top, 2025), consistent with earlier work showing that decomposing heart rate into sympathetic and parasympathetic components improves workload classification beyond heart rate alone (Backs, 1995).

Cardiovascular surveillance in aircrew should nonetheless extend beyond HRV. An important methodological consideration is confounding imposed by the cockpit environment: cardiac and respiratory changes in real operational settings greatly exceed those seen with laboratory cognitive tasks (Wilson, 1992), and the F-35’s reclined seat, positive-pressure breathing, and anti-G manoeuvres impose respiratory and cardiovascular patterns that substantially affect HRV (Eiken et al., 2007; Hayano and Yuda, 2019; Laborde et al., 2017; Whinnery and Murray, 1990), requiring aircraft-specific reference standards and contextual interpretation rather than negating HRV’s utility (Shen et al., 2026; Wittels et al., 2026). Rhythm and conduction abnormalities documented in aircrew underscore the need to incorporate structural and electrophysiological endpoints (Guettler et al., 2026), and significant post-flight reductions in brachial artery flow-mediated dilation, alongside transient baseline vasodilation, in 20 Eurofighter Typhoon pilots exposed to up to +9Gz are consistent with an acute endothelial response to high-performance flight, supporting flow-mediated dilation as a non-invasive marker for structured cardiovascular follow-up in high-G aircrew (Nesci et al., 2026).

Electrical bioimpedance, particularly thoracic impedance cardiography (ICG), is an important complementary technology that has been underrepresented in aerospace monitoring discussions. ICG estimates beat-to-beat changes in thoracic electrical impedance to derive stroke volume, cardiac output, systolic time intervals, and related hemodynamic indices; when integrated with ECG and blood-pressure measurements, it can also support estimation of preload- and vascular-resistance-related responses (Sherwood et al., 1990; Newman and Callister, 1999). Its non-invasive, low-power, continuous, and increasingly wearable form factor is attractive for aerospace and remote operational monitoring, where HRV alone cannot distinguish whether autonomic change is accompanied by meaningful alterations in central hemodynamics (Groenendaal et al., 2021). Importantly, ICG should not be treated as a reference-standard cardiac-output method under all conditions. Motion, respiration, electrode placement, body habitus, changes in thoracic geometry, and algorithm choice can affect signal quality and absolute estimates; the aerospace literature therefore supports its greatest near-term value for within-subject trend monitoring and multimodal interpretation rather than stand-alone clinical decision-making (Sherwood et al., 1990; Newman and Callister, 1999).

Several interpretive cautions apply throughout the HRV literature reviewed above and should be read as qualifying every HRV-based claim in this review, not only those in this section. First, reduced HRV is not invariably maladaptive, and elevated HRV or increased vagal indices are not invariably protective: both directions of change can reflect recovery, post-exertional rebound, task-specific parasympathetic engagement, or measurement artefact rather than a stable trait of resilience or strain, so single-timepoint HRV values should not be read as a diagnostic label without reference to an individualized baseline and trajectory. Second, the LF/HF ratio in particular should not be interpreted as a direct index of sympathovagal balance; this interpretation has been increasingly questioned on physiological grounds, and we flag it as a specific point of caution wherever it appears in the cited literature (Burlacu et al., 2026). Third, HRV recordings obtained during short, non-stationary flight phases, such as individual manoeuvres or brief mission segments, require careful validation against longer, steady-state recordings before they can be assumed to reflect the same underlying physiology; frequency-domain indices in particular are sensitive to recording duration and stationarity. Fourth, appropriate preprocessing, including artefact and ectopic-beat correction and, where feasible, respiratory adjustment, is essential to the validity of any HRV metric reported in this literature, and studies vary considerably in how rigorously this is reported. Fifth, and most importantly for the surveillance applications discussed in Section 10, none of the observational studies cited in this review were designed to establish operational decision thresholds; associations between HRV metrics and workload, fatigue, or strain do not by themselves justify a specific cut-off for grounding, intervention, or fitness determination, and we are not aware of any HRV threshold in this literature that has been prospectively validated against an operational outcome in fifth- or sixth-generation aircrew. HRV should accordingly be read throughout this review as a promising and biologically plausible research signal rather than a validated clinical or operational biomarker.

### 3.1 Trainable autonomic control and resilience interventions

A critical, underutilised implication of the HRV-as-resilience framework is that autonomic regulation is modifiable and trainable. In a controlled study of Spanish Air Force fighter pilot trainees, 24 sessions of combined neurofeedback and biofeedback training produced significantly improved HRV, decreased EEG theta/SMR ratios, improved cognitive task accuracy, reduced reaction times, and reduced anxiety (Fuentes-Garcia et al., 2025). In 86 military trainees, a structured mindfulness programme produced a 14.4% improvement in HRV and a 45.7% increase in frontal alpha asymmetry, with alpha asymmetry fully mediating the relationship between HRV improvement and parachute landing performance (Lee et al., 2025b). Collectively, HRV biofeedback, neurofeedback, mindfulness, and structured breathing interventions form a toolkit of trainable autonomic resilience approaches suitable for embedding within pre-conversion training syllabi, with rigorous in-flight validation remaining an important research priority (Little, 2025; Zhang et al., 2020).

## 4 High-G acceleration, OBOGS physiology, unexplained physiological episodes

### 4.1 G-force mechanisms, G-LOC, and countermeasures

High positive gravitational acceleration (+Gz) is one of the defining physiological challenges of high-performance fighter aviation (Burton et al., 1977; Langridge and Cable, 2025; Tutton, 2018). During high-G manoeuvres, hydrostatic forces displace blood toward the lower body, reducing venous return, cardiac output, retinal perfusion, and cerebral blood flow (Ercan, 2021). Without effective countermeasures, many individuals cannot maintain adequate cerebral perfusion above approximately +4–5 Gz, producing a progressive sequence of visual and neurological symptoms: grey-out, blackout, almost-loss of consciousness (A-LOC), and ultimately G-induced loss of consciousness (G-LOC) (Bagshaw, 1990; Goswami et al., 2026).

Physiological monitoring indicates that cerebral oxygenation is closely linked to G-tolerance. Near-infrared spectroscopy shows that cerebral deoxyhaemoglobin correlates with symptom severity during acceleration (Benni et al., 2003), and an approximately 15% reduction in frontal cortical tissue oxygenation index at G-LOC has been reported in 141 pilots, supporting frontal cerebral oxygenation as a potential real-time sentinel (Kurihara et al., 2007). Acceleration onset rate is also critical: gradual- versus rapid-onset G-LOC profiles impose distinct cerebrovascular challenges (Gaffie et al., 1993; Guillaume et al., 1997), and under rapid, high-amplitude onset conditions, compressive and shear stresses at the tentorial notch may interfere with cerebral vessels, increasing ischaemia risk and leaving little time for anticipatory compensation (Guillaume et al., 2002). Performance deficits may begin approximately 7.4 seconds before loss of consciousness and persist for an average of 55.6 seconds afterward (Tripp et al., 2006, 2009), and sustained +Gz exposure may impair memory encoding, suggesting sub-LOC stress can degrade situational-awareness formation during high-G combat manoeuvring (Levin et al., 2007).

Individual vulnerability to oxygenation-related performance degradation is highly variable. SpO_2_ responses during acute hypoxia in air personnel vary significantly with BMI, haemoglobin concentration, smoking status, FEV_1_/FVC ratio, and age, reinforcing that aerospace physiological tolerance is individualized and should inform hypoxia training and risk modelling (Nakdimon et al., 2025; Post et al., 2026). High-G exposure also activates broader neuroendocrine and immune pathways, with salivary cortisol and IgA differing between G-test pass and fail groups (Lee et al., 2025a) and salivary microRNAs proposed as candidate minimally invasive markers of high-G physiological stress (Akiyama et al., 2026). The operational prevalence of G-induced alterations of consciousness underscores the continuing relevance of this risk (Lan et al., 2023): in a survey of 403 UK Armed Forces pilots, 21.6% reported at least one such event, with recency of +Gz exposure rated the most important modifiable risk factor and the G-layoff effect identified as an underappreciated hazard requiring operational controls (Jones et al., 2026), while centrifuge-based dose-response characterization across four baseline acceleration levels provides data relevant to calibrating G-training protocols and return-to-G-exposure decision rules (Britton et al., 2026).

Monitoring and countermeasure development are therefore central to next-generation G-protection. Surface electromyography has been reviewed as a non-invasive, cockpit-compatible method for detecting impending G-LOC through muscle activation patterns (Yang et al., 2026b), and a 117-study scoping review confirmed that anti-G straining manoeuvre proficiency, cardiovascular reflex integrity, and lower-limb muscular strength are among the factors most consistently associated with improved G-tolerance, while identifying major gaps including the near absence of female pilot data and insufficient longitudinal monitoring (Bruno et al., 2026).

The evolution of anti-G protection reflects sustained efforts to extend human tolerance near physiological limits, and it is useful to distinguish established countermeasures with a long operational record from emerging approaches that remain under development. Canada was central from the earliest phase: Wilbur Franks developed the Franks Flying Suit in 1939 through the University of Toronto and RCAF aviation medicine programme, among the first operational anti-G suits, and built Canada’s first human centrifuge to evaluate protection under combat-relevant acceleration conditions (Department of National Defence, 2021; 2023), a lineage that continued through the RCAF Institute of Aviation Medicine, DCIEM, and CFEME, contributing to anti-G suit development, the STInG system for CF-18 operations, and controlled characterization of grey-out, blackout, and G-LOC (Albery and Chelette, 1998; Buick et al., 1995; Hendler et al., 1986; Lalande and Buick, 2009; Sibbald, 2001). The modern anti-G suit, positive-pressure breathing for G protection, and automated Ground Collision Avoidance Systems are all established countermeasures with decades of operational validation, and together aim to preserve cerebral perfusion and prevent loss-of-control events during extreme manoeuvring (Summerfield et al., 2018). By contrast, countermeasures specifically adapted to the complex multi-axis acceleration environments of fifth- and sixth-generation aircraft, including non-Gz force combinations associated with advanced manoeuvrability and thrust-vectoring, remain emerging and largely unvalidated in operational settings, since traditional countermeasures were not originally optimized for these combined-axis profiles (Albery, 2004); the surface-electromyography approach to real-time G-LOC prediction noted above is likewise still at the research stage rather than fielded operationally.

Individual cardiovascular vulnerabilities may further interact with high-G stress, as illustrated by a case of a fighter pilot grounded after a frontal lobe infarction associated with mitral valve prolapse (Gray and Bost, 1988); common fatigue countermeasures such as caffeine and modafinil do not substantially alter G-LOC threshold at operationally relevant doses (Florence et al., 2005), and near-space or suborbital acceleration profiles anticipated in future aerospace operations may introduce atelectasis, neuro-ventilatory uncoupling, and cardiovascular dysrhythmias not fully addressed by current screening frameworks (Ahmed, 2026; Langridge and Cable, 2025). Collectively, high-G exposure is a multidimensional stressor spanning cerebral perfusion, autonomic regulation, neuroendocrine activation, and immune modulation; future health-monitoring systems should move G-protection from an event-based safety paradigm toward a longitudinal model of acceleration resilience integrating exposure history, oxygenation, cardiovascular function, and recovery kinetics.

### 4.2 Unexplained physiological episodes and OBOGS respiratory mechanics

UPEs remain one of the most consequential aeromedical challenges in modern tactical aviation. Since 2007, UPEs have been reported across multiple U.S. Air Force and Navy tactical and training aircraft, with the F/A-18 fleet alone accumulating more than 603 reported events (Elliott and Schmitt, 2019). These episodes occur across the flight envelope and cannot be fully explained by classical hypoxia, decompression sickness, or altitude-related training models alone; aircraft in which pilots breathe pressurized ambient air without complex life-support systems appear relatively spared, supporting the view that UPEs arise from interactions among aircraft systems, breathing mechanics, inspired gas composition, and individual physiological susceptibility (Cohen et al., 2023).

The NATO HFM-274 working group provided current perspectives and a multinational synthesis of hypobaric exposure effects in aviators (Mayes, 2024; Sherman, 2024). A key insight is that hypobaria may affect the brain through mechanisms not reducible to concurrent hypoxia (Rosales et al., 2022; Shaw et al., 2021): non-hypoxic hypobaric decompression produces MRI-detectable brain responses, including reductions in white matter GABA and glutathione consistent with transient loss of neuroprotective capacity, with effects dependent on exposure intensity and duration (Connolly et al., 2023, 2024).

The root-cause framework proposed by Elliott and Schmitt holds that UPEs are unlikely to reflect a single failure mode, but instead emerge from the convergence of multiple sub-threshold respiratory and environmental stressors that collectively erode physiological reserve. These include chronic hyperoxia from on-board oxygen generating systems delivering inspired oxygen fractions of approximately 50-95%; absorption atelectasis promoted by high inspired oxygen, G-force-induced lung compression, and restrictive flight equipment; reductions in vital capacity approaching 30%; increased work of breathing from mask, regulator, and hose resistance; ventilation-perfusion mismatch from G-induced lower-lung shunting; and variable OBOGS oxygen output, particularly in systems with minimal or absent plenum buffering (Elliott and Schmitt, 2019). Individually, these factors may remain below the threshold for overt impairment; in combination, they can narrow the pilot’s physiological margin, producing symptoms that do not map cleanly onto traditional hypoxia checklists. A structured self-report scale for the cognitive, sensory, and affective dimensions of mild hypoxic hypoxia, now validated psychometrically, provides a methodologically consistent instrument for characterising these subtle symptom profiles across aircrew cohorts (Vartanian et al., 2025).

This systems model helps explain why UPEs are difficult to diagnose in flight: symptoms may arise from varying combinations of hypoxia, hyperoxia, hypocapnia, increased breathing resistance, atelectasis, acceleration-related pulmonary effects, anxiety, and fatigue (Konings et al., 2025). The operationally effective response described by Elliott and Schmitt, informally “go to Colorado” (descend below 10,000 ft and breathe ambient air with the mask off when safe), addresses multiple candidate mechanisms simultaneously by restoring a more normal breathing environment (Elliott and Schmitt, 2019). A randomized, double-blind Finnish Air Force Hawk simulator study found that normobaric hypoxia increased minute ventilation dose-dependently and that instrument-landing performance remained impaired 10 minutes after hypoxia emergency procedures, consistent with a “hypoxia hangover” effect (Varis et al., 2022), suggesting hyperventilation and delayed recovery may contribute to persistent performance vulnerability even after apparent correction of oxygenation. Combined hyperventilation and normobaric hypoxia training in an F/A-18 Hornet simulator is similarly feasible and effective for reproducing hypoxia-like symptom profiles, reinforcing that hypoxia training should address breathing control and post-exposure recovery alongside symptom recognition.

The cognitive consequences of oxygenation stress are particularly important for fighter operations. A meta-analysis found that acute hypoxia produces domain-specific effects, with vigilance and psychomotor performance more vulnerable than higher executive functions at moderate hypoxic levels, operationally concerning because early UPE-related degradation may affect threat monitoring while higher-level reasoning remains partially preserved (Bloomfield et al., 2026); even non-symptomatic combat jet training sorties produce measurable perturbations in airway function, diffusing capacity, and systemic oxidative-stress parameters, supporting monitoring approaches that detect subclinical strain rather than only reportable events (Bojahr et al., 2024). Characterizing physiological responses to hypobaric hypoxia across operationally relevant altitude ranges is essential for monitoring-system design: heart rate, blood pressure, and SpO_2_ responses have been documented during simulated 5,000 m hypobaric exposure in healthy male pilots, providing altitude-specific reference data for real-time hypoxia alert thresholds (Thanh et al., 2026), and the HAPP dataset captures SpO_2_, heart rate, respiratory rate, and inspired oxygen concentration across 4,500-7,500 m barometric altitudes, supporting algorithm development for high-altitude flight environments (Jia et al., 2026).

UPEs illustrate the risk of designing life-support systems around isolated engineering specifications. Prevention requires OBOGS architectures providing stable inspired oxygen concentrations, adequate plenum buffering, low breathing resistance, and integrated monitoring of oxygenation, ventilation, and cognitive state (Abou-Donia and Brahmajothi, 2020; de Vries et al., 2025; Shaw and Harrell, 2023), informed by respiratory-mechanics principles and interactions among mask interfaces, anti-G systems, acceleration, and workload.

## 5 Biomolecular pathways of neural stress and physiological adaptation

The field of fluid biomarkers in aerospace medicine is nascent but rapidly advancing (Arjmand et al., 2025; Davis et al., 2021; Seidler et al., 2024). Conventional biomarkers used in military aviation health research, including cortisol, catecholamines, and immunoglobulins, capture important neuroendocrine and immune dimensions of the acute stress response (Abou-Donia et al., 2013; Koltun et al., 2023; Otsuka et al., 2006; Raff et al., 2025; Stefanska et al., 2025). More recently, the field has expanded toward markers of neural strain and injury, vascular endothelial disruption, altered blood-brain barrier (BBB) permeability, and impaired glymphatic clearance, reflecting a growing focus on cumulative brain health and long-term neurological risk in personnel repeatedly exposed to high-performance aerospace environments (**Figure 3**) (Rhind et al., 2024; Rice et al., 2026; Van Cutsem et al., 2026).

**Figure 3:**
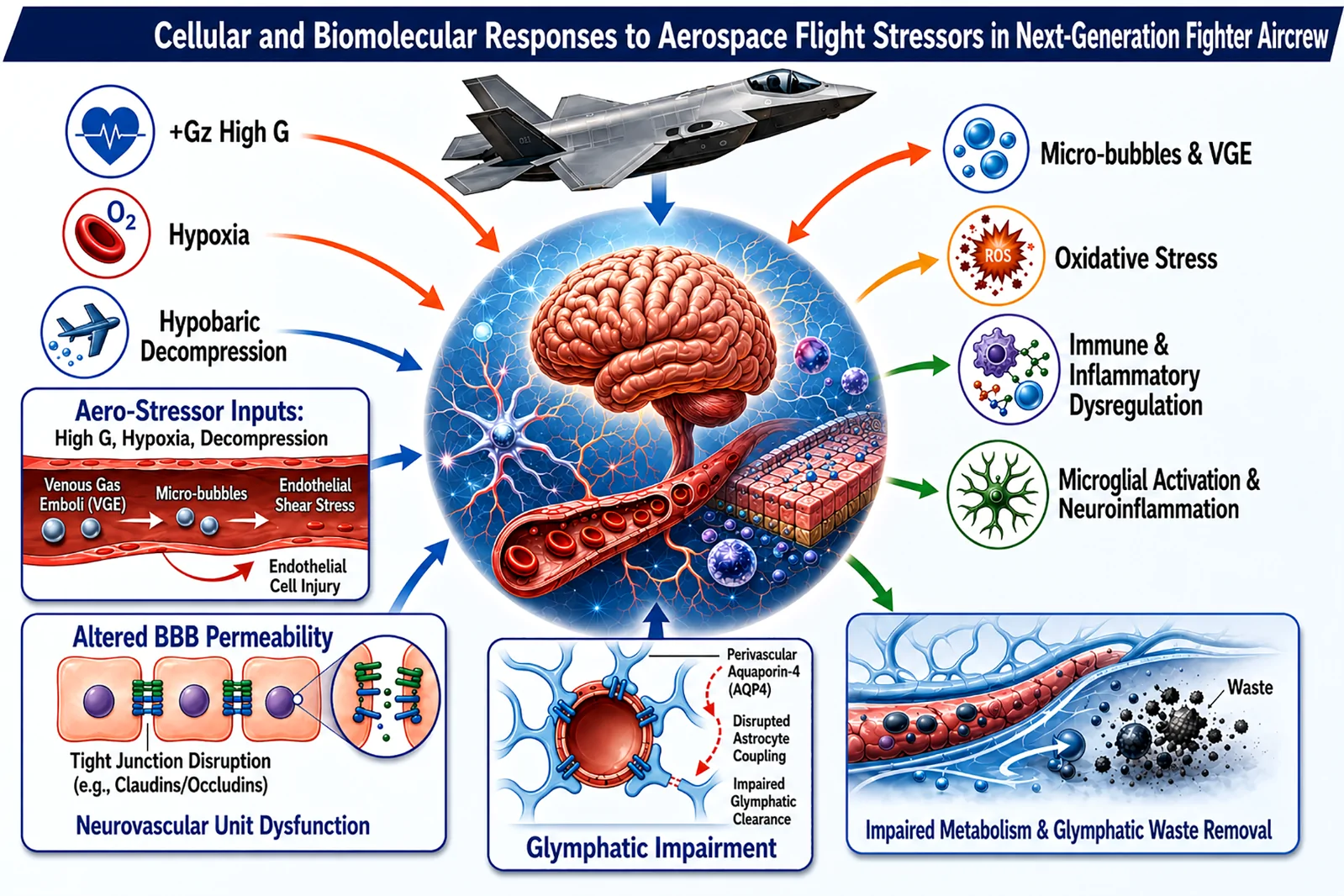
Cellular and biomolecular responses to flight stressors in next-generation fighter aircrew. Extreme aerospace stressors, including sustained +Gz acceleration, intermittent hypoxia, and hypobaric decompression, impose convergent mechanical, metabolic, and vascular strain on the CNS. These exposures may promote venous gas emboli (VGE), microbubble formation, neurovascular shear stress, endothelial injury, altered blood–brain barrier (BBB) permeability, and neurovascular-unit dysfunction. Downstream effects include impaired cerebral perfusion, oxidative stress, inflammatory dysregulation, microglial activation, disrupted astrocyte–vascular coupling, and altered aquaporin-4 (AQP4)-mediated glymphatic clearance. Together, these pathways provide a mechanistic framework by which repeated exposure to high-performance aerospace environments may contribute to cumulative neurobiological stress, altered brain homeostasis, and potential long-term neurological risk in military aviators.

MRI-visible white matter hyperintensities (WMH) currently provide the principal structural imaging window into cumulative neural burden. Previously associated with cumulative exposure in high-performance aircrew (Connolly and Lee, 2015; Summerfield et al., 2018), WMH are widely recognized as markers of small-vessel cerebrovascular injury linked to cognitive decline and dementia risk in broader populations (Ali et al., 2023a, 2023b; Dhana et al., 2022; van Gennip et al., 2024). Foundational evidence comes from high-altitude U-2 pilots, in whom increased WMH burden was associated with significantly lower neurocognitive performance (McGuire et al., 2013, 2016, 2014), a finding since extended to RCAF pilots and aircrew (Ramirez et al., 2025). A recent meta-analysis pooling five MRI-based studies comprising 264 pilots or astronauts and 177 non-flying controls found significantly greater WMH number (mean difference 3.57 lesions) and volume (mean difference 0.09 cm³) in exposed personnel, with no significant heterogeneity across studies (Mohamed et al., 2026); proposed mechanisms include hypoxia-induced neuroinflammation, hypobaric microbubble formation leading to cerebrovascular microemboli, and axonal demyelination, reinforcing the case for longitudinal neuroimaging surveillance across the fighter pilot career span.

Connolly and colleagues provide dose- and context-dependent evidence on hypobaric decompression stress and neurobiological effects. A lower-intensity altitude-chamber programme involving brief, denitrogenated non-hypoxic exposures did not promote new WMH, suggesting such profiles may fall below the threshold for detectable structural injury (Connolly and Lupa, 2021), whereas more intensive hyperoxic hypobaric decompression protocols produced systemic inflammatory and neurological biomarker responses, including IL-6 elevation, persistent CRP and C5a changes, and elevated GFAP, consistent with incomplete biological recovery (Connolly et al., 2024a, 2023). Advanced MRI/MRS demonstrated reductions in subcortical white matter GABA and glutathione dependent on exposure intensity and recovery interval (Connolly et al., 2024b), and high-altitude exposure has similarly been linked with phosphorylated tau deposition, neuroinflammation, and myelin loss (Iacono et al., 2022). The most comprehensive NATO-coordinated observational study of altitude-chamber personnel to date, comparing 19 instructors with 28 controls using high-resolution 3T MRI and diffusion-weighted imaging, found that cumulative chamber exposure time was significantly associated with WMH count, while bilateral reductions in lateral orbitofrontal cortical volume and thickness, regions central to decision-making that co-localize with the predominant frontal WMH distribution, were associated with mild psychomotor slowing despite no overt neuropsychological impairment (Ledderhos et al., 2025), demonstrating that the absence of frank clinical injury should not be interpreted as the absence of measurable neurobiological strain.

Fluid biomarkers provide a complementary and more accessible window into cumulative neural burden. RCAF aviators show significantly elevated plasma GFAP, NF-L, PRDX-6, and T-tau compared with non-aviator military controls, biomarkers associated with glial activation, axonal injury, oxidative stress, and early neurodegenerative processes, whose longitudinal trajectories and relationships to WMH accumulation remain central research priorities (Rhind et al., 2024), and a retrospective cohort study of US Naval aviators quantified the broader epidemiological burden across neurological and psychiatric diagnostic categories, reinforcing the case for proactive longitudinal neurological surveillance (Rice et al., 2026). Inflammatory and autoimmune biomarkers add further insight: MCP-1 has been identified as a serum biomarker of musculoskeletal pain burden in Swiss military aviators, with levels increasing proportionately with back and neck pain frequency (Bron et al., 2024; Kincel et al., 2024), elevated proinflammatory cytokines in military instructor pilots were accompanied by fatigue (Damato et al., 2022), and significantly elevated IgG-class autoantibodies against neural cytoskeletal and glial proteins, including approximately sevenfold increases in anti-tau autoantibodies, have been found in aircrew reporting adverse health effects after ventilation-system air emissions (Abou-Donia et al., 2013, 2014), potentially useful for detecting subclinical trajectories in aircrew with non-specific symptom profiles, including the heterogeneous presentations of UPEs (Abou-Donia and Brahmajothi, 2020).

Structural neuroimaging in pilot-training cohorts suggests that aviation-relevant neurological change can begin early in the career course, including flight-training-associated reductions in grey matter in the left temporal pole, interpreted as adaptive neuroplasticity (Wang et al., 2025b), and gyral morphology changes in the left rostral anterior cingulate cortex over two years of training (Chen et al., 2025a); intracranial abnormalities were identified in 6.5% of 554 Chinese military pilots during career-transition MRI, supporting neuroimaging as a valuable screening component (Summerfield et al., 2018; Zhao et al., 2024). Metabolomics provides a further window into operational exposure and adaptation: NMR-based metabolomics in Brazilian A-29 pilots revealed experience-dependent profiles, with trainees showing greater post-flight salivary glucose increases and instructors showing changes consistent with oxidative-stress adaptation (Verissimo Franca de Oliveira et al., 2025). Oxidative burden from repeated high-G, hypoxia, hyperoxia, decompression, and workload represents a potentially modifiable long-term risk (Bojahr et al., 2024; De Luca et al., 2009), and identification of BChE Ser198 adducts supports exposure-specific biomarker development for aviation toxicants (Furlong et al., 2025).

Cortisol remains a useful neuroendocrine marker, although interpretation requires caution. Cortisol measured at the end of an airsickness rehabilitation programme was associated with desensitization success in student pilots (Greco et al., 2025), while diurnal salivary cortisol measured in 20 experienced F-35 pilots across a flight-training exercise showed that 35% had no measurable cortisol elevation and that pre-flight cortisol correlated inversely with cumulative flight hours (consistent with HPA habituation), with flight-day awakening cortisol inversely correlated with self-reported post-flight stress (Raff et al., 2025), a dissociation suggesting that cortisol monitoring in isolation may under-detect burden in experienced pilots.

Collectively, neuroimaging, fluid biomarker, metabolomic, oxidative-stress, inflammatory, autoimmune, and neuroendocrine findings indicate that next-generation fighter aviation should be studied as a cumulative neurobiological exposure environment. We emphasize, however, that the biomarkers discussed in this section, including GFAP, NF-L, tau, autoantibodies, inflammatory and oxidative-stress markers, and subthreshold white-matter hyperintensities, should be understood as research candidates rather than as tools ready for individual risk stratification or fitness-to-fly decisions. Substantial uncertainties remain unresolved for essentially all of them: specificity relative to non-aviation causes of neural or inflammatory injury; biological and analytical variability, including assay-to-assay and laboratory-to-laboratory differences; the time course over which each marker rises and clears after a given exposure; confounding by age, sex, and comorbidity; the absence of validated aviation-specific reference ranges; an uncertain and largely untested relationship to operational performance; the risk that population screening would generate incidental findings or overdiagnosis without a clear management pathway; and, for subthreshold MRI changes such as the white-matter hyperintensities discussed above, genuine uncertainty about their clinical meaning in the absence of overt neuropsychological impairment. No single marker is likely to provide sufficient sensitivity or specificity for individual risk stratification on its own, and none of the biomarkers reviewed here has been prospectively validated against an operational or clinical outcome in fifth- or sixth-generation aircrew. The most defensible near-term role for this literature is therefore hypothesis-generating and research-oriented: a longitudinal, multimodal biomarker framework integrating exposure history, MRI-derived structural markers, blood-based neural injury and neurovascular biomarkers, inflammatory and oxidative-stress profiles, and cognitive performance could in principle shift aerospace medicine from retrospective injury detection toward prospective monitoring of neural resilience across the pilot career span, but that shift depends on validation work that has not yet been done, and premature clinical deployment risks both false reassurance and unwarranted grounding decisions.

## 6 Cognitive workload, mental fatigue, and the experience effect

### 6.1 Cognitive architecture of next-generation combat

The cognitive demands of fifth- and sixth-generation combat aviation represent a qualitative shift in the role of the fighter pilot (Gündüz, 2025; Wang et al., 2020). Rather than simply flying the aircraft, the pilot must integrate sensor-fusion outputs from radar, EO/IR, electronic warfare, and data-linked networks; make tactical decisions under severe time pressure; supervise increasingly autonomous systems; and communicate across multiple radio networks, while retaining manual flying proficiency for degraded or automation-failure conditions (Deniel et al., 2025; Gündüz, 2025). Summerfield et al. anticipated this challenge in describing a future pilot responsible not only for a single aircraft but potentially for a “squadron” of unmanned systems, generating information flows that may exceed unaided human cognitive capacity and require real-time monitoring of operator state as a safety prerequisite (Summerfield et al., 2018).

The F-35 illustrates this shift from platform control to information management (Svoboda et al., 2019). Its sensor-fusion architecture delivers substantially greater volumes of tactical information than previous-generation fighters, largely through the panoramic touchscreen display and helmet-mounted display, requiring continuous selection, prioritisation, and action on dynamic information while flying and managing weapons (Clark, 2024; Şengöz, 2026). Current pilots describe the difficulty as absorbing all available information while operating sensors and systems, and knowing where to look at the right time (Osborn, 2021). Fifth-generation aviation thus shifts workload from manual aircraft control toward attentional control, information filtering, working-memory updating, and rapid tactical decision-making (Şengöz, 2026), demands likely amplified under high-tempo or degraded conditions, since stress, fatigue, hypoxia, thermal strain, and high +Gz exposure can impair working memory, attentional flexibility, and decision quality (Bottenheft et al., 2023; Wittels et al., 2024). This workload should be understood not as “more information” in the cockpit, but as a dynamic interaction between information density, display architecture, mission tempo, physiological strain, and individual cognitive reserve (Blacker et al., 2019), under which pilots may maintain apparent control performance while their capacity to monitor peripheral cues, update the tactical picture, or evaluate competing courses of action quietly erodes, a compensated-performance state in which overt performance appears preserved while situational awareness is already degrading (Aminian et al., 2025).

Understanding this environment requires distinguishing three related but mechanistically distinct constructs (Albers et al., 2025; Attiah Alzahrani et al., 2025). Cognitive load is the immediate demand on working memory and attentional resources during task performance (Sale, 2025). Cognitive fatigue reflects cumulative depletion of attentional and executive capacity across sustained effort (Pessiglione et al., 2025). Decision fatigue is partially separable, characterized by progressive degradation in decision quality after repeated executive choices, with greater reliance on heuristics, choice avoidance, or narrowed option evaluation (Pignatiello et al., 2020). These constructs differ in time course, physiological expression, and countermeasure strategy (Kapela, 2026): a pilot may show high cognitive load early in a mission while retaining adequate reserve, yet later develop decision fatigue that impairs risk appraisal even while conventional workload indices appear stable (Rosa et al., 2020). Treating these as a single category of “mental demand” risks obscuring the mechanisms monitoring systems must detect.

Monitoring feasibility is advancing rapidly. EEG-based workload assessment (Chen et al., 2025a), HRV-derived workload indices (Koskelo et al., 2024), and multimodal discrimination of adaptive versus maladaptive stress responses (Zhao et al., 2025) all support real-time cognitive-state monitoring architectures (Dan and Reiner, 2017), with substantial between-pilot variability in such systems reinforcing the need for individualized baselines rather than population-level thresholds (D’Alessandro et al., 2024). Working memory updating, cognitive flexibility, response inhibition, and conflict monitoring contribute differentially to piloting, navigation, and communication performance, with working memory updating showing particularly strong predictive validity for critical decision-making (Enriquez-Geppert et al., 2024); HRV suppression, EEG workload signatures, fixation entropy, and pupillometric changes should accordingly be understood as indirect markers of the attentional and executive resources on which performance depends. Burnout-related cognitive depletion is similarly associated with reduced P3a and P3b event-related potential amplitudes, markers of attentional engagement that may capture early degradation before overt performance failure (Wu et al., 2022), and EEG time- and frequency-domain features reliably discriminate pilot workload states (Yang et al., 2026a). At the autonomic level, HRV in simulated flight is sensitive to the specific demands of manoeuvre phases: climbing, descending, and level turns produce differentiated autonomic profiles, with parasympathetically mediated indices such as RMSSD progressively suppressed as manoeuvre complexity increases (Zhou et al., 2024), a proof-of-concept for wearable-enabled classification of flight-phase-specific workload relevant to tactical manoeuvring (Masi et al., 2023).

Importantly, ecologically relevant studies also show that physiological workload markers are not uniformly discriminative. In 14 experienced male F-16 pilots completing an operationally relevant simulator mission, Reuten et al. (2026) found that subjective mental effort increased across mission segments, yet frontal theta, parietal alpha, heart rate, and electrodermal measures did not systematically distinguish the workload manipulations and explained only 2.2% of the variance in subjective workload. The authors attributed the null physiological result in part to marked inter-individual variability, workload-management strategies, and modest differences between task conditions. This recent negative study is an important counterweight to high classification accuracies reported in more bounded paradigms and reinforces that no single physiological signal should be assumed to provide a context-independent measure of cognitive workload in operational aviation.

Several mechanisms may amplify the operational risk of cognitive fatigue. The opportunity-cost model proposes that fatigue intensifies when alternative actions are perceived as more valuable than the task at hand, producing motivational disengagement precisely when sustained effort is required (Dora et al., 2022), while attentional tunnelling compounds this risk: as cognitive resources decline, pilots may narrow attentional bandwidth to preserve primary task performance while sacrificing monitoring of peripheral information, creating a hazardous compensated-performance state in which workload indices appear manageable while the tactical picture has silently degraded (Flood and Keegan, 2022). Military personnel can also experience objective performance decrements while still rating their own situational awareness as adequate, suggesting impaired metacognition and limiting the reliability of self-report as a safety mechanism (Sekel et al., 2023); objective physiological and behavioural monitoring may therefore become essential for detecting early cognitive-state degradation (Alharasees and Kale, 2024; Kapela, 2026).

Cognitive fatigue may also be forecast before performance degrades, predicted from sleep history and operational-tempo data, shifting the monitoring paradigm from reactive identification toward proactive prevention through scheduling, crew-rest allocation, and adaptive mission planning (Veksler et al., 2025). Training architecture must also account for the longitudinal development of workload tolerance; workload trajectories across military flight training inform graduated syllabi (Biondich, 2026), and adaptive-autonomy testbeds in which AI systems dynamically adjust autonomy levels according to operator cognitive state are directly relevant to sixth-generation aircraft, where the boundary between human and machine decision authority will be increasingly fluid (Paul et al., 2026). As discussed further in Section 9, cognitive fatigue during long-duration missions is domain-selective, with vigilance declining before executive function (Rosa et al., 2022). Sensor fusion and automation similarly reduce manual workload during nominal operations but increase cognitive complexity during abnormal events, an automation paradox in which overreliance may erode manual skill and situational awareness while under-reliance creates excessive workload precisely when time pressure and physiological strain are greatest (Li et al., 2022; Şengöz, 2026).

### 6.2 Pilot training and learning effects: critical physiological moderators

Pilot training, repeated task exposure, and learning-related adaptation are critical moderators of psychophysiological responses to flight demands. Rather than treating “experience” as a simple proxy for resilience, cognitive-behavioural science suggests that performance under stress is shaped by structured learning, cue recognition, attentional control, and the progressive calibration of physiological responses to operationally relevant demands. In 21 Swedish military attack pilots, urinary adrenaline increased with mission condition, and prior flying experience moderated the stress response, with higher baseline adrenaline in experienced pilots potentially reflecting a calibrated sympathetic readiness state rather than nonspecific stress activation (Svensson et al., 1988). More recently, licensed pilots exhibited substantially lower EEG-measured cognitive workload, heart rate, and facial thermal responses than novice and intermediate participants exposed to identical simulated scenarios, including high-stress engine-failure emergencies (Khalaj et al., 2026), suggesting that training and task familiarity reduce the physiological cost of performance, likely by improving attentional efficiency, anticipatory control, and stress-regulation capacity.

A key mechanism is the development of learned cue-action mappings: Recognition-Primed Decision-Making allows expert operators to rapidly match situational cues to stored operational patterns and act without slow deliberative processing (Reale et al., 2023), reflecting not simply accumulated flight hours but the progressive learning of aircraft-specific cues, threat patterns, and recovery actions. This advantage is context-dependent, however. In genuinely novel next-generation scenarios, including unfamiliar sensor-fusion displays, autonomous-system failures, or unexpected automation behaviour, previously learned pattern libraries may be incomplete or poorly matched to the problem; cognitive workload may then rise sharply, and the experience advantage may narrow until new learning occurs (Ho and Burns, 2003; Woltjer et al., 2024; Yıldız, 2024). This is important for CF-35 transition research: legacy-platform expertise may not automatically transfer to next-generation cockpit ecology without deliberate conversion training and adaptive learning support.

The relationship between training exposure and psychophysiological adaptation is also non-linear. Exposing novices to highly difficult scenarios at the outset produced persistently elevated stress across subsequent simulated flights, whereas a graduated training progression supported more favourable adaptation, with intermediate participants showing the greatest proportional improvement between first and second exposures (Khalaj et al., 2026), consistent with cognitive-load and stress-inoculation principles: training must be challenging enough to drive adaptation without overwhelming attentional resources or conditioning maladaptive stress responses (Vartanian et al., 2022). Training effects should therefore be treated as measurable trajectories rather than assumed outcomes: changes in EEG workload indices, HR/HRV, eye tracking, sleep-recovery patterns, and task performance can help determine whether a pilot is adapting, plateauing, or accumulating strain, allowing instructors and aeromedical teams to distinguish productive learning stress from excessive burden.

Experience alone is insufficient to explain cognitive resilience under operational stress: higher trait resilience, aerobic fitness, conscientiousness, and lower neuroticism predict smaller declines in tactical adaptive decision-making under simulated operational stress (Sekel et al., 2023). Psychophysiological readiness should therefore be viewed as a composite of learned cognitive skill, platform-specific adaptation, autonomic regulation, stress tolerance, physical conditioning, and recovery capacity, several of which are measurable, trainable, or modifiable through individualized readiness programmes (Sloan et al., 2021; Ulvr and Rackova, 2025). The central research question is not whether experienced pilots perform better, but how training produces adaptive physiological and cognitive change, how that adaptation transfers to next-generation fighter operations, and how monitoring can help sustain learning, health, and mission readiness across the career span.

### 6.3 Real versus simulated flight: complementary rather than equivalent

Simulators should be considered complementary to, rather than equivalent substitutes for, real flight. Simulators can reproduce many psychophysiological demands of real combat flight: cortisol, SpO_2_, lactate, and respiratory function did not differ significantly between real and simulated combat missions, but real flight produced neuromuscular activation (horizontal jump performance) and different metabolic responses not replicated in simulation (Tornero-Aguilera et al., 2025). Real flights also produce greater HRV changes and higher cognitive anxiety than matched simulator scenarios (Fuentes-Garcia et al., 2021), together supporting a complementary architecture in which simulators serve cognitive, tactical, and procedural proficiency while actual flight and centrifuge exposure address G-tolerance, neuromuscular conditioning, and full psychophysiological coupling. Normobaric hypoxia studies conducted in Hawk and F/A-18 tactical simulators illustrate the value of high-fidelity simulation as an experimental bridge between laboratory physiology and operational aviation: by combining full flight equipment, tactical manoeuvring, realistic cockpit tasks, and blinded gas manipulation, these studies show that simulators can reproduce clinically and operationally meaningful respiratory, cognitive, and performance effects that would be difficult to isolate safely in live flight (Leinonen et al., 2022).

An important caveat is that decision fatigue remains inconsistently defined across the cognitive-performance literature (Maier et al., 2025; Pignatiello et al., 2020), making cross-paradigm comparisons less straightforward than they appear, particularly when translating simulator findings to operational settings where stakes, physiological stressors, and temporal demands differ substantially.

## 7 Sensory stress and multimodal integration

### 7.1 Visual and oculomotor strain

HMDS systems project sensor-fused tactical, navigational, and weapons-related information directly onto the pilot’s visor, enabling off-boresight targeting yet simultaneously imposing visual clutter, attentional tunnelling, and perceptual conflict when displayed information diverges from vestibular and proprioceptive cues (Buck, 2024; Cameron, 2015; Compierchio et al., 2024; Jenkins and Havig, 2015; Newman, 2017; Van der Burg et al., 2025).

Oculomotor fatigue is an important consequence of sustained HMDS and night-vision-enabled operations. AH-64 Apache pilots show significant pre- to post-flight changes in pupil size, constriction latency, and saccadic velocity, with alterations comparable to those following sleep loss and occurring after a single flight exposure (LeDuc et al., 2005). Night vision goggles impose additional visual burdens including post-flight eye strain, retinal after-images, altered depth perception, and visual discomfort (Newman, 2017), and cognitive assessment batteries can be tailored to NVG flight requirements for aircrew selection and proficiency assessment (Lewkowicz et al., 2026). These findings support understanding visual performance in advanced fighter operations as a dynamic psychophysiological function that may degrade with workload, fatigue, sleep loss, hypoxia, and repeated operational exposure.

Eye tracking offers a sensitive, non-invasive approach for quantifying visual attention, oculomotor fatigue, cognitive workload, and physiological strain during flight: pupil diameter indexes task demand and sympathetic arousal, blink rate and duration reflect fatigue and vigilance decrements, fixation entropy reflects attentional allocation, and saccadic velocity may mark oculomotor fatigue (Moore et al., 2023). Oculomotor responses may be among the earliest measurable indicators of hypoxia, suggesting eye-based monitoring could serve as a multi-hazard sentinel for visual fatigue, cognitive overload, and degraded operational readiness (Summerfield et al., 2018). Platform-specific challenges of the F-35 Gen III HMDS, including green glow, double vision, monocular symbology rendering, and image-processing artefacts, may increase visual parsing demands during high-tempo operations (Compierchio et al., 2024); AI-augmented fixation maps offer a countermeasure to guide visual search in data-dense tactical environments (Compierchio et al., 2023), and integrating eye tracking with EEG during F-35-relevant HMD training is feasible for measuring how cognitive resources are allocated across display elements (Carroll et al., 2013).

A further emerging concern is the potential impact of repeated high-G exposure on ocular and retinal microvascular integrity: short-term +Gz exposure during centrifuge training produces measurable changes in retinal thickness and vascular density detectable by optical coherence tomography angiography (Hsu et al., 2025). Because the retina provides a uniquely accessible window into neurovascular and systemic microvascular health (De Cesari et al., 2020), longitudinal retinal imaging may be a valuable component of career-span surveillance, particularly when integrated with visual performance, oculomotor function, and cerebrovascular measures.

### 7.2 Vestibular and spatial orientation stress

Spatial disorientation in flight is one of the most persistent and consequential hazards in military aviation, accounting for an estimated 20-38% of fatal military aviation accidents worldwide (Gillingham and Previc, 1993); in one 21-year analysis of U.S. Air Force data, it contributed to 101 deaths and $2.32 billion in aircraft losses (Gillingham, 1992; Poisson and Miller, 2014). At its core, spatial disorientation reflects a mismatch between the pilot’s perceived and actual orientation, motion, or attitude relative to gravity and the surrounding environment (Bushby and Grimshaw, 2025; Meeks et al., 2018). The vestibular system is intrinsically vulnerable in flight because it evolved to resolve terrestrial patterns of gravity, head motion, and angular acceleration, whereas high-performance aviation routinely imposes accelerative forces, visual conditions, and manoeuvre profiles that violate these assumptions (Baumgartner et al., 2025; Pennings et al., 2020).

A fundamental challenge is that, absent reliable visual or instrument cues, the central nervous system cannot fully distinguish linear acceleration produced by vehicle motion from acceleration caused by gravity (Paillard et al., 2014), creating an inherent ambiguity between tilt and translation whereby acceleration may be misinterpreted as a change in pitch or bank attitude (Stott, 2013). Semicircular canals and otolith organs can therefore be systematically misled during sustained turns, rapid acceleration, deceleration, high-G manoeuvres, or degraded visual conditions, producing powerful illusory percepts that may be difficult or impossible to override voluntarily (Rupert et al., 2023), limitations compounded in next-generation fighter aviation by rapid aircraft response characteristics, night or low-visibility operations, and the need to maintain tactical decision-making under intense time pressure (Blaginin and Lyashedko, 2023).

Next-generation helmet-mounted display systems may both mitigate and exacerbate spatial disorientation risk: sensor-fused visual information can improve external orientation and reduce head-down time, but visual clutter, unstable symbology, latency, and mismatch between displayed imagery and vestibular or proprioceptive cues may also increase risk, particularly during high-G manoeuvres, night operations, and degraded visual environments (Benson, 2006; Newman, 2017; Schnell et al., 2017). Spatial orientation in flight is therefore not purely vestibular; it emerges from continuous integration of visual, vestibular, somatosensory, proprioceptive, cognitive, and instrument-derived cues (Gillingham and Previc, 1993; Stott and Benson, 2016), and when these channels become discordant, the pilot must resolve perceptual ambiguity while simultaneously maintaining aircraft control and mission execution (Bertolini et al., 2023). Advanced aircraft may introduce novel disorientation profiles, such as a proposed “reverse-dip” illusion during night aerial refuelling in an F-15I, in which HUD cues and visual fixation contributed to an unintentional climb toward a tanker (Nakdimon and Gordon, 2025); experimentally, spatial disorientation exposure is accompanied by increased cortical arousal, autonomic activation, and psychological strain in both fighter pilots and medical aircrew (Fuentes-Garcia et al., 2021; Tornero-Aguilera et al., 2022), reinforcing that it is not simply a perceptual error but a whole-system psychophysiological stress response involving sensory conflict, autonomic activation, respiratory and vascular regulation, and degraded cue integration.

Vestibulo-ocular reflex (rVOR) performance is one important marker of spatial-orientation adaptation and resilience: pilots exposed to tactical high-performance flight show selectively reduced vertical rotational rVOR gain, with pathological values more common in experienced fighter pilots than trainees, suggesting vestibular adaptation with implications for gaze stabilization and target acquisition during dynamic flight (Bertolini et al., 2023). Conversely, rotary-wing Naval aviators demonstrate significantly better dynamic visual acuity than non-pilot service members, with the effect uncorrelated with flight hours, suggesting vestibulo-ocular resilience may partly reflect selection factors rather than exposure history alone (Lytle et al., 2026), together supporting individualized vestibular and oculomotor baseline phenotyping, targeted training, and longitudinal monitoring as complements to accumulated flight experience (Thomas et al., 2023).

Because the vestibular system is tightly coupled with autonomic, respiratory, cardiovascular, ocular-motor, and postural control systems, future countermeasures should extend beyond subjective reporting and post-event reconstruction: multimodal monitoring integrating flight-state data, head and eye tracking, respiratory patterning, HRV, and postural dynamics may detect emerging spatial disorientation or sensory conflict before aircraft control is compromised (Boril et al., 2025), a capability particularly valuable in fifth- and sixth-generation aircraft, where pilots must manage highly fused information environments and autonomous teammates while operating near the limits of human sensory integration. Recovery from spatial disorientation depends not only on vestibular tolerance but also on cue recognition, cognitive flexibility, instrument trust, and realistic prior training (Dixon et al., 2026), so training should move beyond isolated vestibular exposure toward high-fidelity simulator scenarios that integrate sensory conflict, HMDS-specific artefacts, workload, degraded visual environments, and stress inoculation (Inderberg et al., 2026). The most effective approach will likely combine improved display design, vestibular and oculomotor phenotyping, scenario-based training, and real-time multimodal monitoring to help preserve orientation, performance, and safety under conditions of accelerating perceptual and cognitive demand (Hinckley, 2024).

### 7.3 Auditory and hearing-related stress

The auditory domain remains an underappreciated stressor in military aviation. Tactical aircraft generate extreme acoustic environments, and supersonic jet noise is a recognized contributor to noise-associated hearing loss in military aircrew (Aubert and McKinley, 2011; Muhr et al., 2019). The F-35A has been reported to produce approximately 145 dB at military power and 149 dB in afterburner, levels approaching or exceeding the human pain threshold of approximately 120–140 dB (Gee et al., 2013; Summerfield et al., 2018; Tam, 2025). Although cockpit structures, helmets, and hearing-protection devices attenuate exposure, pilots remain embedded in a complex acoustic environment shaped by engine noise, airflow, warning tones, radio communications, and mission-system alerts (Shen et al., 2025), so auditory stress should be understood not only as a hearing-conservation issue but as a contributor to cognitive workload, autonomic activation, and operational risk (Masi et al., 2023; Mucci et al., 2021).

Noise-induced hearing injury in fighter aviation is not restricted to overt threshold shift. Continuous, intermittent, and impulse noise exposures may produce auditory fatigue, tinnitus, reduced otoacoustic emission amplitudes, impaired speech-in-noise perception, and progressive high-frequency sensorineural hearing loss, emerging first in the 3–8 kHz range with particular vulnerability around 4–6 kHz, frequencies important for speech intelligibility and warning-tone detection (Kuo et al., 2021; Song et al., 2025). CAF pilot data show progressive high-frequency threshold increases over years of service and higher rates of clinically meaningful hearing loss among those reporting inconsistent hearing-protection use (Smith and Burrell, 2017), with fixed-wing pilots and those with higher flying hours showing greater vulnerability in some cohorts (Al-Omari et al., 2018). Ground staff demonstrate even greater vulnerability than fighter pilots, including elevated pure-tone thresholds and extended high-frequency deficits; extended high-frequency audiometry and otoacoustic emissions appear more sensitive to early or subclinical cochlear injury than conventional pure-tone audiometry alone, with both pilots and ground staff showing reduced otoacoustic-emission ratios across 1–8 kHz after a single flight-mission exposure (Kuo et al., 2021), suggesting conventional audiograms may underestimate the acute and cumulative auditory burden of military aviation.

Experimental work supports a broader psychophysiological interpretation of cockpit noise exposure. Simulated cockpit noise above 70 dB(A) triggers sustained sympathetic activation, reflected by heart rate acceleration and increased skin conductance, alongside EEG changes consistent with altered cognitive control (Gkintoni and Halkiopoulos, 2025; Shen et al., 2025), and recorded fifth-generation fighter cockpit noise alters visual-search and auditory working-memory performance during multitasking, with individual noise sensitivity moderating cognitive efficiency and negative affect (Fox et al., 2025), indicating that cockpit noise imposes a dual cognitive-physiological burden extending beyond peripheral cochlear injury (Shukla et al., 2025). Longer-term consequences may also involve central auditory and broader cognitive networks: long-term noise exposure in fighter pilots is associated with decreased grey matter volume and reduced regional homogeneity in the left hippocampus, correlating with impaired delayed verbal and visual working memory (Cheng et al., 2019), while flight training and aviation experience more broadly are associated with structural and functional alterations in multisensory integration and cognitive flexibility networks (Chen et al., 2025b, 2025), underscoring that auditory stress occurs within a broader neurocognitive system already adapting to the demands of aviation.

Auditory stress also intersects with cardiovascular and neurovascular risk in military aviators (Ba̧czalska et al., 2022). Noise exposure can activate hypothalamic-pituitary-adrenal and sympathetic-adrenal-medullary pathways, contributing to autonomic dysregulation, oxidative stress, endothelial injury, and hypertension (Maculewicz et al., 2023), which may interact with intermittent hypoxia, repeated +Gz exposure, sleep disruption, and occupational stress to produce a cumulative rather than simple additive risk profile (Sebastian et al., 2025), arguing for integrating cardiovascular and autonomic monitoring into hearing-conservation programmes (Molitor et al., 2023).

The cognitive dimension of auditory stress is equally important (Martins, 2016). Fighter pilots must continuously process communications from air traffic control, mission command, wingmen, and onboard systems while filtering engine noise, cockpit acoustics, alerts, and life-support sounds; hearing acuity alone is insufficient, since operationally relevant auditory performance depends on the ability to detect, localize, prioritize, and interpret safety-critical signals under high workload and competing sensory demand (Deniel et al., 2025; Shukla et al., 2025). CAF pilot data suggest that while most pilots report little difficulty hearing audio alerts, a meaningful subset experience communication or alert-audibility challenges when hearing protection and cockpit systems interact (Smith and Burrell, 2017), highlighting a recurring design tension: hearing protection must attenuate hazardous noise without masking mission-critical speech, alarms, or spatial audio cues (Rajguru, 2013). Three-dimensional directional audio for threat cueing, for example spatially localizing a missile-warning tone to the direction of approach, represents a promising innovation for reducing visual workload during time-critical threat response, and hearing thresholds are not significantly compromised at +4 Gz, supporting feasibility under moderate high-G conditions (Sandor et al., 2004). Hearing-aware cockpit design should combine active noise reduction, fit-tested hearing protection, preserved speech intelligibility, and adaptive alert prioritization that avoids both masking and startle.

Collectively, visual, vestibular, and auditory stressors should be viewed as interacting components of a single sensory-integration challenge. In next-generation fighter aircraft, HMDS imagery influences spatial orientation, vestibular conflict increases visual dependence, and auditory alerts compete for attention as fatigue, hypoxia, +Gz exposure, and cognitive workload degrade sensory reliability. Future mitigation should combine aircraft-specific noise mapping, extended high-frequency audiometry, speech-in-noise testing, eye tracking, vestibulo-ocular assessment, and autonomic monitoring; preserving auditory health is inseparable from preserving communication accuracy, cognitive performance, and long-term sensory-neurovascular resilience.

## 8 Fatigue, sleep disruption, and psychological health

Fatigue arising from sleep loss, circadian disruption, and sustained operational demand is among the most prevalent threats to pilot health and flight safety. In long-duration single-pilot Gripen missions, sustained attention declined significantly after approximately seven hours, with executive cognitive functions preserved, a hazardous dissociation in which pilots may continue to appear capable of complex tactical reasoning while losing the vigilance needed to detect unexpected system failures or threats (Rosa et al., 2022).

Sleep deprivation further compounds this risk. Twenty-four hours of sleep deprivation impaired both attention and emotional regulation in Air Force pilots, with three hours of recovery sleep restoring emotional regulation but eight hours required to restore attentional performance (Dolev et al., 2025), with direct implications for crew-rest policies. Rates of insomnia and sleep apnea in military aviators have increased over 16 years, with age, BMI, and operational tempo as key risk factors (Caldwell and Knapik, 2025), and circadian misalignment carries broader health consequences in aviation personnel, including metabolic disruption and reproductive concerns in female aircrew (Stefanska et al., 2025). Delayed drowsiness following normobaric hypoxia refreshment training in an F/A-18 Hornet simulator shows that post-exposure symptoms may emerge after apparent immediate recovery (Varis et al., 2023), supporting the need for post-hypoxia monitoring windows and conservative recovery guidance.

Aviator fatigue cannot be reduced to sleepiness alone. Inflammatory and cytokine-mediated responses to physical duress, including high-G exposure, hyperoxia, and pressurized-gas breathing, may contribute to fatigue independently of sleep loss (Damato et al., 2026), consistent with the allostatic load framework reviewed in Section 3, so scheduling optimization alone is unlikely to be sufficient for pilots exposed to G-force, hypoxia, and combat-related stress. Fatigue also varies qualitatively and quantitatively across flight types, supporting flight-type-specific fatigue risk management (Erme, 2026), while resource, procedural, and compliance gaps in Air Force fatigue risk management system implementation highlight the persistent gap between fatigue policy and operational reality (Hadi et al., 2026).

Psychological health, burnout, and resilience are therefore central determinants of both short-term performance and long-term career sustainability. Psychophysiological readiness has been formalized as an assessable component of tactical aviation professionalism, incorporating emotional-nervous stability, fatigue resistance, sensorimotor reaction speed, and capacity for rapid resource mobilization (Roman, 2021). HRV-based resilience assessment provides an objective biological complement to subjective resilience scales, and evidence that resilience moderates the relationship between occupational stressors and psychological distress in pilots suggests that resilience-building interventions should be embedded within training pipelines rather than delivered only as *ad hoc* wellness programmes. For next-generation aviation careers, fatigue and psychological health should be treated as integrated readiness domains requiring continuous monitoring, individualized baselines, and proactive intervention.

## 9 Limitations of current aircrew health surveillance

The central argument of this review is that current aircrew health surveillance paradigms have not kept pace with the psychophysiological demands of next-generation fighter aviation. These paradigms share four structural limitations that should be explicitly addressed in any future framework (Marqueze et al., 2023).

First, current surveillance is episodic rather than continuous. Annual or periodic medical examinations provide a point-in-time snapshot, entirely missing the dynamic psychophysiological trajectories that unfold across training cycles, operational deployments, and career transitions. A pilot may pass an annual examination in excellent apparent health while carrying a trajectory of progressive HRV suppression, accumulating WMH burden, or declining cervical range of motion that will not manifest as a clinical finding until significant damage has occurred. Significant bilateral orbitofrontal cortical volumetric reduction has been documented in altitude-chamber personnel who remained fully mission-capable and passed all neuropsychological thresholds, illustrating precisely this problem: structural brain changes of potential operational significance can accumulate to detectability before any conventional examination would flag them (Ledderhos et al., 2025).

Second, current surveillance lacks individualized baselines. Without knowledge of a pilot’s characteristic HRV pattern, cognitive performance profile, or biomarker baseline, meaningful deviations cannot be reliably detected, since a finding representing significant suppression for one individual may fall within normal population ranges for another. Population-level reference ranges are insufficient for detecting individual-level trajectories that predict future impairment.

Third, current surveillance is insufficiently sensitive to subclinical change. Episodic measurement combined with population-level reference standards means that meaningful early changes in psychophysiological state, which may appear months or years before clinical thresholds are crossed, go undetected. This is particularly concerning for cumulative exposures (repeated G-force, career-long hypoxia, chronic noise) where harm may be irreversible by the time it is clinically detectable.

Fourth, current surveillance is platform-centric rather than pilot-centric. Health monitoring is largely reactive and tied to aircraft-specific investigations, as with the UPE crisis, where the focus was on finding aircraft defects rather than characterising the pilot’s physiological state. A pilot-centric approach would track the individual pilot’s psychophysiological state longitudinally across aircraft type conversions, operational roles, and career phases, enabling detection of cumulative stressor effects that cross platform boundaries.

## 10 Toward next-generation psychophysiological monitoring

### 10.1 Multimodal sensing ecosystems

The technical foundation for next-generation psychophysiological monitoring is rapidly emerging (**Figure 4**). Wearable, embedded, and cockpit-integrated systems can acquire ECG/HRV, photoplethysmography, electrical bioimpedance/impedance cardiography, EEG, fNIRS, electrodermal activity, respiration, temperature, SpO2, EMG, blood-pressure surrogates, and movement-related signals (Calado et al., 2025; Sobotnicka et al., 2025). Such time-resolved measures may complement behaviour and self-report when workload, fatigue, hypoxia, +Gz stress, or sensory demands change rapidly.

**Figure 4:**
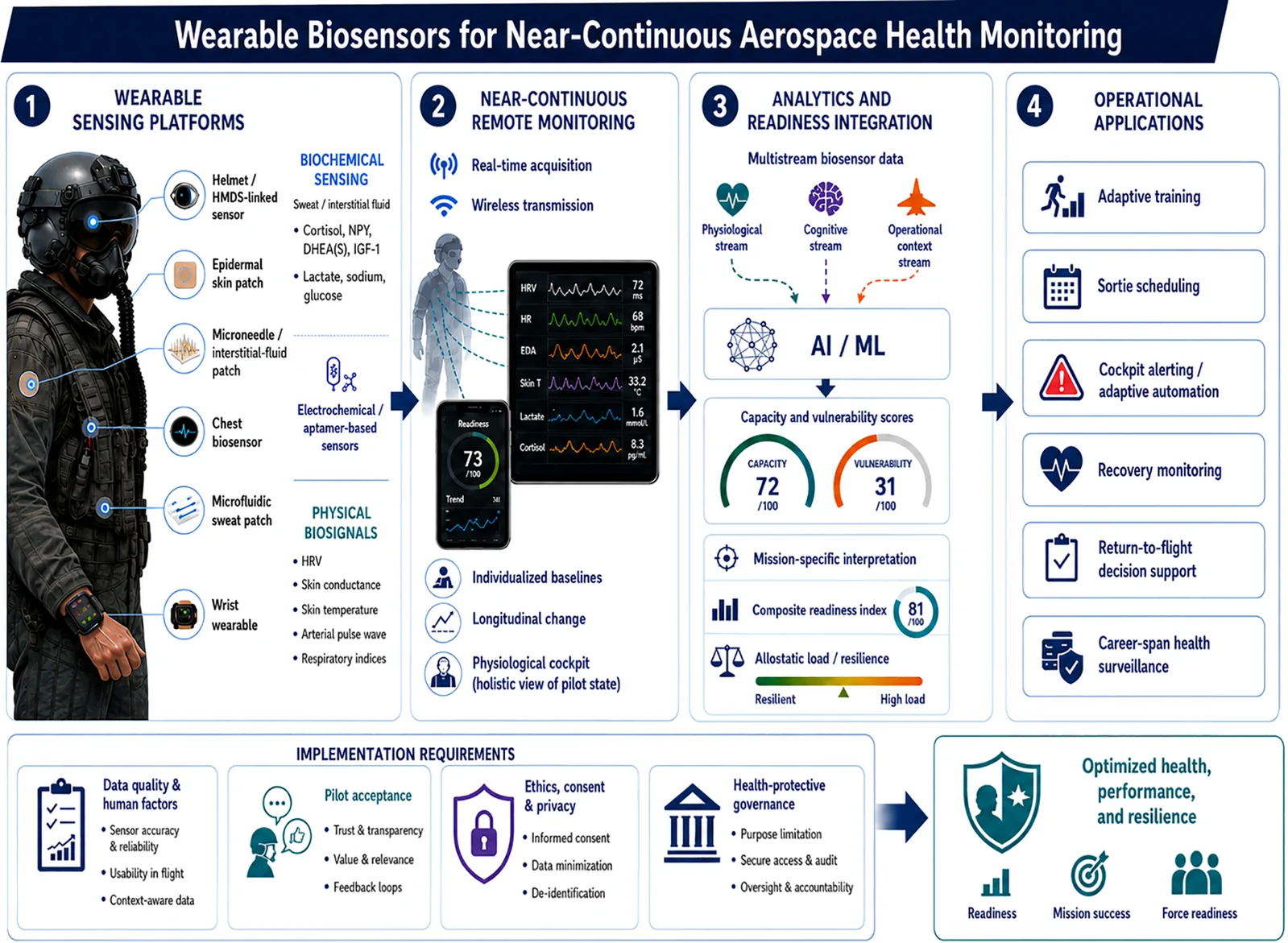
Wearable biosensors for near-continuous aerospace health monitoring. Illustrates a proposed wearable biosensor ecosystem for next-generation aerospace health monitoring. Helmet/HMDS-linked sensors, epidermal and microfluidic patches, microneedle/interstitial-fluid sensors, chest biosensors, and wrist wearables can capture biochemical markers together with physical biosignals. Wireless integration of these data streams enables individualized baselines, longitudinal tracking, AI/ML-derived readiness indices, vulnerability scoring, and estimates of allostatic load and resilience. Operational applications include adaptive training, sortie scheduling, cockpit alerting, recovery monitoring, return-to-flight decision support, and career-span health surveillance. AI/ML, artificial intelligence/machine learning; DHEA(S), dehydroepiandrosterone/dehydroepiandrosterone sulfate; EEG, electroencephalography; fNIRS, functional near-infrared spectroscopy; HMDS, helmet-mounted display system; HRV, heart rate variability; IGF-1, insulin-like growth factor-1; NPY, neuropeptide-Y.

Wearable neurotechnologies add complementary information: EEG provides high-temporal-resolution indices relevant to workload, vigilance, and fatigue, whereas fNIRS captures cortical oxygenation and prefrontal activation during executive tasks (Ayaz, 2025; Izzetoglu and Richards, 2019; Pongsakornsathien et al., 2022). Their main near-term value is comparative and research-oriented, including testing whether new cockpit systems reduce operator burden or redistribute it (Belloni and Curșeu, 2026).

The sensing ecosystem extends beyond brain monitoring. Smart garments can integrate flexible electrodes, strain, thermal, and bioimpedance sensors (Groenendaal et al., 2021; Ma et al., 2025); HMDS-linked systems can capture gaze, blink, pupil, and oculomotor dynamics (Compierchio et al., 2023); and epidermal, microfluidic, or microneedle platforms can sample sweat or interstitial-fluid markers relevant to stress and metabolism (Sim et al., 2022; Spinelli et al., 2025; Wu et al., 2024; Gulgosteren et al., 2025). Compact multimodal skin-interfaced systems have demonstrated coordinated capture of cardiac, respiratory, electrodermal, thermal, and movement responses under several stress conditions, although transfer to high-G flight requires separate validation (Kim et al., 2026).

A central lesson from real-world wearables is that signals are highly context dependent. HRV, EDA, respiration, skin temperature, and PPG-derived indices vary with posture, movement, heat, altitude, hydration, circadian state, workload, and emotion (Norman et al., 2026). Fighter monitoring should therefore pair biometrics with exposure and platform context, including acceleration, flight phase, cockpit temperature/noise/vibration, barometric pressure, and oxygen-system status, rather than interpreting any single signal as a specific marker of workload or readiness (Guarnaccia et al., 2026).

Cockpit-embedded optical, acoustic, and thermal sensors may add facial temperature, breathing-mask, voice, and communication features without increasing body-worn burden (Belloni and Curșeu, 2026; Gnerre, 2026; Liu et al., 2025). Their value will depend on robust handling of motion artefact, sensor displacement, data loss, and operational context (Alharasees et al., 2026).

Collectively, these approaches support the concept of a ‘physiological cockpit’ in which wearable, embedded, and platform-derived signals provide a more complete view of pilot state (Summerfield et al., 2018). The near-term priority is not sensor proliferation, but identification of combinations that remain reliable, interpretable, and actionable across simulator, centrifuge, and live-flight conditions. Individualized baselines may improve sensitivity to within-person change, but deployment also requires the validation, governance, and pilot-trust safeguards discussed below.

### 10.2 Advanced analytics and AI/ML

AI/ML approaches are increasingly used to translate multimodal physiology into estimates of workload, stress, fatigue, or operator state (Johnston et al., 2026; Molloy et al., 2026). Proof-of-concept studies have reported 83-91% eustress/distress classification from fNIRS-ECG fusion, 91.67% workload classification from live-flight EEG, multimodal behavioural-state recognition in an F-35 simulator, and discrimination of experience-related physiological signatures (Khalaj et al., 2026; Li et al., 2022; Taheri Gorji et al., 2023; Zhao et al., 2025).

Some studies extend beyond simulation. Shang et al. (2025), for example, reported 95.56% high-versus-low stress classification from ECG and respiratory features in 32 pilots during combat-simulated flight, while other work has explored cross-condition generalization and multimodal training evaluation (Pontiggia et al., 2026; Zhang et al., 2026). These results show that physiological signals contain classifiable information, but not that the resulting models are ready for safety-critical use.

Reported accuracies should be interpreted cautiously. Small homogeneous samples, class imbalance, and train-test leakage can inflate performance; subject-dependent models are easier than subject-independent models, and internal cross-validation is weaker than external validation on new pilots, platforms, and missions. Simulator models may also fail under the noisier physiology of real flight, and binary classification is much simpler than multiclass operational detection. Accordingly, the reported values are best treated as proof-of-concept, not estimates of prospective utility. Operational translation requires subject-independent and external validation, calibration, explainability, uncertainty reporting, and explicit characterization of false-positive and false-negative consequences before an output can influence training or aeromedical decisions.

### 10.3 Evidence maturity, validation, and operational readiness

A central implementation challenge is that the technologies discussed in this review are not at the same level of scientific or operational maturity. Established physiological measurements such as ECG, pulse oximetry, standard blood-pressure assessment, and laboratory-grade impedance cardiography have well-characterized measurement principles and broad clinical or research validation, although their performance during high-G, vibration, pressure breathing, and prolonged cockpit use still requires context-specific verification. HRV is similarly mature as a derived autonomic measure, but aerospace-specific decision thresholds remain incompletely standardized. In contrast, EEG, fNIRS, eye tracking, EDA, smart textiles, and wearable biochemical sensing have substantial experimental support but more limited evidence for routine use during unrestricted operational flight. AI/ML-based readiness prediction, closed-loop adaptive automation, and continuous molecular sensing should therefore be regarded as promising future capabilities rather than established operational standards.

Across modalities, the principal translational barriers are reproducibility, standardization, ecological validity, and multimodal integration. Motion and vibration artefacts, sweat and thermal effects, electrode or optode displacement, variable perfusion, respiratory mechanics, pressure equipment, and electromagnetic or optical interference can degrade signal quality; synchronization errors and missing data become increasingly important as sensor number increases. Inter-individual variability also limits population-level thresholds, while proprietary preprocessing and device-specific algorithms can impede reproducibility across platforms. These limitations argue for staged validation: bench and laboratory characterization, simulator and centrifuge testing, matched in-flight validation against reference methods, and only then prospective assessment of whether a metric improves health, safety, training, or recovery decisions.

Accordingly, the monitoring architecture proposed here should be interpreted as a research and capability-development framework rather than a claim that all components are ready for routine deployment. Near-term systems should privilege validated measurements with clear physiological meaning and use experimental sensors or algorithms as adjuncts until their incremental value is demonstrated. This distinction is especially important for AI-enabled decision support: algorithmic outputs should augment, not replace, aeromedical judgment, and should be introduced only with transparent uncertainty, human oversight, and predefined failure-management procedures.

### 10.4 Operational integration: supporting adaptive training, recovery, decision support

The value of psychophysiological monitoring lies in the decisions it can validly support. In training, synchronized physiological data may augment simulator debriefing and help identify overload or fatigue; in operations, validated trends might eventually inform scheduling and recovery; and in-cockpit applications could, in principle, support alerts or adaptive task allocation (Robinson et al., 2026; Shaw and Harrell, 2023; Summerfield et al., 2018). These uses have very different validation and safety requirements and should not be treated as a single level of readiness.

Longitudinal monitoring may also characterize recovery and allostatic burden. Neuroendocrine markers such as cortisol, NPY, DHEA(S), and IGF-1 may be informative when interpreted as response and recovery trajectories rather than isolated concentrations, but substantial variation from genetics, sex, training, and context limits population thresholds (Sim et al., 2022; Van Cutsem et al., 2026).

Multistream data become useful only when translated into interpretable, validated information. Proposed military readiness frameworks combine physical, psycho-emotional, cognitive, and contextual data into individualized capacity or vulnerability estimates (de Vries et al., 2025), while emerging knowledge-graph and language-model approaches may help structure relationships between physiological state and mission demands (Okere and Balyan, 2025). These remain research architectures rather than validated readiness scores.

Any future composite system should demonstrate incremental value beyond simpler measures and be designed around operational constraints, data governance, and user acceptance (Coffey et al., 2026; Strong et al., 2026). Consent, ownership, access, and protection against punitive repurposing of health data are prerequisites for pilot trust, not secondary implementation details (Eagan, 2026).

Monitoring is most defensible when linked to evidence-based intervention. Neurofeedback and HRV biofeedback may improve self-regulation and anxiety control in pilot trainees (Fuentes-Garcia et al., 2025), whereas altitude decompression sickness already has defined grounding intervals in some air forces, illustrating a more mature decision framework. Comparable, standardized return-to-flight criteria for UPEs and other emerging hazards remain a research and policy priority (Mago, 2025; Tasiou et al., 2026).

Because research findings should not be conflated with fielded capability, we propose a three-stage translation pathway. Stage 1 is research use: establish feasibility, repeatability, individualized reference behaviour, and relationships with independent performance or health outcomes, with no operational consequence attached to an individual result. Stage 2 is surveillance use: validated measures track longitudinal trends for contextual review by human clinicians or supervisors. Stage 3 is decision support: only after prospective validation, false-positive/false-negative characterization, and governance requirements are satisfied could a measure contribute to training progression, go/no-go, or return-to-flight decisions, and then only as one input to human judgment. To our knowledge, none of the emerging multimodal or AI-enabled systems reviewed here has progressed beyond Stage 1 in fifth- or sixth-generation fighter populations.

### 10.5 Technical and operational limitations of continuous monitoring

The ‘physiological cockpit’ concept currently outpaces deployment readiness. High-G manoeuvring, vibration, anti-G straining, and pressure-suit, helmet, or oxygen-mask interfaces can contaminate signals or displace skin-contact sensors. Electromagnetic compatibility, secure data handling, and airworthiness certification are also unresolved for many candidate systems. These constraints mean that laboratory performance cannot be assumed to persist during unrestricted operational flight.

At the decision level, both false positives and false negatives matter. A high-sensitivity system may generate nuisance alerts that add workload; a high-specificity system may miss genuine deterioration. Model drift, platform and sensor changes, missing data, and cross-population transfer further complicate performance. Systems must therefore fail transparently and safely, quantify uncertainty, and be revalidated when hardware, software, aircraft, or operating context changes.

Continuous monitoring also creates governance questions: who receives an alert, who is authorized to act, and who is accountable for erroneous grounding, mission interruption, or a missed event? These consequences are operationally and professionally significant. Together with privacy, consent, ownership, and cybersecurity concerns, they support our view that continuous physiological monitoring remains a promising research direction rather than a deployment-ready capability.

## 11 Future research directions and solution-oriented studies

Collectively, the evidence reviewed supports a paradigm shift in how pilot health and performance are conceptualised in next-generation fighter aviation. Rather than isolated stressors producing discrete outcomes, fifth- and sixth-generation fighter operations impose interacting psychophysiological loads that accumulate across time, mission phase, platform transition, and individual susceptibility, converging on shared regulatory systems including autonomic control, cerebrovascular regulation, neurocognitive processing, sensory integration, endocrine-immune signalling, sleep-recovery dynamics, and musculoskeletal resilience.

A key implication is that preserved task performance does not necessarily indicate absence of physiological strain. Autonomic, cognitive, sensory, and neural responses may initially function adaptively to maintain operational effectiveness, the “compensated performance” phenomenon described above, but these compensatory mechanisms have finite capacity: a pilot may continue to meet mission demands while exhibiting increased physiological cost, reduced recovery reserve, degraded sleep, elevated allostatic load, or subclinical biomarkers of neural, vascular, inflammatory, or musculoskeletal strain that, over time, may erode resilience and increase vulnerability to acute performance breakdown, spatial disorientation, unexplained physiological episodes, or longer-term health consequences.

The critical research challenge is therefore not simply identifying gross impairment after it becomes operationally obvious, but detecting early deviations in individual adaptation trajectories before performance degradation or clinical symptoms emerge. Conventional surveillance, periodic medical examinations, post-event reporting, standard audiometry, self-reported fatigue, or routine fitness assessments, is not designed to capture dynamic, subclinical changes of this kind. Future research should therefore move from cross-sectional “snapshot” assessment toward longitudinal, individualized, multimodal monitoring capable of distinguishing adaptive response from emerging vulnerability (**Figure 5**), beginning with career-spanning trajectory science: baseline phenotyping performed before major platform transition, covering autonomic and cardiovascular regulation, cognitive performance, oculomotor and vestibular function, auditory performance, sleep and recovery patterns, musculoskeletal status, and biochemical markers, with repeated assessments during conversion training, high-G exposure, and operational deployments.

**Figure 5:**
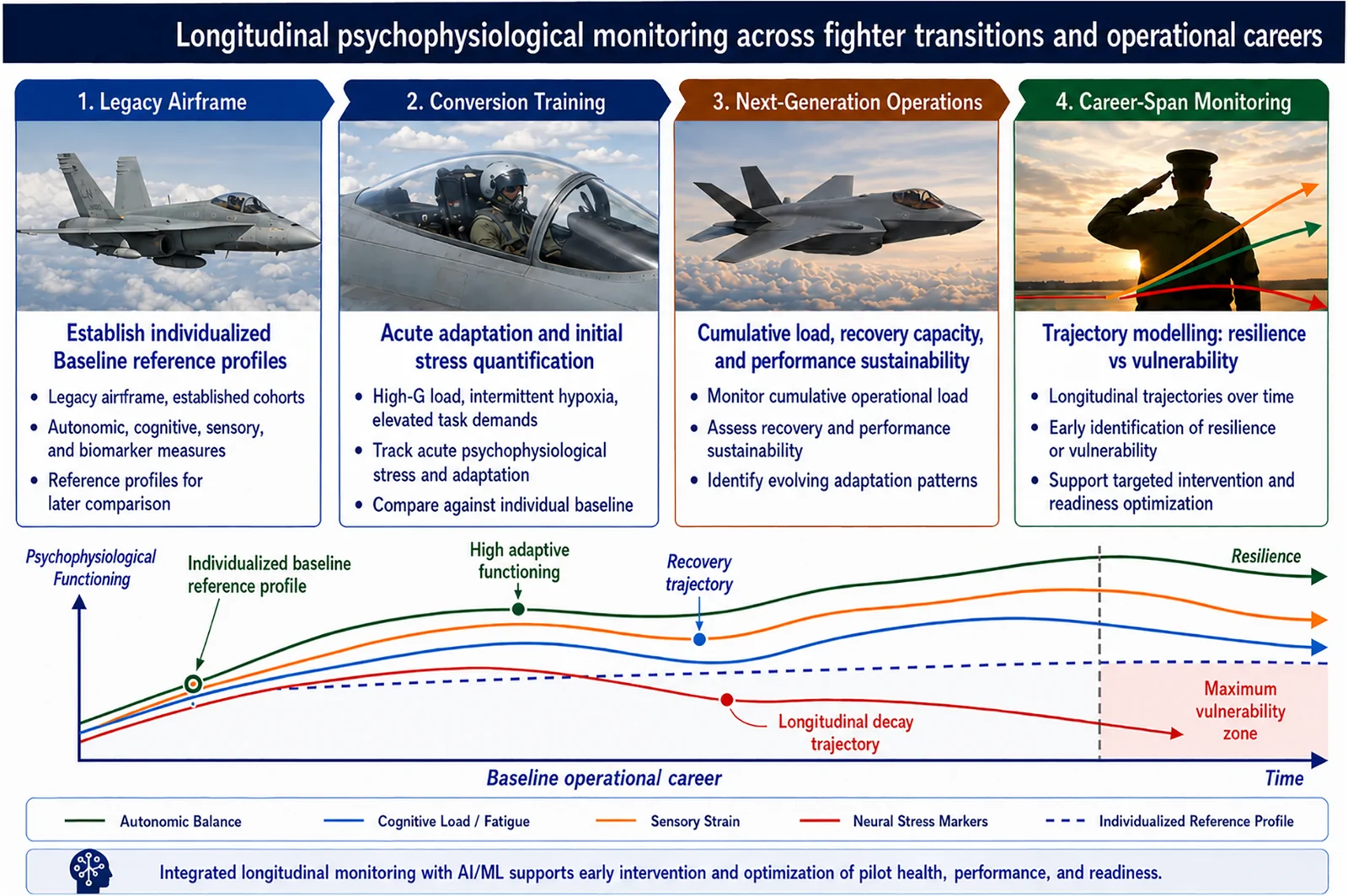
Psychophysiological monitoring across fighter aircraft transitions and operational careers. This schematic illustrates a framework for longitudinal, multimodal monitoring as fighter pilots transition from legacy airframes through conversion training to next-generation operations and across their careers. Baseline assessments establish individualized reference profiles across autonomic regulation, cognition, sensory processing, sleep–recovery dynamics, musculoskeletal status, and physiological biomarkers. Conversion training enables assessment of acute adaptation to high-G loading, intermittent hypoxia, HMDS demands, and increased workload, while operational service captures cumulative exposure burden, recovery capacity, and performance sustainability. Over time, trajectories may diverge toward resilience, compensated strain, or emerging vulnerability. Integration of individualized baselines with AI/ML analytics can support early detection of subclinical strain, characterization of adaptation patterns, and proactive optimisation of pilot health, performance, and long-term readiness. The trajectories shown are hypothetical and are intended to illustrate potential patterns of adaptation and vulnerability rather than empirical longitudinal data or validated clinical thresholds.

Solution-oriented studies should link exposure, biology, function, and operational context, pairing aircraft-derived exposure metrics (+Gz dose, sortie duration, flight phase, helmet configuration, oxygen-system events, cockpit temperature, noise, vibration) with wearable physiology, cognitive testing, sensory-system assessment, and blood-, saliva-, sweat-, or interstitial-fluid biomarkers, to determine which exposure combinations predict transient strain, delayed recovery, or persistent maladaptation, and to identify protective factors such as autonomic flexibility, vestibulo-ocular stability, physical conditioning, and cognitive reserve. Artificial intelligence and machine-learning methods will likely be essential for translating these complex data streams into actionable insight, though the goal should not be generic prediction models built around population averages: the most useful systems will combine population-level risk modelling with individualized baselines, flagging early signatures of overload, delayed recovery, or vulnerability to hazards such as spatial disorientation, hypoxia-related cognitive decrement, and musculoskeletal injury, and should be interpretable rather than functioning as opaque “black-box” tools.

Future research should also move beyond risk detection toward intervention testing, pairing longitudinal monitoring with studies evaluating countermeasures such as individualized G-tolerance training, vestibular and oculomotor conditioning, neck strengthening, sleep and fatigue-management programmes, and cockpit-interface refinements, to determine not only who is becoming vulnerable but which intervention restores resilience most effectively. Implementation science will be critical: monitoring systems will only succeed if trusted, usable, and governed by clear health-protective policies, so pilot acceptability, data ownership, consent, and safeguards against punitive use should be treated as core research outcomes, not afterthoughts. The ultimate objective is a learning health-and-performance system that protects individual pilots, informs platform and training design, and sustains operational capability across the career span.

### 11.1 Longitudinal psychophysiological cohort studies

The most urgent research gap is the absence of prospective longitudinal cohorts tracking fighter pilots’ psychophysiological state across training, conversion, and operational phases on fifth- and sixth-generation platforms, which would allow researchers to distinguish pilots who develop resilient adaptation profiles from those showing compensated strain or emerging vulnerability. Serial assessments should include neuroimaging where feasible, musculoskeletal assessment, cardiovascular and autonomic profiling, biomarker characterization, cognitive performance testing, sleep-recovery monitoring, and detailed exposure history, providing the evidence base needed to define health-protection standards and intervention thresholds for next-generation aircrew.

### 11.2 Integrated simulator-in-flight study design

Future studies should combine the experimental control of simulation with the ecological validity of live flight. Complementary findings on domains of equivalence and divergence between simulator and real-flight physiology (Fuentes-Garcia et al., 2021; Tornero-Aguilera et al., 2025) argue for hybrid designs pairing controlled simulator manipulation with matched in-flight measurement. Stressor-interaction mapping will be particularly valuable: rather than testing high-G loading, hypoxia, cognitive workload, and acoustic stress in isolation, future studies should systematically vary combinations of these stressors to characterize their additive, synergistic, or threshold-dependent effects, supported by simulator infrastructure integrating synchronised flight data with physiological, cognitive, and environmental data streams (Ozdemir and Savas, 2026).

### 11.3 Sensory-specific risk and mitigation studies

Sensory-system research remains a major priority because visual, vestibular, and auditory demands are central to next-generation fighter performance but are often studied separately. The auditory domain remains comparatively underdeveloped, with limited evidence characterising auditory fatigue trajectories across realistic sortie profiles or how hearing protection affects speech intelligibility under combat-relevant conditions (Kuo et al., 2021; Song et al., 2025). Comparable gaps exist in the visual domain, where evidence from night-vision-goggle use may not fully generalize to conformal augmented-reality displays or sensor-fused symbology (Mędrzycka et al., 2025; Wang et al., 2025a). Vestibular and spatial-orientation mitigation studies should also be prioritized, evaluating vestibulo-ocular adaptation and recovery from sensory conflict across simulator, centrifuge, and live-flight contexts, and testing whether targeted vestibular training, oculomotor conditioning, and real-time gaze and head-motion monitoring can reduce spatial-disorientation risk while preserving tactical performance.

### 11.4 Personalised monitoring and adaptive training

Physiological monitoring may help identify overload, fatigue, or maladaptive adaptation before overt impairment, but translation into routine practice remains limited by ecological validity, sensor intrusiveness, pilot acceptance, and uncertainty about how outputs should change decisions (Belloni and Curșeu, 2026; Woltjer et al., 2024). Future work should therefore test longitudinal, *in-situ*, multimodal models against independent performance and health outcomes, with interpretable analytics calibrated to within-person change rather than population thresholds alone. Adaptive training systems that adjust scenario difficulty according to validated indicators of operator state are a promising research direction (Khalaj et al., 2026; Summerfield et al., 2018), but should be evaluated first as supervised training tools rather than assumed to be ready for operational automation.

### 11.5 Cross-domain and allied force collaboration

The psychophysiological challenges of next-generation fighter aviation are shared across allied air forces and are unlikely to be resolved efficiently through isolated national programmes. Common data standards, interoperable assessment protocols, and shared analytic approaches would support pooled analyses and reduce duplication (Mills et al., 2026), particularly for rare outcomes and platform-transition effects difficult to study within a single national cohort. Cross-domain alignment with space medicine may also provide useful methodological guidance, since spaceflight research has developed mature approaches for continuous, individualized physiological monitoring in small populations operating in extreme, high-consequence environments (Dino et al., 2026; Summerfield et al., 2018). Ultimately, future research should converge on a shared objective: to move next-generation fighter aviation from reactive aeromedical surveillance toward predictive, preventive, and performance-sustaining health systems that identify vulnerability before failure and protect the long-term health of pilots across their careers.

## 12 Implications for military aviation and air forces of the future

The evidence synthesized in this review carries clear implications for military aviation policy, platform development, and force health protection that extend beyond the academic literature.

For platform development, the evidence reviewed here suggests that aeromedical and human performance expertise should be embedded in aircraft development programmes from first concept through full operational capability. Life support system design, cockpit acoustic environments, HMD systems, and escape systems must each incorporate iterative human physiological testing as a design-gate requirement. The lesson of OBOGS, that a technically sound engineering solution can create unforeseen physiological hazards when integrated with the full complexity of high-G, high-altitude, high-workload operations, applies across every life-critical system in next-generation aircraft.

For training and personnel development, objective psychophysiological measures, including HRV-based resilience indices and experience-related physiological signatures, may complement existing monitoring and support frameworks (Khalaj et al., 2026; Kula et al., 2025a). These should not substitute established aeromedical standards but may help identify trainees who could benefit from targeted support or adjusted progression (Ulvr and Rackova, 2025); graduated syllabi that progressively introduce stressor complexity, informed by physiological monitoring, may optimize adaptation and reduce avoidable training burden.

For operational health and surveillance, current evidence supports more longitudinal, individualized, and pilot-centred monitoring approaches. Episodic assessments remain essential for aeromedical governance, but continuous or repeated monitoring interpreted against individual baselines, rather than population norms alone, could provide a more sensitive framework for identifying emerging risk, guiding recovery, and evaluating the effects of new platforms and countermeasures. Longitudinal psychophysiological monitoring should therefore be considered an enabling research and surveillance capability that may sustain individual pilot health while generating the population-level evidence needed to refine standards and training practices (Saehle, 2025).

## 13 Limitations of this review

This is a narrative rather than a systematic review, reflecting a deliberate choice given the breadth of domains covered and our aim of producing an integrative, policy-relevant synthesis rather than a pooled quantitative estimate of any single effect; as with any narrative synthesis, this choice trades a degree of reproducibility for breadth, and the evidence-selection process, while described as transparently as the format allows in the Literature Search and Synthesis Approach section above, was not conducted as formal independent dual screening with adjudication of disagreements, and no formal risk-of-bias instrument or quantitative synthesis (e.g., meta-analysis) was applied. Readers should treat effect sizes and percentages reported throughout this review as descriptive of individual studies rather than as pooled, precision-weighted estimates.

The underlying evidence base carries its own limitations that a review, however careful, cannot correct for. Publication and selection bias likely favour positive or novel findings over null results, particularly in the fast-moving 2025–2026 literature that makes up a large share of our citations. Samples across the cited studies are predominantly small and predominantly male, reflecting the demographic composition of current fast-jet pilot populations; where sex differences are discussed in this review, this is more often because the underlying literature has identified an evidence gap than because adequately powered sex-stratified data exist, and we have tried to flag this distinction wherever it applies rather than implying equivalent evidence across sexes. Platforms, exposure protocols, and outcome measures are highly heterogeneous across the cited studies, which limits direct comparison and is a further reason we did not attempt quantitative pooling. As university- and government-affiliated researchers without access to classified or export-controlled data, we have relied exclusively on open-literature and unclassified sources; some technical reports, theses, and other grey-literature and non-peer-reviewed sources are included where they were the most direct available evidence (for example, historical anti-G suit development and some early-career conference proceedings), and we have tried to flag these by source type where relevant, but this literature has not undergone the same quality control as peer-reviewed publication. As discussed throughout this review and summarized in the Literature Search and Synthesis Approach section, direct empirical evidence from fifth-generation platforms remains limited relative to the volume of extrapolation from legacy fast-jet aircraft, controlled aerospace analogues, and non-aviation populations, and sixth-generation systems do not yet exist in operational form, so claims about them are necessarily forward-looking inference rather than demonstrated fact; we have tried to signal this distinction explicitly throughout rather than treating all evidentiary tiers as equivalent, but the reader should treat sixth-generation claims in particular with commensurate caution. Coverage is intentionally integrative but not uniform: cardiovascular-autonomic physiology and neurobiomarkers have denser evidence bases, whereas thermal physiology, musculoskeletal loading, sex-specific responses and equipment fit, and countermeasure effectiveness are less systematically characterized; this unevenness reflects both the density of the available literature and the necessarily broad scope of the review.

## 14 Conclusions

The evidence synthesized in this review supports a clear conclusion: the physiological and cognitive demands of next-generation fighter aviation are advancing faster than the human performance science, monitoring frameworks, and protective strategies designed to support the pilots who must meet them. Across the domains examined, acceleration physiology, respiratory life support, cognitive workload, sensory integration, fatigue, neural biomarkers, and psychological health, a consistent pattern emerges: stressors interact and compound rather than operate independently; compensatory mechanisms can preserve apparent performance while physiological reserves are progressively taxed; and current surveillance frameworks were largely designed for an earlier aviation environment, not the increasingly complex demands of fifth- and sixth-generation systems.

As defined in the Introduction, readiness is not a binary clinical state but a dynamic, individualized trajectory shaped by G-force history, respiratory stress, sleep and circadian integrity, cognitive demand, sensory load, allostatic accumulation, and career phase. The cortisol habituation reported in experienced F-35 pilots illustrates a central challenge: conventional biomarkers and self-report may underestimate physiological strain in personnel most likely to be assigned demanding missions. Detecting meaningful deviations in readiness before they manifest as performance decrements is likely to require longitudinal, individualized, multimodal monitoring rather than reliance on episodic, population-normed assessments; advances in wearable biosensors, minimally invasive biochemical monitoring, and AI-enabled analytics make this approach increasingly feasible.

Several evidence gaps warrant explicit prioritization. The near-absence of female pilot data across acceleration physiology, OBOGS research, and biomarker studies remains a significant barrier to health thresholds and monitoring frameworks applicable to an increasingly diverse pilot force. Sixth-generation manned-unmanned teaming will introduce qualitatively new cognitive demands, including supervisory command of autonomous systems, trust calibration under uncertainty, and shared decision authority, for which the psychophysiological evidence base is currently sparse. The ethical governance of continuous biometric monitoring, including consent, data ownership, and prevention of health data being repurposed punitively, must be resolved before operational deployment. These are prerequisites for monitoring systems that are both technically capable and institutionally trusted.

## Glossary

+Gz: Positive acceleration in the head-to-foot axis
A-29: Embraer A-29 Super Tucano aircraft
A-LOC: Almost loss of consciousness
AH-64: Apache attack helicopter
AI: Artificial intelligence
AI/ML: Artificial intelligence/machine learning
ALI: Allostatic load index
ANS: Autonomic nervous system
AQP4: Aquaporin-4
Auto-GCAS: Automatic ground collision avoidance system
BBB: Blood–brain barrier
BChE: Butyrylcholinesterase
BMI: Body mass index
BP: Blood pressure
CFEME: Canadian Forces Environmental Medicine Establishment
CNS: Central nervous system
CO: Cardiac output
CRP: C-reactive protein
DCIEM: Defence and Civil Institute of Environmental Medicine
DHEA(S): Dehydroepiandrosterone and dehydroepiandrosterone sulfate
ECG: Electrocardiography
EDA: Electrodermal activity
EEG: Electroencephalography
EMG: Electromyography
EO/IR: Electro-optical/infrared
F/A-18: F/A-18 Hornet/Super Hornet aircraft
F-15: F-15 Eagle aircraft
F-15I: F-15I Ra’am aircraft
F-16: F-16 Fighting Falcon aircraft
F-22: F-22 Raptor aircraft
F-35: F-35 Lightning II aircraft
F-35A: F-35A conventional take-off and landing variant
F-47: Boeing F-47 sixth-generation fighter aircraft
FEV₁/FVC: Ratio of forced expiratory volume in 1 second to forced vital capacity
FEM: Finite element modelling
fNIRS: Functional near-infrared spectroscopy
GABA: Gamma-aminobutyric acid
GCAP: Global Combat Air Programme
GFAP: Glial fibrillary acidic protein
G-LOC: G-induced loss of consciousness
GSR: Galvanic skin response
HAPP: High-Altitude Pilot Physiological Monitoring Dataset
HFM-274: NATO Human Factors and Medicine Research Task Group 274
HMD: Helmet-mounted display
HMDS: Helmet-mounted display system
HPA: Hypothalamic-pituitary-adrenal
HR: Heart rate
HRF: Heart rate fragmentation
HRV: Heart rate variability
HRVBF: Heart rate variability biofeedback
HUD: Head-up display
ICG: Impedance cardiography
IGF-1: Insulin-like growth factor-1
IL-6: Interleukin-6
IL-10: Interleukin-10
J-20: Chengdu J-20 fighter aircraft
KAAN: Turkish fifth-generation fighter aircraft
LF/HF: Low-frequency/high-frequency ratio
MCP-1: Monocyte chemoattractant protein-1
ML: Machine learning
MRI: Magnetic resonance imaging
MRS: Magnetic resonance spectroscopy
NASA-TLX: National Aeronautics and Space Administration Task Load Index
NATO: North Atlantic Treaty Organization
NF-L: Neurofilament light chain
NFB: Neurofeedback
NGAD: Next Generation Air Dominance
NIRS: Near-infrared spectroscopy
NMR: Nuclear magnetic resonance
NPY: Neuropeptide-Y
NVG: Night vision goggles
OBOGS: On-board oxygen generating system
P3a/P3b: Event-related potential components associated with attentional orienting and stimulus evaluation
PNS: Parasympathetic nervous system
PRDX-6: Peroxiredoxin-6
PVT: Psychomotor vigilance task
RCAF: Royal Canadian Air Force
RMSSD: Root mean square of successive differences
ROM: Range of motion
RR: Interval between successive R waves on the electrocardiogram
rVOR: Vestibulo-ocular reflex gain
SA: Situational awareness
SAM: Sympatho-adrenomedullary
SD: Spatial disorientation
SD1/SD2: Ratio of short- to long-term HRV variability derived from Poincaré
SDNN: Standard deviation of normal-to-normal intervals
SV: Stroke volume
SMR: Sensorimotor rhythm

## References

[B1] Яковенко О. Шинкарук О. Строганов С. (2025). Mechanisms of stress impact on psychophysiological indicators of military personnel and ways for correcting their effects. Науковий часопис Українського державного університету імені Михайла Драгоманова. Серія 15, 243–248. doi: 10.31392/UDU-nc.series15.2025.10(197).46

[B2] Abou-Donia M. B. Abou-Donia M. M. ElMasry E. M. Monro J. A. Mulder M. F. (2013). Autoantibodies to nervous system-specific proteins are elevated in sera of flight crew members: biomarkers for nervous system injury. J. Toxicol. Environ. Health A. 76, 363–380. doi: 10.1080/15287394.2013.765369 23557235

[B3] Abou-Donia M. B. Brahmajothi M. V. (2020). Novel approach for detecting the neurological or behavioral impact of physiological episodes (PEs) in military aircraft crews. Mil. Med. 185, 383–389. doi: 10.1093/milmed/usz295 32074315

[B4] Abou-Donia M. B. Van de Goot F. Mulder M. (2014). Autoantibody markers of neural degeneration are associated with post-mortem histopathological alterations of a neurologically injured pilot. J. Biol. Phys. Chem. 14, 34–53. doi: 10.4024/05ab14a.jbpc.14.03

[B5] Agorastos A. Chrousos G. P. (2022). The neuroendocrinology of stress: the stress-related continuum of chronic disease development. Mol. Psychiatry 27, 502–513. doi: 10.1038/s41380-021-01224-9 34290370

[B6] Ahmed S. (2026). From legitimacy to cognition: the rise of fifth-generation warfare (5GW) 117–146. doi: 10.1007/978-3-032-16585-5_6

[B7] Akiyama M. Takei F. Takada K. (2026). A pilot study of salivary microRNA changes due to high G-load. Aerosp. Med. Hum. Perform. 97, 106–110. doi: 10.3357/amhp.6757.2026 41592718

[B8] Albers F. Maier J. M. Schudlik K. Dupuy J. B. (2025). The effects of wearing pilots' oxygen masks on cognitive performance. Ergonomics 69, 1–11. doi: 10.1080/00140139.2025.2511871 40454790

[B9] Albery W. (2004). Acceleration in other axes affects +Gz tolerance: dynamic centrifuge simulation of agile flight. Aviat. Space Environ. Med. 75, 1–6. 14736126

[B10] Albery W. B. Chelette T. L. (1998). Effect of G suit type on cognitive performance. Aviat. Space Environ. Med. 69, 474–479. 9591617

[B11] Alharasees O. Kale U. (2024). Cognitive load assessment for cadet pilots in simulated aircraft environment-pilot study. Aircraft Eng. Aerospace Technol. 96, 858–872. doi: 10.1108/aeat-12-2023-034435579975

[B12] Alharasees O. Kilikevičius A. Kale U. (2026). “ Real-flight monitoring of vital physiological parameters in pilots: implications for performance, training, and safety”, in: Proceedings of the 4th Cognitive Mobility Conference (Cham: Springer Nature Switzerland AG).

[B13] Ali D. G. Abner E. L. Bahrani A. A. El Khouli R. Gold B. T. Jiang Y. . (2023a). Amyloid-PET and white matter hyperintensities have independent effects on baseline cognitive function and synergistic effects on longitudinal executive function. Brain Sci. 13, 218. doi: 10.3390/brainsci13020218 36831761 PMC9953773

[B14] Ali D. G. Bahrani A. A. El Khouli R. H. Gold B. T. Jiang Y. Zachariou V. . (2023b). White matter hyperintensities influence distal cortical beta-amyloid accumulation in default mode network pathways. Brain Behav. 13, e3209. doi: 10.1002/brb3.3209 37534614 PMC10570488

[B15] Al-Omari A. S. Al-Khalaf H. M. Hussien N. F. M. (2018). Association of flying time with hearing loss in military pilots. Saudi J. Med. Med. Sci. 6, 155–159. doi: 10.4103/sjmms.sjmms_10_18 30787843 PMC6196690

[B16] Aminian N. O. Michael W. Ghoreyshi M. (2025). Cognitive workload assessment: a comprehensive review. J. Aircraft Spacecraft Technol. 9, 12–33. doi: 10.3844/jastsp.2025.12.33

[B17] Arjmand F. Taherkhani S. Shahrezaei A. Sohani M. Esfahani S. S. Marjaei S. . (2025). Neural changes in microgravity: investigating the effects of aerospace environments. IBRO Neurosci. Rep. 19, 973–980. doi: 10.1016/j.ibneur.2025.11.011 41334530 PMC12666590

[B18] Attiah Alzahrani A. Alessa M. F. Musaad Alharbi F. Mohammed Alotaibi T. (2025). Efficacy of fatigue countermeasures on cognitive performance and operational safety in military pilots: a systematic review. Cureus 17, e95823. doi: 10.7759/cureus.95823 41328140 PMC12665280

[B19] Aubert A. McKinley R. (2011). “ Measurements of jet noise aboard us navy aircraft carriers”, in: AIAA Centennial of Naval Aviation Forum" 100 Years of Achievement and Progress (Reston, VA: American Institute of Aeronautics and Astronautics (AIAA)).

[B20] Ayaz H. (2025). Harnessing neuroergonomics: integrating wearable neurotechnologies for enhanced human performance and experience. Neural Interfaces, 145–159. doi: 10.1016/b978-0-443-24824-5.00012-042574925

[B21] Ba̧czalska J. Wojciechowska W. Rojek M. Hahad O. Daiber A. Munzel T. . (2022). Cardiovascular consequences of aircraft noise exposure. Front. Public Health 10, 1058423. doi: 10.3389/fpubh.2022.1058423 PMC9755730 36530719

[B22] Backs R. W. (1995). Going beyond heart rate: autonomic space and cardiovascular assessment of mental workload. Int. J. Aviat. Psychol. 5, 25–48. doi: 10.1207/s15327108ijap0501_3 11541494

[B23] Bagshaw R. J. (1990). The natural history of gravitational induced loss of consciousness. Aviat. Space Environ. Med. 61, 442. 2161649

[B24] Baumgartner H. M. Sigmon J. B. Ciesielski A. L. Lewis R. J. (2025). Changing trends in fatal spatial disorientation accidents in general aviation. J. Saf. Res. 95, 486–494. doi: 10.1016/j.jsr.2025.11.002 41338802

[B25] Beckie T. M. Duffy A. Groer M. W. (2016). The relationship between allostatic load and psychosocial characteristics among women veterans. Women's Health Issues 26, 555–563. doi: 10.1016/j.whi.2016.05.008 27444339

[B26] Belloni G. Curșeu P. L. (2026). Real-time cognitive state monitoring via physiological signals in commercial aviation: a systematic literature review with reasoned snowballing expansion. Safety 12, 56. doi: 10.3390/safety1202005630654563

[B27] Benni P. B. Li J. K. Chen B. Cammarota J. Amory D. W. (2003). Correlation of NIRS determined cerebral oxygenation with severity of pilot +Gz acceleration symptoms. Adv. Exp. Med. Biol. 530, 381–389. doi: 10.1007/978-1-4615-0075-9_35 14562733

[B28] Benson A. J. (2006). Spatial orientation in flight. Ernsting’s Aviation Med. 4, 293–306.

[B29] Bertolini G. Pagnamenta A. Kunz A. Del Torso A. Bron D. (2023). Reduction of the vertical vestibular-ocular reflex in military aircraft pilots exposed to tactical, high-performance flight. Front. Neurol. 14, 949227. doi: 10.3389/fneur.2023.949227 37360332 PMC10289026

[B30] Biondich K. F. (2026). A longitudinal study of performance and workload in military flight training.

[B31] Blacker K. J. Hamilton J. Roush G. Pettijohn K. A. Biggs A. T. (2019). Cognitive training for military application: a review of the literature and practical guide. J. Cognit. Enhancement 3, 30–51. doi: 10.1007/s41465-018-0076-130311153

[B32] Blaginin A. A. Lyashedko S. P. (2023). The problem of spatial disorientation and professional selection of flight personnel. Bull. Russian Military Med. Acad. 25, 357–366. doi: 10.17816/brmma456462

[B33] Bloomfield P. M. Connell C. J. W. Shaw D. M. Fan J. L. Fisher J. P. Gant N. (2026). Systematic review and meta-analysis of acute hypoxia and cognition: domain-specific effects and the role of barometric pressure and carbon dioxide. Neurosci. Biobehav. Rev. 184, 106603. doi: 10.1016/j.neubiorev.2026.106603 41672258

[B34] Bojahr J. Jorres R. A. Kronseder A. Weber F. Ledderhos C. Roiu I. . (2024). Effects of training flights of combat jet pilots on parameters of airway function, diffusing capacity and systemic oxidative stress, and their association with flight parameters. Eur. J. Med. Res. 29, 100. doi: 10.1186/s40001-024-01668-z 38317201 PMC10840181

[B35] Boril J. Smrz V. Pokorny V. Lacinova V. (2025). “ Objective assessment methods for pilots' psychological resistance against loss of spatial orientation”, in: 2025 AIAA DATC/IEEE 44th Digital Avionics Systems Conference (DASC) (Piscataway, NJ: IEEE).

[B36] Bottenheft C. Groen E. L. Mol D. Valk P. J. L. Houben M. M. J. Kingma B. R. M. . (2023). Effects of heat load and hypobaric hypoxia on cognitive performance: a combined stressor approach. Ergonomics 66, 2148–2164. doi: 10.1080/00140139.2023.2190062 36916391

[B37] Britton J. K. Green N. D. Harridge S. D. Peters A. G. Thornton-White C. G. Pollock R. D. (2026). Physiological responses to four different baseline accelerations on a human centrifuge. Aerosp. Med. Hum. Perform. 97, 75–83. doi: 10.3357/amhp.6523.2026 41592724

[B38] Bron D. Kunz A. Grad S. Kincel K. Damato E. Decker M. (2024). Proinflammatory markers and flight hours: preliminary findings. Aerosp. Med. Hum. Perform. 95, 574.

[B39] Bruno J. Sarmento H. Montoro-Bombú R. (2026). G-force tolerance in fighter pilots: a scoping review with evidence gap map. Int. J. Strength Conditioning 6. doi: 10.47206/ijsc.v6i1.574

[B40] Buck J. (2024). Influence of Helmet Mounted Display Symbology and Spatial Audio Horizon Cueing on Prevention and Recovery From Spatial Disorientation (Iowa City, IA: The University of Iowa).

[B41] Buick F. Wood E. Pecaric M. Maloan J. (1995). Methods for Measuring Physiological Responses and Protection in Man Exposed to High+ Gz (Neuilly-sur-Seine: Defence & Civil Institute of Environmental Medicine).

[B42] Burlacu A. Brinza C. Geman O. Karppa M. Hemanth D. J. (2026). Heart rate variability as a dual-use digital biomarker: integrating clinical, AI, and operational perspectives on human performance and resilience. BMC Cardiovasc. Disord. 26, 87. doi: 10.1186/s12872-026-05543-z 41580586 PMC12849089

[B43] Burton R. R. Storm W. F. Johnson L. W. Leverett S. D. Jr. (1977). Stress responses of pilots flying high-performance aircraft during aerial combat maneuvers. Aviat. Space Environ. Med. 48, 301–307. 871290

[B44] Bushby A. Grimshaw T. L. (2025). “ Spatial orientation and disorientation in flight”, in: Ernsting's Aviation and Space Medicine (Boca Raton, FL: CRC Press), 297–332.

[B45] Bustamante-Sanchez A. Tornero-Aguilera J. F. Fernandez-Elias V. E. Hormeno-Holgado A. J. Dalamitros A. A. Clemente-Suarez V. J. (2020). Effect of stress on autonomic and cardiovascular systems in military population: a systematic review. Cardiol. Res. Pract. 2020, 7986249. doi: 10.1155/2020/7986249 32850146 PMC7439199

[B46] Calado A. S. A. Fernandes T. F. R. da Silva H. H. P. de Brito André P. S. (2025). “ Towards psychophysiological monitoring of aircraft pilot crew”, in: Advanced Optical Sensors for Aerospace Applications (Singapore: Springer Nature Singapore), 363–391.

[B47] Caldwell J. A. Knapik J. J. (2025). Trends and factors associated with insomnia and sleep apnea in U.S. military aviators 2006-2022. Aerosp. Med. Hum. Perform. 96, 23–30. doi: 10.3357/amhp.6524.2025 39853290

[B48] Cameron A. (2015). “ In the blink of an eye: head mounted displays development within BAE systems”, in: Display Technologies and Applications for Defense, Security, and Avionics IX; and Head-and Helmet-Mounted Displays XX (Bellingham, WA: SPIE).

[B49] Carroll M. Surpris G. Strally S. Archer M. Hannigan F. Hale K. . (2013). “ Enhancing HMD-based F-35 training through integration of eye tracking and electroencephalography technology”, in: International Conference on Augmented Cognition (Berlin/Heidelberg: Springer).

[B50] Causse M. Mouratille D. Rouillard Y. El Yagoubi R. Matton N. Hidalgo-Munoz A. (2024). How a pilot's brain copes with stress and mental load? Insights from the executive control network. Behav. Brain Res. 456, 114698. doi: 10.2139/ssrn.4392279 37797721

[B51] Chaikovsky I. Senko I. Budnyk M. Matsyshyn V. Ryzhenko T. Budnyk V. . (2026). Analysis of heart rate variability and subtle ECG changes based on machine learning for objective assessment of the psychological state of military personnel. Front. Psychol. 17, 1688230. doi: 10.3389/fpsyg.2026.1688230 41835868 PMC12982173

[B52] Chattopadhyay S. (2025). Sixth generation fighter jets: Latest fighter jet programmes around the world. Vayu Aerospace Defence Rev., 89.

[B53] Chen X. Chu Q. Wang H. Tan X. Duan M. Luo C. (2025b). Flight training and alterations in cortical characteristics. Front. Hum. Neurosci. 19, 1672307. doi: 10.3389/fnhum.2025.1672307 41036167 PMC12479415

[B54] Chen X. Meng Q. Song Q. Xu P. Zhang S. Huang D. . (2025c). Neuroadaptive changes in brain structural–functional coupling among pilots. Front. Neurosci. 19. doi: 10.3389/fnins.2025.1608739 40778354 PMC12329225

[B55] Chen B. Yu Y. Ding L. (2025a). Correlation analysis of physiological parameters of pilot mental workload based on flight simulation. Biomed. Signal Process. Control 109, 108024. doi: 10.1016/j.bspc.2025.10802442574925

[B56] Cheng H. Sun G. Li M. Yin M. Chen H. (2019). Neuron loss and dysfunctionality in hippocampus explain aircraft noise induced working memory impairment: a resting-state fMRI study on military pilots. Biosci. Trends 13, 430–440. doi: 10.5582/bst.2019.01190 31611544

[B57] Clark A. (2024). From Levers to Glass: The Evolution of Aircraft Cockpits and the Future of Aviation Innovation (Tampa, FL: ASXResearch.org).

[B58] Coffey A. Marston P. Smith T. G. Annicelli L. Bilger C. Pollock R. D. . (2026). A proposed framework for the application of physiological monitoring in fast-jet aviation. Aerosp. Med. Hum. Perform. 97, 218–223. doi: 10.3357/amhp.6470.2026 41698666

[B59] Cohen R. Loewenson I. Correl D. (2023). Physiological issues in flight are going down, but still persist. Air Force Times.

[B60] Compierchio A. Illankoon P. Tretten P. (2023). “ Augment-me: an approach for enhancing pilot’s helmet-mounted display visualization for tactical combat effectiveness and survivability,” in Applications of Augmented Reality - Current State of the Art. Ed. Boulanger P. ( IntechOpen, London).

[B61] Compierchio A. Tretten P. Illankoon P. (2024). “ Helmet-mounted display,” in Applications of Augmented Reality-Current State of the Art: Current State of the Art, vol. 3. .

[B62] Connolly D. Davagnanam I. Wylezinska-Arridge M. Mallon D. Wastling S. Lee V. M. (2024a). Brain magnetic resonance imaging responses to nonhypoxic hypobaric decompression. Aerosp. Med. Hum. Perform. 95, 733–740. doi: 10.3357/amhp.6445.2024 39431704

[B63] Connolly D. M. Lee V. M. (2015). Odds ratio meta-analysis and increased prevalence of white matter injury in healthy divers. Aerosp. Med. Hum. Perform. 86, 928–935. doi: 10.3357/amhp.4392.2015 26564757

[B64] Connolly D. M. Lupa H. T. (2021). Prospective study of white matter health for an altitude chamber research program. Aerosp. Med. Hum. Perform. 92, 215–222. doi: 10.3357/amhp.5730.2021 33752784

[B65] Connolly D. M. Madden L. A. Edwards V. C. D'Oyly T. J. Harridge S. D. R. Smith T. G. . (2023). Early human pathophysiological responses to exertional hypobaric decompression stress. Aerosp. Med. Hum. Perform. 94, 738–749. doi: 10.3357/amhp.6247.2023 37726900

[B66] Connolly D. M. Madden L. A. Edwards V. C. Lee V. M. (2024b). Brain and lung biomarker responses to hyperoxic hypobaric decompression. Aerosp. Med. Hum. Perform. 95, 667–674. doi: 10.3357/amhp.6391.2024 39169490

[B67] Corrigan S. L. Roberts S. Warmington S. Drain J. Main L. C. (2021). Monitoring stress and allostatic load in first responders and tactical operators using heart rate variability: a systematic review. BMC Public Health 21, 1701. doi: 10.1186/s12889-021-11595-x 34537038 PMC8449887

[B68] Costa M. D. Davis R. B. Goldberger A. L. (2017). Heart rate fragmentation: A new approach to the analysis of cardiac interbeat interval dynamics. Front. Physiol. 8, 255. doi: 10.3389/fphys.2017.00255 28536533 PMC5422439

[B69] D’Alessandro M. Mackie R. Berger T. Ott C. Sullivan C. Curry I. (2024). Real-time neurophysiological and subjective indices of cognitive engagement in high-speed flight. Aerosp. Med. Hum. Perform. 95, 885–896. doi: 10.3357/AMHP.6489.2024 39711418

[B70] Damato E. G. Boehringer H. E. Ramakrishnan N. Decker M. J. (2026). “ Fatigue in aviators: it is not sleepiness,” in The Scientific Basis of Fatigue. Eds. Martin C. R. Preedy V. R. Patel V. B. Rajendram R. (London: Academic Press), 123–132.

[B71] Damato E. G. Fillioe S. J. Margevicius S. P. Mayes R. S. Somogyi J. E. Vannix I. S. . (2022). Increased serum levels of proinflammatory cytokines are accompanied by fatigue in military T-6A texan II instructor pilots. Front. Physiol. 13, 876750. doi: 10.3389/fphys.2022.876750 35574470 PMC9097024

[B72] Dan A. Reiner M. (2017). Real time EEG based measurements of cognitive load indicates mental states during learning. J. Educ. Data Min. 9, 31–44. doi: 10.5281/zenodo.3554719

[B73] Danielson R. W. Allen C. S. Limardo J. G. Clark J. B. (2019). “ Acoustic and auditory issues,” in Principles of Clinical Medicine for Space Flight. Eds. Barratt M. R. Baker E. S. Pool S. L. ( Springer New York, New York, NY), 175–200.

[B74] Davis J. Stepanak J. Fogarty J. Blue R. (2021). Fundamentals of Aerospace Medicine (Philadelphia, PA: Lippincott Williams & Wilkins).

[B75] De Cesari C. Barravecchia I. Pyankova O. V. Vezza M. Germani M. M. Scebba F. . (2020). “ Hypergravity activates a pro-angiogenic homeostatic response by human capillary endothelial cells,” in International Journal of Molecular Sciences, vol. 21. , 2354. 10.3390/ijms21072354 PMC7177524 32231163

[B76] Department of National Defence (2021). Celebrating Wilbur Rounding Franks, inventor of the G-Suit. The Maple Leaf. (Ottawa, ON: Department of National Defence, Government of Canada). Available online at: https://www.canada.ca/en/department-national-defence/maple-leaf/rcaf/2021/03/celebrating-wilbur-rounding-franks-inventor-of-the-g-suit.html (Accessed September 6, 2026).

[B77] De Luca C. Deeva I. Mariani S. Maiani G. Stancato A. Korkina L. (2009). Monitoring antioxidant defenses and free radical production in space-flight, aviation and railway engine operators, for the prevention and treatment of oxidative stress, immunological impairment, and pre-mature cell aging. Toxicol. Ind. Health 25, 259–267. doi: 10.1177/0748233709103032 19651796

[B78] Deniel J. Dupuy M. Duchevet A. Matton N. Imbert J.-P. Cegarra J. . (2025). Cognitive incapacitation in aviation. Aviation Psychol. Appl. Hum. Factors 15, 62–82. doi: 10.1027/2192-0923/a000286

[B79] Department of National Defence (2023). “ Defence research through the decades,” in Canada.Ca (Ottawa, ON: Department of National Defence, Government of Canada). Available online at: https://www.Canada.ca/en/department-national-defence/campaigns/defence-research-through-the-decades.html.

[B80] de Vries H. J. van der Wal S. J. Delahaij R. Venrooij W. Kamphuis W. (2025). Real-time monitoring of military health and readiness: a perspective on future research. Front. Digit Health 7, 1542140. doi: 10.3389/fdgth.2025.1542140 40400540 PMC12092382

[B81] Dhana A. DeCarli C. Dhana K. Desai P. Wilson R. S. Evans D. A. . (2022). White matter hyperintensity, neurofilament light chain, and cognitive decline. Ann. Clin. Transl. Neurol 10 (3), 321–327. doi: 10.1002/acn3.51720 36542548 PMC10014007

[B82] Dino M. J. Villafuerte C. M. Pimentel J. Sayat J. Vital J. C. Hernandez D. . (2026). Aerospace health: a systematic review and current state of science. Front. Space Technol. 7. doi: 10.3389/frspt.2026.1736627

[B83] Dixon J. B. Clark T. K. Endsley T. C. (2026). Findings from interviews with pilots on spatial disorientation: training, temporal dynamics and countermeasures. Front. Physiol. 16, 1620737. doi: 10.3389/fphys.2025.1620737 41743420 PMC12929113

[B84] Dolev T. Maoz I. Zubedat S. Aga-Mizrachi S. Levkovsky A. Nakdimon I. . (2025). Attention regulation among sleep-deprived air-force pilots. J. Neurosci. Res. 103, e70052. doi: 10.1002/jnr.70052 40491070 PMC12149678

[B85] Dora J. van Hooff M. L. Geurts S. A. Kompier M. A. Bijleveld E. (2022). The effect of opportunity costs on mental fatigue in labor/leisure trade-offs. J. Exp. Psychology: Gen. 151, 695. doi: 10.1037/xge0001095 34472958

[B86] Dussault C. Lely L. Langrume C. Sauvet F. Jouanin J. C. (2009). Heart rate and autonomic balance during stand tests before and after fighter combat missions. Aviat. Space Environ. Med. 80, 796–802. doi: 10.3357/asem.2494.2009 19750876

[B87] Eagan S. M. (2026). “ Ethical analysis of emerging health-monitoring technologies in military settings,” in Artificial Intelligence Ethics in Military Medicine and Humanitarian Healthcare. Eds. Koch B. Winkler D. ( Springer Nature Switzerland, Cham), 81–94. Military and Humanitarian Health Ethics.

[B88] Eiken O. Kolegard R. Bergsten E. Gronkvist M. (2007). G protection: interaction of straining maneuvers and positive pressure breathing. Aviat. Space Environ. Med. 78, 392–398. 17484342

[B89] Elhefnawy N. (2021). A sixth-generation fighter?'An update. An. Update (May 4 2021). doi: 10.2139/ssrn.3839502

[B90] Elliott J. J. Schmitt D. R. (2019). Unexplained physiological episodes. AIR SPACE Power J. 33, 15–32.

[B91] Enriquez-Geppert S. Smit D. de Boer M. C. Daneshnia N. Lafont A. Dehais F. (2024). A systematic narrative review of the involvement of executive functions in flying performance of pilots. Front. Neuroergon. 5, 1462304. doi: 10.3389/fnrgo.2024.1462304 39703619 PMC11656452

[B92] Ercan E. (2021). Effects of aerospace environments on the cardiovascular system. Anatol J. Cardiol. 25, 3–6. doi: 10.5152/anatoljcardiol.2021.s103 34464290 PMC8412052

[B93] Erme E. B. (2026). Comparison of fatigue symptoms according to flight types. Fatigue: Biomedicine Health Behav. 14, 167–186. doi: 10.1080/21641846.2026.262716437339054

[B94] Fan Y. Liang J. Cao X. Pang L. Zhang J. (2022). Effects of noise exposure and mental workload on physiological responses during task execution. Int. J. Environ. Res. Public Health 19, 12434. doi: 10.3390/ijerph191912434 36231736 PMC9566815

[B95] Feigel E. D. Koltun K. J. Lovalekar M. Bird M. B. Kargl C. K. Forse J. N. . (2025a). Identifying a digital phenotype of allostatic load: association between allostatic load index score and wearable physiological response during military training. Am. J. Physiol. Regul. Integr. Comp. Physiol. 329, R946–R958. doi: 10.1152/ajpregu.00216.2025 41223069

[B96] Feigel E. D. Koltun K. J. Lovalekar M. Friedl K. E. Martin B. J. Nindl B. C. (2025b). Advancing the allostatic load model in military training research: from theory to application. Front. Physiol. 16, 1638451. doi: 10.3389/fphys.2025.1638451 41078375 PMC12511115

[B97] Feigel E. D. Koltun K. J. Lovalekar M. Kargl C. K. Bird M. B. Forse J. N. . (2025c). Association of allostatic load measured by allostatic load index on physical performance and psychological responses during arduous military training. Physiol. Rep. 13, e70273. doi: 10.14814/phy2.70273 40110958 PMC11923871

[B98] Flood A. Keegan R. J. (2022). Cognitive resilience to psychological stress in military personnel. Front. Psychol. 13, 809003. doi: 10.3389/fpsyg.2022.809003 35369170 PMC8966027

[B99] Florence G. Riondet L. Serra A. Etienne X. Huart B. van Beers P. . (2005). Psychostimulants and G tolerance in rhesus monkeys: effects of oral modafinil and injected caffeine. Aviat. Space Environ. Med. 76, 121–126. 15742828

[B100] Fox E. L. Cook A. D. Mobley F. Bowers G. Gallagher H. Ennis Z. (2025). Investigating the impacts of cockpit noise exposure on human decision-making. doi: 10.61782/fa.2025.0501

[B101] Fuentes-Garcia J. P. Clemente-Suarez V. J. Marazuela-Martinez M. A. Tornero-Aguilera J. F. Villafaina S. (2021). Impact of real and simulated flights on psychophysiological response of military pilots. Int. J. Environ. Res. Public Health 18, 787. doi: 10.3390/ijerph18020787 33477710 PMC7831919

[B102] Fuentes-Garcia J. P. Leon-Llamas J. L. Villafaina S. (2025). Psychophysiological and dual-task effects of biofeedback and neurofeedback interventions in airforce pilots: a pilot study. Sensors (Basel) 25, 2580. doi: 10.3390/s25082580 40285268 PMC12031066

[B103] Furlong C. E. Richter R. Marsillach J. Zelter A. McDonald M. Rettie A. . (2025). Investigating biomarkers of exposure to jet aircraft oil fumes using mass spectrometry. medRxiv 2025.04.17.25326021. doi: 10.1101/2025.04.17.25326021

[B104] Gaffie D. Guillaume A. Quandieu P. (1993). Modeling and numerical simulation of the cerebral blood flow under acceleration. Physiologist 36, S66–S67. 11538534

[B105] Gee K. L. Neilsen T. B. Wall A. T. Downing J. M. James M. M. (2013). The ‘sound of freedom’: Characterizing jet noise from high-performance military aircraft. Acoust. Today 9, 8–21. doi: 10.1121/1.4821141

[B106] Gertler J. (2020). F-35 joint strike fighter (jsf) program.

[B107] Gil G.-H. Kaber D. Kaufmann K. Kim S.-H. (2012). Effects of modes of cockpit automation on pilot performance and workload in a next generation flight concept of operation. Hum. Factors Ergonomics Manufacturing Service Industries 22, 395–406. doi: 10.1002/hfm.2037742587375

[B108] Gillingham K. K. (1988). High-G stress and orientational stress: physiologic effects of aerial maneuvering. Aviat. Space Environ. Med. 59, A10–A20. 3060092

[B109] Gillingham K. K. (1992). The spatial disorientation problem in the United States Air Force. J. Vestib Res. 2, 297–306. doi: 10.3233/ves-1992-2404 1342404

[B110] Gillingham K. K. Previc F. H. (1993). Spatial orientation in flight.

[B111] Gkintoni E. Halkiopoulos C. (2025). Mapping EEG metrics to human affective and cognitive models: An interdisciplinary scoping review from a cognitive neuroscience perspective. Biomimetics (Basel) 10, 730. doi: 10.3390/biomimetics10110730 41294401 PMC12649996

[B112] Gnerre M. (2026). Beyond the black box: Vocal biomarkers for monitoring stress in pilot-ATC communication.

[B113] Goldstein D. S. McEwen B. (2002). Allostasis, homeostats, and the nature of stress. Stress 5, 55–58. doi: 10.1080/102538902900012345 12171767

[B114] Goswami N. Blaber A. P. Valenti G. Hinghofer-Szalkay H. Evans J. Bailey D. M. . (2026). Gravity, microgravity, and artificial gravity: physiological effects, implementation, and applications. Physiol. Rev. 106, 750–839. doi: 10.1152/physrev.00055.2024 41021767

[B115] Gray G. W. Bost J. W. (1988). Cases from the aerospace medicine residents' teaching file. Case #26. A fighter pilot with a single left frontal lobe infarction and probable mitral valve prolapse and supraventricular arrhythmias. Aviat. Space Environ. Med. 59, 994–996. 3190629

[B116] Greco A. Verde P. De Nuccio C. Spanu C. Minghetti L. Lucertini M. (2025). Hormones and biomarkers in student pilots before and after rehabilitation from airsickness. Aerosp. Med. Hum. Perform. 96, 461–468. doi: 10.3357/amhp.6441.2025 40643388

[B117] Groenendaal W. Lee S. van Hoof C. (2021). Wearable bioimpedance monitoring: Viewpoint for application in chronic conditions. JMIR Biomed. Eng. 6, e22911. doi: 10.2196/22911 38907374 PMC11041432

[B118] Guarnaccia M. Spampinato A. G. Alessi E. Cavallaro S. (2026). Convergent sensing: Integrating biometric and environmental monitoring in next-generation wearables. Biosens.-Basel 16, 43. doi: 10.3390/bios16010043 41590295 PMC12838745

[B119] Guettler N. Holdsworth D. Broekhuizen L. Syburra T. D'Arcy J. Bron D. . (2026). Cardiac rhythm and conduction abnormalities in aircrew. Aerosp. Med. Hum. Perform. 97, 289–295. doi: 10.3357/amhp.6678.2026 41895331

[B120] Guillaume A. Osmont D. Gaffie D. Sarron J. C. Quandieu P. (1997). Effects of perfusion on the mechanical behavior of the brain-exposed to hypergravity. J. Biomech. 30, 383–389. doi: 10.1016/s0021-9290(96)00153-4 9075007

[B121] Guillaume A. I. Osmont D. Gaffie D. Sarron J. C. Quandieu P. (2002). Physiological implications of mechanical effects of +Gz accelerations on brain structures. Aviat. Space Environ. Med. 73, 171–177. 11908880

[B122] Gulgosteren E. Agrali Ermis S. Algin Toros A. Toros T. Serin E. Sekeroglu M. O. . (2025). Sweat, tears, and beyond: advanced wearable sensors for personalized health and athletic performance. Front. Bioeng. Biotechnol. 13, 1684674. doi: 10.3389/fbioe.2025.1684674 41394971 PMC12698657

[B123] Gündüz K. (2025). 5th generation fighter jets and their role in 21st century military strategy 9, 201–206.

[B124] Hadi M. Izzatusholekha I. Dewi M. P. (2026). Implementation of the fatigue risk management system policy for flight safety at the air force regional command I (resources, procedures, and compliance). J. Soc. Sci. (JoSS) 5, 43–57. doi: 10.57185/2yp34g34

[B125] Hagos D. H. Alami H. E. Rawat D. B. (2024). Ai-driven human-autonomy teaming in tactical operations: Proposed framework, challenges, and future directions. arXiv preprint arXiv:2411.09788. doi: 10.48550/arXiv.2411.09788

[B126] Hayano J. Yuda E. (2019). Pitfalls of assessment of autonomic function by heart rate variability. J. Physiol. Anthropol. 38, 3. doi: 10.1186/s40101-019-0193-2 30867063 PMC6416928

[B127] Hegarty-Craver M. Davis-Wilson H. Gaur P. Walls H. Dausch D. Temple D. (2024). “Wearable sensors for service members and first responders: Considerations for using commercially available sensors in continuous monitoring,” ( RTI Press, Research Triangle Park (NC). doi: 10.3768/rtipress.2024.op.0090.2402 38530910

[B128] Hendler E. Hrebien L. Whitley P. (1986). Anti-G suit protection and body position 17.

[B129] Hinckley J. (2024). Enhancing Spatial Disorientation Training in Aviation: Simulator Development and Validation for Future Pilots (Stillwater, OK: Oklahoma State University).

[B130] Ho D. Burns C. (2003). A review of experimentation methodologies in the area of aviation displays and trust in automation.

[B131] Hsu C. C. Lee L. C. Chang H. C. Lai C. Y. Kuo C. L. Chien K. H. (2025). Short-term structural and microvascular ocular changes induced by hypergravity exposure during human centrifuge training. Invest. Ophthalmol. Vis. Sci. 66, 72. doi: 10.1167/iovs.66.11.72 40874695 PMC12400979

[B132] Iacono D. Murphy E. K. Sherman P. M. Chapapas H. Cerqueira B. Christensen C. . (2022). High altitude is associated with pTau deposition, neuroinflammation, and myelin loss. Sci. Rep. 12, 6839. doi: 10.1038/s41598-022-10881-x 35477957 PMC9046305

[B133] Inderberg L. H. Lum H. C. Michael A. (2026). “ Pitch, please! Detecting the onset of spatial disorientation in virtual flight”, in: 2026 IEEE Conference on Cognitive and Computational Aspects of Situation Management (CogSIMA) (Piscataway, NJ: IEEE).

[B134] Izzetoglu K. Richards D. (2019). “ Human performance assessment: evaluation of wearable sensors for monitoring brain activity,” in Improving Aviation Performance Through Applying Engineering Psychology (Boca Raton, FL: CRC Press), 163–180.

[B135] Jenkins J. C. Havig P. R. (2015). “ An evaluation of latency in the F-35 joint strike fighter helmet mounted display”, in: Proceedings of the Human Factors and Ergonomics Society Annual Meeting (Thousand Oaks, CA: SAGE Publications).

[B136] Jia C. Yang M. Zhao C. (2026). High-altitude pilot physiological monitoring dataset: Respiratory performance and SpO_2_ analysis. Sci. Data 13, 192. doi: 10.1038/s41597-025-06508-1 41588022 PMC12886808

[B137] Johnson B. K. (2024). “ Physiology of the autonomic nervous system,” in Basic Sciences in Anesthesia. Eds. Argalious M. Farag E. Sharma D. ( Springer Nature Switzerland, Cham), 377–386.

[B138] Johnston J. Radhakrishnan S. Shamwell J. (2026). “ Predicting cognitive state and workload of aviators with machine learning of physiological data”, in: 2026 IEEE Aerospace Conference (Piscataway, NJ: IEEE), 7–14.

[B139] Jones M.-L. Britton J. K. Green N. D. C. Pollock R. D. (2026). G-induced alterations of consciousness in UK military aviation. Aerosp. Med. Hum. Perform. 97, 257–263. doi: 10.3357/amhp.6452.2026 41895324

[B140] Juster R. P. McEwen B. S. Lupien S. J. (2010). Allostatic load biomarkers of chronic stress and impact on health and cognition. Neurosci. Biobehav. Rev. 35, 2–16. doi: 10.1016/j.neubiorev.2009.10.002 19822172

[B141] Kapela A. (2026). Human Factors for Pilots and Aviation Specialists: Flight Safety Through Modern Approaches to Human Behavior (Boca Raton, FL: CRC Press).

[B142] Karatsoreos I. N. McEwen B. S. (2011). Psychobiological allostasis: resistance, resilience and vulnerability. Trends Cognit. Sci. 15, 576–584. doi: 10.1016/j.tics.2011.10.005 22078931

[B143] Keijsper G. (2024). F-35 in service: With air forces around the world.

[B144] Khalaj B. K. Abdessalem H. B. Frasson C. (2026). “ Impact of experience on cognitive load and physiological responses in aviation pilots,” in: Generative Systems and Intelligent Tutoring Systems: 21st International Conference, ITS 2025, Alexandroupolis, Greece, June 2-6, 2025, Proceedings, Part II. ( Springer Nature Switzerland, Cham). doi: 10.1007/978-3-031-63031-6_6

[B145] Kim S. H. Park T. W. Cho S. Yang T. Yoo S. Ilya K. . (2026). Wireless, skin-interfaced multimodal sensing system for continuous psychophysiological monitoring-a wearable polygraph device. Sci. Adv. 12, eaed3162. doi: 10.1126/sciadv.aed3162 42127197 PMC13170674

[B146] Kincel K. Jr. Damato E. Decker M. Grad S. Kunz A. Bron D. (2024). Monocyte chemoattractant protein-1 is increased in Swiss military aviators with back and neck pain. Aerosp. Med. Hum. Perform. 95, 614.

[B147] Koltun K. J. Bird M. B. Forse J. N. Nindl B. C. (2023). Physiological biomarker monitoring during arduous military training: Maintaining readiness and performance. J. Sci. Med. Sport 26 Suppl 1, S64–S70. doi: 10.1016/j.jsams.2022.12.005 36631385

[B148] Konings M. Wiekenkamp A. C. Wingelaar-Jagt Y. Q. Wingelaar T. T. (2025). Evaluating the impact of flight hours on pulmonary function in military pilots. Aerosp. Med. Hum. Perform. 96, 1079–1083. doi: 10.3357/amhp.6753.2025 41360150

[B149] Korentsides J. Keebler J. R. Fausett C. M. Patel S. M. Lazzara E. H. (2024). Human-AI teams in aviation: Considerations from human factors and team science. J. Aviation/Aerospace Educ. Res. 33, 7. doi: 10.58940/2329-258x.2046

[B150] Koskelo J. Lehmusaho A. Laitinen T. P. Hartikainen J. E. K. Lahtinen T. M. M. Leino T. K. . (2024). Cardiac autonomic responses in relation to cognitive workload during simulated military flight. Appl. Ergon. 121, 104370. doi: 10.1016/j.apergo.2024.104370 39186837

[B151] Kula Y. Horosov R. Gidron Y. Ekshtein A. Gordon B. Iversen Z. . (2025a). Pre-training heart rate variability as a predictor of Air Force Academy completion. PloS One 20, e0327406. doi: 10.1371/journal.pone.0327406 40773446 PMC12331047

[B152] Kula Y. Iversen Z. Cohen A. Levine A. D. Gidron Y. (2025b). Does vagal nerve activity predict performance in a naval commando selection test? Appl. Psychophysiol. Biofeedback 50, 349–357. doi: 10.1007/s10484-025-09702-4 40111586 PMC12331781

[B153] Kuo C. Y. Hung C. L. Chen H. C. Shih C. P. Lu R. H. Chen C. W. . (2021). The immediate and long-term impact of military aircraft noise on hearing: A cross-sectional comparison of fighter pilots and ground staff. Int. J. Environ. Res. Public Health 18, 2982. doi: 10.3390/ijerph18062982 33799421 PMC7999744

[B154] Kurihara K. Kikukawa A. Kobayashi A. Nakadate T. (2007). Frontal cortical oxygenation changes during gravity-induced loss of consciousness in humans: A near-infrared spatially resolved spectroscopic study. J. Appl. Physiol. (1985) 103, 1326–1331. doi: 10.1152/japplphysiol.01191.2006 17641211

[B155] Laborde S. Mosley E. Thayer J. F. (2017). Heart rate variability and cardiac vagal tone in psychophysiological research - Recommendations for experiment planning, data analysis, and data reporting. Front. Psychol. 8, 213. doi: 10.3389/fpsyg.2017.00213 28265249 PMC5316555

[B156] Lalande S. Buick F. (2009). Physiologic +G_z_ tolerance responses over successive +G_z_ exposures in simulated air combat maneuvers. Aviat. Space Environ. Med. 80, 1032–1038. doi: 10.3357/ASEM.2525.2009 20027850

[B157] Lan X. Zhu W. Du J. Wang J. Yang M. Xu Y. . (2023). High G tolerance and cardiopulmonary reserve function in healthy air force aviators. Aerosp. Med. Hum. Perform. 94, 911–916. doi: 10.3357/amhp.6297.2023 38176048

[B158] Langridge D. Cable G. (2025). Surviving space: A review of the medical considerations of acceleration force exposure in commercial suborbital spaceflight. J. Australas. Soc. Aerospace Med. 14, 31–40. doi: 10.2478/asam-2025-0004

[B159] Ledderhos C. Kuhn S. Sonksen S. E. Gens A. Weber F. (2025). The impact of occupational nonhypoxic hypobaria on cerebral function and integrity. Sci. Rep. 15, 29777. doi: 10.1038/s41598-025-13622-y 40804070 PMC12350639

[B160] LeDuc P. A. Greig J. L. Dumond S. L. (2005). Involuntary eye responses as measures of fatigue in US Army Apache aviators. Aviat. Space Environ. Med. 76, C86–C91. 16018334

[B161] Lee S. Kim J. H. Kim H. Kim S. H. Park S. S. Hong C. W. . (2025b). Investigating the effect of mindfulness training for stress management in military training: The relationship between the autonomic nervous system and emotional regulation. BMC Psychol. 13, 13. doi: 10.1186/s40359-024-02322-3 39773484 PMC11706002

[B162] Lee M. J. Yoon S. Kim I. K. Sung J. Y. (2025a). Effects of gravitational acceleration on physical fitness, stress, and immunity levels of prospective air force pilots. Stress Health 41, e70060. doi: 10.1002/smi.70060 40497823

[B163] Leinonen A. M. Varis N. O. Kokki H. J. Leino T. K. (2022). A new method for combined hyperventilation and hypoxia training in a tactical fighter simulator. Aerosp. Med. Hum. Perform. 93, 681–687. doi: 10.3357/amhp.6027.2022 36224739

[B164] Lejeune N. (2026). Stress in the cockpit: How pilots make decisions under pressure. Intellectual Arch. 14, 25–41. doi: 10.32370/ia_2025_04_4

[B165] Lengersdorf A. Post J. Guettler N. Sammito S. (2025). Pulmonary function changes in fighter pilots with positive pressure ventilation. Healthcare (Basel) 13, 2020. doi: 10.3390/healthcare13162020 40868636 PMC12385563

[B166] Levin B. Andersson J. Karlsson T. (2007). Memory performance during G exposure as assessed by a word recognition task. Aviat. Space Environ. Med. 78, 587–592. 17571659

[B167] Lewkowicz R. Stasiak K. Zieliński P. (2026). Predictive validity of selected cognitive tests for the assessment of pilot flight performance in night vision goggles. Cognition Technol. Work., 1–13. doi: 10.1007/s10111-026-00859-430311153

[B168] Li Y. Li K. Wang S. Chen X. Wen D. (2022). Pilot behavior recognition based on multi-modality fusion technology using physiological characteristics. Biosens.-Basel 12. doi: 10.3390/bios12060404 35735552 PMC9221330

[B169] Lindsay K. Van den Top E. (2025). The utility of heart rate variability in aviation and space medicine. J. Australas. Soc. Aerospace Med. 14, 19–26. doi: 10.2478/asam-2025-0002

[B170] Little A. L. (2025). The A52 breath method: A narrative review of breathwork for mental health and stress resilience. Stress Health 41, e70098. doi: 10.1002/smi.70098 40792649 PMC12341363

[B171] Liu T. Mao Y. Dou H. Zhang W. Yang J. Wu P. . (2025). Emerging wearable acoustic sensing technologies. Adv. Sci. (Weinh) 12, e2408653. doi: 10.1002/advs.202408653 39749384 PMC11809411

[B172] Li Volsi G. Monte I. P. Aruta A. Gulizzi A. Libra A. Mirulla S. . (2023). Heart rate variability indices of student pilots undergo modifications during flight training. Aerosp. Med. Hum. Perform. 94, 835–842. doi: 10.3357/amhp.6305.2023 37853596

[B173] Lytle J. A. Kittelson A. J. Cardinale T. Kingsbury T. Infante E. Winters J. D. . (2026). A comparative study of vestibular performance in pilot and non-pilot service members in naval aviation. Mil. Med., usaf604. doi: 10.1093/milmed/usaf604 41617204

[B174] Mędrzycka J. Rotuski G. Różycki R. Gołębiewska J. (2025). Role of flight duration and altitude in ocular health of military pilots. Med. Sci. Monitor: Int. Med. J. Exp. Clin. Res. 31, e948936. doi: 10.12659/MSM.948936 PMC12396089 40849679

[B175] Ma H. He Y. Ma Y. Han G. Zhang Z. Tian B. (2025). Smart polymers and adaptive systems in pilot suit engineering: Toward autonomous, responsive, and wearable flight technologies. Nanomaterials (Basel) 15, 1228. doi: 10.3390/nano15161228 40863808 PMC12388872

[B176] Maculewicz E. Pabin A. Dziuda L. Bialek M. Bialek A. (2023). Selected exogenous (occupational and environmental) risk factors for cardiovascular diseases in military and aviation. J. Clin. Med. 12, 7492. doi: 10.3390/jcm12237492 38068543 PMC10706953

[B177] Mago V. (2025). “ Decompression sickness,” in Hyperbaric Medicine ( Springer Nature Singapore, Singapore), 133–141.

[B178] Maier M. Powell D. Murchie P. Allan J. L. (2025). Systematic review of the effects of decision fatigue in healthcare professionals on medical decision-making. Health Psychol. Rev. 19, 717–762. doi: 10.1080/17437199.2025.2513916 40591577

[B179] Mansikka H. Simola P. Virtanen K. Harris D. Oksama L. (2016). Fighter pilots' heart rate, heart rate variation and performance during instrument approaches. Ergonomics 59, 1344–1352. doi: 10.1080/00140139.2015.1136699 26942339

[B180] Marotte H. (2006). The exposure of man to altitude when flying: From Paul Bert to today. J. Soc Biol. 200, 251–255. doi: 10.1051/jbio:2006029 17417140

[B181] Marqueze E. C. de Sa E. B. E. A. Russo A. C. Furst M. S. G. Roscani R. C. Guimaraes P. C. V. . (2023). Organizational risk factors for aircrew health: A systematic review of observational studies. Int. J. Environ. Res. Public Health 20, 3401. doi: 10.3390/ijerph20043401 36834104 PMC9960399

[B182] Martins A. P. (2016). A review of important cognitive concepts in aviation. Aviation 20, 65–84. doi: 10.3846/16487788.2016.1196559

[B183] Masi G. Amprimo G. Ferraris C. Priano L. (2023). Stress and workload assessment in aviation-a narrative review. Sensors (Basel) 23, 3556. doi: 10.3390/s23073556 37050616 PMC10098909

[B184] Mauss D. Li J. Schmidt B. Angerer P. Jarczok M. N. (2015). Measuring allostatic load in the workforce: A systematic review. Ind. Health 53, 5–20. doi: 10.2486/indhealth.2014-0122 25224337 PMC4331190

[B185] Mayes R. (2024). Physiologic episodes in high-performance aviation: NATO working group findings and next steps. Aerosp. Med. Hum. Perform. 95, 591.

[B186] McCrory C. McLoughlin S. Layte R. NiCheallaigh C. O'Halloran A. M. Barros H. . (2023). Towards a consensus definition of allostatic load: A multi-cohort, multi-system, multi-biomarker individual participant data (IPD) meta-analysis. Psychoneuroendocrinology 153, 106117. doi: 10.1016/j.psyneuen.2023.106117 37100008 PMC10620736

[B187] McEwen B. S. (1998). Protective and damaging effects of stress mediators. N. Engl. J. Med. 338, 171–179. doi: 10.7551/mitpress/3077.003.0079 9428819

[B188] McEwen B. S. (2006). Protective and damaging effects of stress mediators: Central role of the brain. Dialogues Clin. Neurosci. 8, 367–381. doi: 10.1016/s0079-6123(08)62128-7 17290796 PMC3181832

[B189] McEwen B. S. (2017). Neurobiological and systemic effects of chronic stress. Chronic Stress (Thousand Oaks) 1, 2470547017692328. doi: 10.1177/2470547017692328 28856337 PMC5573220

[B190] McEwen B. S. Karatsoreos I. N. (2015). Sleep deprivation and circadian disruption: Stress, allostasis, and allostatic load. Sleep Med. Clin. 10, 1–10. doi: 10.1016/j.jsmc.2022.03.005 26055668 PMC8935364

[B191] McGuire S. A. Boone G. R. Sherman P. M. Tate D. F. Wood J. D. Patel B. . (2016). White matter integrity in high-altitude pilots exposed to hypobaria. Aerosp. Med. Hum. Perform. 87, 983–988. doi: 10.3357/amhp.4585.2016 28323582 PMC5455142

[B192] McGuire S. Sherman P. Profenna L. Grogan P. Sladky J. Brown A. . (2013). White matter hyperintensities on MRI in high-altitude U-2 pilots. Neurology 81, 729–735. doi: 10.1212/wnl.0b013e3182a1ab12 23960192 PMC3776459

[B193] McGuire S. A. Tate D. F. Wood J. Sladky J. H. McDonald K. Sherman P. M. . (2014). Lower neurocognitive function in U-2 pilots: Relationship to white matter hyperintensities. Neurology 83, 638–645. doi: 10.1212/wnl.0000000000000694 25008397 PMC4141992

[B194] Meeks R. K. Anderson J. Bell P. M. (2018). Physiology of spatial orientation. 30085518

[B195] Miller C. (2025). Aerial Combatpublifye as (Oslo: Publifye AS).

[B196] Mills P. Drew J. G. Costello R. Cochran S. Grill B. Kepe M. . (2026). Improving Interoperability With Allies and Partners: Aircraft Maintenance and Base Operating Support (Santa Monica, CA: RAND Corporation).

[B197] Mohamed M. I. Dean Y. E. Rath S. Rehman A. U. Aleem M. A. Hamza A. . (2026). Morphological brain changes in high-altitude careers and spaceflights: A systematic review and meta-analysis. BMC Neurol 26 (1), 356. doi: 10.1186/s12883-025-04272-8 42186000 PMC13220517

[B198] Molitor M. Bayo-Jimenez M. T. Hahad O. Witzler C. Finger S. Garlapati V. S. . (2023). Aircraft noise exposure induces pro-inflammatory vascular conditioning and amplifies vascular dysfunction and impairment of cardiac function after myocardial infarction. Cardiovasc. Res. 119, 1416–1426. doi: 10.1093/cvr/cvad021 36702626 PMC10262193

[B199] Molloy O. Eves G. Vahidnia S. Shahin M. (2026). Machine learning methods for cognitive load analysis and classification in aviation: A systematic review. Int. J. Hum.-Comput. Interact., 1–28. doi: 10.1080/10447318.2026.263215137339054

[B200] Moore D. Carrothers B. Rolf Z. De Reus A. Van Den Berg T. Bigmore D. . (2023). Flight testing of helmet mounted displays.

[B201] Morfitt G. E. (2022). Augmenting Heads-Up Displays With Intelligent Agents: A Human Factors Approach (Glassboro, NJ: Rowan University).

[B202] Morgan-Warren P. Woodcock M. (2014). Visual aspects of advanced sensors and helmet-mounted displays. Aeronaut. J. 118, 155–168. doi: 10.1017/s000192400000904041292463

[B203] Mucci N. Traversini V. Lulli L. G. Vimercati L. Rapisarda V. Galea R. P. . (2021). Neurobehavioral alterations in occupational noise exposure: A systematic review. Sustainability 13, 12224. doi: 10.20944/preprints202103.0740.v1

[B204] Muhr P. Johnson A. C. Selander J. Svensson E. Rosenhall U. (2019). Noise exposure and hearing impairment in air force pilots. Aerosp. Med. Hum. Perform. 90, 757–763. doi: 10.3357/amhp.5353.2019 31426890

[B205] Munroe H. E. (1919). Observations on Flying Sickness, with special Reference to its Diagnosis. Can. Med. Assoc. J. 9, 883–895. PMC1523605 20311321

[B206] Nakdimon I. Algranati D. Glass A. Levkovsky A. Eliyahu U. Ben-Ari O. . (2025). Individual factors affecting oxygen saturation under acute hypoxia exposure in air personnel. Respir. Med. 244, 108175. doi: 10.1016/j.rmed.2025.108175 40414316

[B207] Nakdimon I. Gordon B. (2025). Spatial disorientation event during flight due to proposed reverse-dip visual illusion. Aerosp. Med. Hum. Perform. 96, 1015–1018. doi: 10.3357/amhp.6656.2025 41253365

[B208] Nesci A. Scagliusi A. Ruggieri V. Ponziani F. R. Parati E. A. Trivelloni P. . (2026). Altered endothelial function in high-performance military fighter pilots. Am. J. Physiol. Heart Circ. Physiol. 330, H1368–H1374. doi: 10.1152/ajpheart.00541.2025 41855027

[B209] Newman D. G. (2017). Flying Fast Jets: Human Factors and Performance Limitations. 1st ed. (Place of publication not identified: CRC Press).

[B210] Newman D. G. Callister R. (1999). The non-invasive assessment of stroke volume and cardiac output by impedance cardiography: a review. Aviat. Space Environ. Med. 70, 780–789. 10447052

[B211] Norman G. J. Morgan E. Raja S. Berntson G. G. (2026). Wearable ANS monitoring in real life: A critical review of context-sensitive interpretation and implications for psychophysiology. Auton. Neurosci. 263, 103364. doi: 10.1016/j.autneu.2025.103364 41506124

[B212] Okere E. E. Balyan V. (2025). Sixth generation enabling technologies and machine learning intersection: A performance optimization perspective. Future Internet 17, 50. doi: 10.3390/fi1702005030654563

[B213] Osborn K. (2021). Sensor fusion: the secret sauce that makes the F-35 revolutionary. Natl. Interest.

[B214] Otsuka Y. Onozawa A. Kikukawa A. Miyamoto Y. (2007). Effects of flight workload on urinary catecholamine responses in experienced military pilots. Percept. Mot Skills 105, 563–571. doi: 10.2466/pms.105.6.563-571 18065080

[B215] Otsuka Y. Onozawa A. Miyamoto Y. (2006). Hormonal responses of pilots to training flights: the effects of experience on apparent stress. Aviat. Space Environ. Med. 77, 410–414. 16676652

[B216] Oubaid V. (2023). “ Psychological stress and the autonomic nervous system,” in Primer on the Autonomic Nervous System. Eds. Biaggioni I. Browning K. Fink G. Jordan J. Low P. A. Paton J. F. R. (London: Academic Press), 301–304.

[B217] Ozdemir U. Savas T. (2026). Development of a simulator system enabling flight data recording and post-flight analysis for trainee pilots: A proof of concept. Aerospace 13, 149. doi: 10.3390/aerospace1302014930654563

[B218] Paillard A. C. Quarck G. Denise P. (2014). Sensorial countermeasures for vestibular spatial disorientation. Aviat. Space Environ. Med. 85, 563–567. doi: 10.3357/asem.3735.2014 24834571

[B219] Paul T. S. Durrmeyer C. Bliss J. P. Ferrari V. (2026). Adaptive autonomy in the air force: testbed for human-al collaboration. Hum. Interaction Emerging Technol. (IHIET-AI 2026): Artif. Intell. Future Appl. 201, 213. doi: 10.54941/ahfe1007175

[B220] Pennings H. J. Oprins E. A. Wittenberg H. Houben M. M. Groen E. L. (2020). Spatial disorientation survey among military pilots. Aerosp. Med. Hum. Perform. 91, 4–10. doi: 10.3357/amhp.5446.2020 31852567

[B221] Pessiglione M. Blain B. Wiehler A. Naik S. (2025). Origins and consequences of cognitive fatigue. Trends Cognit. Sci. 29, 730–749. doi: 10.1016/j.tics.2025.02.005 40169294

[B222] Pfaltz M. C. Schnyder U. (2023). Allostatic load and allostatic overload: preventive and clinical implications. Psychother. Psychosom 92, 279–282. doi: 10.1159/000534340 37931612 PMC10716872

[B223] Pignatiello G. A. Martin R. J. Hickman R. L. Jr. (2020). Decision fatigue: A conceptual analysis. J. Health Psychol. 25, 123–135. doi: 10.1177/1359105318763510 29569950 PMC6119549

[B224] Poisson R. J. Miller M. E. (2014). Spatial disorientation mishap trends in the U.S. Air force 1993-2013. Aviat. Space Environ. Med. 85, 919–924. doi: 10.3357/asem.3971.2014 25197890

[B225] Pongsakornsathien N. Gardi A. Lim Y. Sabatini R. Kistan T. (2022). Wearable cardiorespiratory sensors for aerospace applications. Sensors (Basel) 22, 4673. doi: 10.3390/s22134673 35808167 PMC9268781

[B226] Pontiggia A. Quiquempoix M. Fabries P. Beauchamps V. Jacques C. Guillard M. . (2026). Robust multimodal mental workload classification: A cross-physiological condition machine learning approach. Comput. Methods Programs Biomed. 277, 109251. doi: 10.1016/j.cmpb.2026.109251 41576780

[B227] Post T. E. Denney C. Cohen A. Jordan J. Limper U. (2026). Human hypoxia models in aerospace medicine: Potential applications for human pharmacological research. Br. J. Clin. Pharmacol. 92, 58–72. doi: 10.1111/bcp.16040 38556349 PMC12746364

[B228] Powell F. L. Garcia N. (2000). Physiological effects of intermittent hypoxia. High Alt. Med. Biol. 1, 125–136. doi: 10.1089/15270290050074279 11256564

[B229] Raff H. Hainsworth K. R. Cutlan R. Sherman K. A. Stemper B. D. (2025). Flight-associated cortisol dynamics and subjective assessments of pain and stress in fighter pilots: an exploratory study. Eur. J. Appl. Physiol. 125, 3385–3395. doi: 10.1007/s00421-025-05837-8 40512180

[B230] Rajguru R. (2013). Military aircrew and noise-induced hearing loss: prevention and management. Aviat. Space Environ. Med. 84, 1268–1276. doi: 10.3357/asem.3503.2013 24459798

[B231] Ramirez J. Marraffino V. N. Vartanian O. Rhind S. G. Battista A. P. D. King K. . (2025). Correlates of white matter hyperintensities in Royal Canadian Air Force pilots and aircrew. Alzheimer's Dementia 21, e099047. doi: 10.1002/alz70857_09904742587375

[B232] Reale C. Salwei M. E. Militello L. G. Weinger M. B. Burden A. Sushereba C. . (2023). Decision-making during high-risk events: A systematic literature review. J. Cognit. Eng. Decis. Mak 17, 188–212. doi: 10.1177/15553434221147415 37823061 PMC10564111

[B233] Reuten A. J. C. Bruin J. Ledegang W. D. Groen E. L. Houben M. M. J. Cornelisse E.-J. . (2026). Physiological and subjective assessment of fighter pilot mental workload. Aerosp. Med. Hum. Perform. 97, 592–600. doi: 10.3357/AMHP.6875.2026 42542328

[B234] Rhind S. G. Shiu M. Y. Vartanian O. Allen S. Palmer M. Ramirez J. . (2024). Neurological biomarker profiles in royal Canadian air force (RCAF) pilots and aircrew. Brain Sci. 14, 1296. doi: 10.3390/brainsci14121296 39766495 PMC11674576

[B235] Rice G. M. Linnville S. Luse T. M. Erpenbach J. D. Larocco S. A. (2026). Prevalence of neurological and mental health illness in U.S. Naval aviators: A retrospective cohort study. Aerosp. Med. Hum. Perform. 97, 264–271. doi: 10.3357/amhp.6806.2026 41895329

[B236] Richardson W. (2019). Leveraging capability: A study of the interoperability of fourth-and fifth-generation NATO fighter aircraft. J. Military Strategic Stud. 19, 126–147. doi: 10.55016/newnpg93

[B237] Robinson A. Silva L. Linder J. (2026). Better, faster, smarter: scalable aircrew training for modern air power. Contemp. Issues Air Space Power 4, BP55099219. doi: 10.58930/bp55099219

[B238] Roman N. (2021). Psychophysiological readiness of tactical aviation pilots as a subject of multidisciplinary research. Int. J. Psychiatry Res. 4, e1111-e1111. doi: 10.33425/2641-4317.1111

[B239] Rood G. M. James S. H. (2024). “ In-flight noise and communications,” in Ernsting's Aviation and Space Medicine, 6th ed. Eds. Gradwell D. P. Wilkinson E. S. (Boca Raton, FL: CRC Press), 754–773.

[B240] Rosa E. Eiken O. Grönkvist M. Kölegård R. Dahlström N. Knez I. . (2020). Effects of fatigue on cognitive performance in long-duration simulated flight missions. Aviation Psychol. Appl. Hum. Factors 10 (2), 82–93. doi: 10.1027/2192-0923/a000191

[B241] Rosa E. Lyskov E. Gronkvist M. Kolegard R. Dahlstrom N. Knez I. . (2022). Cognitive performance, fatigue, emotional, and physiological strains in simulated long-duration flight missions. Mil. Psychol. 34, 224–236. doi: 10.1080/08995605.2021.1989236 38536372 PMC10013546

[B242] Rosales A. M. Shute R. J. Hailes W. S. Collins C. W. Ruby B. C. Slivka D. R. (2022). Independent effects of acute normobaric hypoxia and hypobaric hypoxia on human physiology. Sci. Rep. 12, 19570. doi: 10.1038/s41598-022-23698-5 36379983 PMC9666440

[B243] Rupert A. H. McGrath B. Mortimer B. J. Brill J. C. (2023). “ Spatial orientation modeling: position-motion paradox in hypergravity”, in: 2023 IEEE Aerospace Conference (Piscataway, NJ: IEEE).

[B244] Şengöz M. (2026). “ The transformation and future perspectives of modernized and next-generation fighter aircrafts,” in Industrial Policy, Innovation, and Complexity: Comparative Analysis, Trends, and Prospects (Hershey, PA: IGI Global Scientific Publishing), 263–302.

[B245] Saehle T. (2025). Evidence-based aeromedical assessments. Aerosp. Med. Hum. Perform. 96, 71–77. doi: 10.3357/amhp.6540.2024 39853285

[B246] Sale C. (2025). Detecting Cognitive Load Through the Structure of Behaviour: An Ecological-Dynamical Approach (Sydney, NSW: Macquarie University).

[B247] Sandor P. M. Pellieux L. Godfroy M. Ossard G. Dancer A. (2004). Hearing thresholds during Gz acceleration with masking noise. Aviat. Space Environ. Med. 75, 952–955. 15558994

[B248] Schmidt R. (2026). Decision fatigue in military operations: cognitive challenges, performance impacts, and mitigation strategies. Perform. Impacts Mitigation Strategies (March 06 2026). doi: 10.2139/ssrn.6359880

[B249] Schnell T. Reichlen C. Reuter C. Geiselman E. Knox J. Williams H. (2017). “ A comparison of helmet-mounted display symbologies during live flight operational tasks”, in: 19th International Symposium on Aviation Psychology (Dayton, OH: Wright State University).

[B250] Sebastian S. A. Sethi Y. Johal G. (2025). Association between long-term aircraft noise exposure and cardiovascular disease: a meta-analysis with focused subgroup analyses. Eur. J. Prev. Cardiol. 32, 1943–1959. doi: 10.1093/eurjpc/zwaf471 40738497

[B251] Seeman T. E. McEwen B. S. Rowe J. W. Singer B. H. (2001). Allostatic load as a marker of cumulative biological risk: MacArthur studies of successful aging. Proc. Natl. Acad. Sci. U.S.A. 98, 4770–4775. doi: 10.1073/pnas.081072698 11287659 PMC31909

[B252] Seidler R. D. Mao X. W. Tays G. D. Wang T. Zu Eulenburg P. (2024). Effects of spaceflight on the brain. Lancet Neurol. 23, 826–835. doi: 10.1016/s1474-4422(24)00224-2 38945144

[B253] Sekel N. M. Beckner M. E. Conkright W. R. LaGoy A. D. Proessl F. Lovalekar M. . (2023). Military tactical adaptive decision making during simulated military operational stress is influenced by personality, resilience, aerobic fitness, and neurocognitive function. Front. Psychol. 14, 1102425. doi: 10.3389/fpsyg.2023.1102425 36844343 PMC9944034

[B254] Shang L. Lu M. Qian C. Wanyan X. Zhou Y. Liu S. (2025). Research on the evaluation model of pilots' in-flight stress state based on flight critical events. Front. Physiol. 16, 1713035. doi: 10.3389/fphys.2025.1713035 41789155 PMC12956700

[B255] Shaw D. M. Cabre G. Gant N. (2021). Hypoxic hypoxia and brain function in military aviation: Basic physiology and applied perspectives. Front. Physiol. 12, 665821. doi: 10.3389/fphys.2021.665821 34093227 PMC8171399

[B256] Shaw D. M. Harrell J. W. (2023). Integrating physiological monitoring systems in military aviation: a brief narrative review of its importance, opportunities, and risks. Ergonomics 66, 2242–2254. doi: 10.1080/00140139.2023.2194592 36946542

[B257] Sheel A. W. (2023). Intermittent hypoxia and respiratory plasticity: The good, the bad, and the unknown. Funct. (Oxf) 4, zqad045. doi: 10.1093/function/zqad045 37753181 PMC10519271

[B258] Shen Y. Bateer He Q. Cheng L. Fu Q. (2026). Evaluating civil aircraft crew go-around performance oriented towards airworthiness verification. Sci. Rep. 16, 4510. doi: 10.1038/s41598-025-34698-6 41501322 PMC12864958

[B259] Shen H. Hao M. Ren J. Chen K. Gao Y. (2025). Experimental study on the effects of cockpit noise on physiological indicators of pilots. Sensors (Basel) 25, 4175. doi: 10.3390/s25134175 40648430 PMC12251948

[B260] Sherman P. (2024). Warfighter brain health in hypobaric environments post NATO HFM RTG-274. Aerosp. Med. Hum. Perform. 95, 608.

[B261] Sherwood A. Allen M. T. Fahrenberg J. Kelsey R. M. Lovallo W. R. van Doornen L. J. (1990). Methodological guidelines for impedance cardiography. Psychophysiology 27, 1–23. doi: 10.1111/j.1469-8986.1990.tb02171.x 2187214

[B262] Shukla M. Patel J. C. Mani K. V. Nayak D. Shukla M. Sinha D. . (2025). Impact of aircraft noise on hearing and physiological health using cognitive and physiological evaluations in military personnel. Discover Med. 2, 63. doi: 10.1007/s44337-025-00263-330311153

[B263] Sibbald B. (2001). Military's environmental medicine research pushes limits of human endurance. CMAJ 164 (1), 85–86. PMC80657 11202676

[B264] Sim D. Brothers M. C. Slocik J. M. Islam A. E. Maruyama B. Grigsby C. C. . (2022). Biomarkers and detection platforms for human health and performance monitoring: A review. Adv. Sci. (Weinh) 9, e2104426. doi: 10.1002/advs.202104426 35023321 PMC8895156

[B265] Sloan R. P. Shapiro P. A. Lauriola V. McIntyre K. Pavlicova M. Choi C. J. . (2021). The impact of aerobic training on cardiovascular reactivity to and recovery from psychological and orthostatic challenge. Psychosom Med. 83, 125–137. doi: 10.1097/psy.0000000000000896 33337592 PMC7858227

[B266] Smith E. Burrell C. (2017). A Study of Risk Factors and Prevalence for Noise-Induced Hearing Loss in Canadian Armed Forces Pilots (Ottawa, Canada: Defence Research and Development Canada).

[B267] Soares A. B. F. Almeida M. F. Franchini E. Ferreira F. G. Campos F. A. D. (2025). Heart rate variability in military pilots during flight: a scoping review. Military Med. 190, e515–e522. doi: 10.1093/milmed/usae390 39141423

[B268] Sobotnicka E. Mocha J. Badura G. Sobotnicki A. Dziuda Ł. (2025). The concept of an innovative system for monitoring physiological parameters under extreme conditions. Electronics 14, 889. doi: 10.3390/electronics1405088930654563

[B269] Solano-Atehortua J. M. Castrillon G. Suarez-Revelo J. X. Sanchez-Lopez J. D. Vargas-Tejada D. A. Valentina H. C. . (2026). Linking allostatic load, heart rate variability and brain functional networks and structures in healthy men. Psychoneuroendocrinology 186, 107759. doi: 10.1016/j.psyneuen.2026.107759 41564709

[B270] Song L. Liu M. Li S. Piao Y. (2025). Effects of aircraft noise on pilots' auditory perception. Noise Health 27, 819–826. doi: 10.4103/nah.nah_151_25 41482911 PMC12818493

[B271] Spinelli J. C. Suleski B. J. Wright D. E. Grow J. L. Fagans G. R. Buckley M. J. . (2025). Wearable microfluidic biosensors with haptic feedback for continuous monitoring of hydration biomarkers in workers. NPJ Digital Med. 8, 76. doi: 10.1038/s41746-025-01466-9 39893305 PMC11787291

[B272] Stefanska O. Barbarska O. Minkiewicz-Zochniak A. (2025). Health implications of shift work in airline pilots and cabin crew: A narrative review and pilot study findings. Nutrients 17, 3906. 10.3390/nu17243906 PMC12735491 41470851

[B273] Stensrud R. Valaker S. Mikkelsen B. Betten S. (2020). 5th generation fighter aircraft and future command and control: A note on some possible changes. 25th ICCRTS, 1–29.

[B274] Stott J. R. (2013). Orientation and disorientation in aviation. Extrem Physiol. Med. 2, 2. doi: 10.1186/2046-7648-2-2 23849216 PMC3710190

[B275] Stott J. R. Benson A. J. (2016). “ Spatial orientation and disorientation in flight,” in Ernsting's Aviation and Space Medicine 5th Ed (Boca Raton, FL: CRC Press), 281–320.

[B276] Strong R. Samu J. Pan B. D. Liu D. (2026). Pilot acceptance of wearable fatigue monitoring technology using extended technology acceptance model. J. Air Transportation 34, 23–37. doi: 10.2514/1.d0462

[B277] Summerfield D. Raslau D. Johnson B. Steinkraus L. (2018). “ Physiologic challenges to pilots of modern high performance aircraft,” in Aircraft Technology. Ed. Melih Cemal K. ( IntechOpen, Rijeka). Ch. 3.

[B278] Svensson E. Thanderz M. A. Sjoberg L. Gillberg M. (1988). Military flight experience and sympatho-adrenal activity. Aviat. Space Environ. Med. 59, 411–416. 3390097

[B279] Svoboda A. Boril J. Bauer M. Costa P. Blasch E. (2019). Information Overload in Tactical Aircraft (Piscataway, NJ: IEEE).

[B280] Taha D. H. Abd Alwahab D. Obiad R. M. (2025). Physiological changes in the autonomic nervous system under chronic stress: A systematic review. J. Sci. Volume 3, 56–61.

[B281] Taheri Gorji H. Wilson N. VanBree J. Hoffmann B. Petros T. Tavakolian K. (2023). Using machine learning methods and EEG to discriminate aircraft pilot cognitive workload during flight. Sci. Rep. 13, 2507. doi: 10.1038/s41598-023-29647-0 36782004 PMC9925430

[B282] Tait J. L. Aisbett B. Corrigan S. L. Drain J. R. Main L. C. (2024). Recovery of cognitive performance following multi-stressor military training. Hum. Factors 66, 389–403. doi: 10.1177/00187208221086686 35549578

[B283] Tam C. K. (2025). New noise component of supersonic jets: Laboratory jets to high-performance aircraft. AIAA J. 63, 3752–3763. doi: 10.2514/1.j064769

[B284] Tasiou A. Tzerefos C. Janssen I. K. Karagianni M. D. Peramatzis K. Tsianaka E. . (2026). Flight stressors: Pathophysiological principles guiding safe fixed-wing aeromedical transport of critically ill patients. Cureus 18, e102770. doi: 10.7759/cureus.102770 41783869 PMC12954819

[B285] Thanh X. N. Hong P. N. Ngoc T. T. Minh P. N. Quoc T. P. Duy T. N. . (2026). Heart rate, blood pressure, and SpO(2) responses to simulated 5000 m hypobaric exposure in healthy male Vietnamese pilots. Physiol. Rep. 14, e70733. doi: 10.14814/phy2.70733 41673795 PMC12894062

[B286] Thayer J. F. Hansen A. L. Saus-Rose E. Johnsen B. H. (2009). Heart rate variability, prefrontal neural function, and cognitive performance: the neurovisceral integration perspective on self-regulation, adaptation, and health. Ann. Behav. Med. 37, 141–153. doi: 10.1007/s12160-009-9101-z 19424767

[B287] Thomas R. L. Albelo J. L. Wiggins M. (2023). Enhancing pilot training through virtual reality: Recognizing and mitigating aviation visual and vestibular illusions. Int. J. Aviation Aeronautics Aerospace 10, 10. doi: 10.58940/2374-6793.1839

[B288] Tornero-Aguilera J. Clemente-Suárez V. Villafaina S. Moyano Galán M. Fuentes-García J. (2025). Physiological demands of real and simulated combat flight. Romanian J. Military Med. 128, 248–255. doi: 10.55453/rjmm.2025.128.3.9

[B289] Tornero-Aguilera J. F. Fernandez-Elias V. E. Clemente-Suarez V. J. (2022). Ready for combat, psychophysiological modifications in a close-quarter combat intervention after an experimental operative high-intensity interval training. J. Strength Cond Res. 36, 732–737. doi: 10.1519/jsc.0000000000003495 32187148

[B290] Tripp L. D. Warm J. S. Matthews G. Chiu P. Y. Bracken R. B. (2009). On tracking the course of cerebral oxygen saturation and pilot performance during gravity-induced loss of consciousness. Hum. Factors 51, 775–784. doi: 10.1177/0018720809359631 20415154

[B291] Tripp L. D. Warm J. S. Matthews G. Chiu P. Werchan P. Deaton J. E. (2006). +Gz acceleration loss of consciousness: time course of performance deficits with repeated experience. Hum. Factors 48, 109–120. doi: 10.1518/001872006776412144 16696261

[B292] Tutton W. (2018). High-G flight: physiological effects and countermeasures [Book review. Ergonomics 61, 473. doi: 10.1080/00140139.2017.136354737339054

[B293] Uhuegho K. A N. A. I.I A. Fadele A. A. (2026). A systematic review of the literature on the relationship of pilot flight experience, stress, automation dependency, and complacency. Adv. Multidiscip. Sci. Res. J. Publ. 14, 71–80. doi: 10.22624/aims/digital/v14n1p5x42647824

[B294] Ulvr J. Rackova P. (2025). “ New challenges in the process of future military pilots selection,” in Changes and Innovations in Social Systems (Cham: Springer), 255–263.

[B295] U.S. Marine Corps (2015). “ U.S. Marines Corps declares the F-35B operational,” in Marines.Mil (Washington, DC: United States Marine Corps).

[B296] Van Cutsem J. van den Berg N. H. Flores B. Aidara Y. Martin A. Musat A. . (2026). Circulating biomarkers of military-specific performance resilience: A systematic review. Curr. Psychiatry Rep. 28, 14. doi: 10.1007/s11920-025-01652-2 41714467 PMC12920363

[B297] Van der Burg E. Ledegang W. D. Kooi F. L. Houben M. M. J. Groen E. L. (2025). Attentional tunneling in pilots during a visual tracking task with a head mounted display. Hum. Factors 67, 63–78. doi: 10.1177/00187208241236395 38445657

[B298] van Gennip A. C. E. Satizabal C. L. Tracy R. P. Sigurdsson S. Gudnason V. Launer L. J. . (2024). Associations of plasma NfL, GFAP, and t-tau with cerebral small vessel disease and incident dementia: longitudinal data of the AGES-Reykjavik Study. Geroscience 46, 505–516. doi: 10.1007/s11357-023-00888-1 37530894 PMC10828267

[B299] Varis N. Leinonen A. Parkkola K. Leino T. K. (2022). Hyperventilation and hypoxia hangover during normobaric hypoxia training in Hawk simulator. Front. Physiol. 13, 942249. doi: 10.3389/fphys.2022.942249 35910556 PMC9326353

[B300] Varis N. Leinonen A. Perala J. Leino T. K. Husa L. Sovelius R. (2023). Delayed drowsiness after normobaric hypoxia training in an F/A-18 Hornet simulator. Aerosp. Med. Hum. Perform. 94, 715–718. doi: 10.3357/amhp.6238.2023 37587630

[B301] Vartanian O. Boscarino C. Jarmasz J. Zotov V. (2022). “ Training-related stress and performance in the military,” in Handbook of Military Sciences. Ed. Sookermany A. M. ( Springer International Publishing, Cham), 1–21.

[B302] Vartanian O. Bouak F. Lam Q. Miles R. (2025). Initial psychometric validation of a self-report measure of the symptoms of mild hypoxic hypoxia. Aerosp. Med. Hum. Perform. 96, 851–856. doi: 10.3357/amhp.6626.2025 40925617

[B303] Veksler B. Z. Morris M. B. Swan G. Stephenson A. Tuttle J. (2025). Biomathematical modeling of fatigue and cognitive performance. Proc. Hum. Factors Ergon. Soc Annu. Meet. 69, 563–567. doi: 10.1177/10711813251372096

[B304] Venus M. (2022). Interactions of international pilots' stress, fatigue, symptoms of depression, anxiety, common mental disorders and wellbeing. Int. J. Aviation Aeronautics Aerospace 9, 4. doi: 10.15394/ijaaa.2022.1667

[B305] Verissimo Franca de Oliveira R. Barros de Sa G. Dos Santos Pinheiro A. C. Claudia Jacome da Camara A. Diogenes P. Calderaro Calvo A. P. . (2025). Acute metabolic effects in Brazilian A-29 fighter pilots by NMR-based metabolomics. J. Proteome Res. 24, 4033–4043. doi: 10.1021/acs.jproteome.5c00129 40689497 PMC12322951

[B306] Villafaina S. Fuentes-Garcia D. J. P. Gusi N. Tornero-Aguilera J. F. Clemente-Suarez V. J. (2021). Psychophysiological response of military pilots in different combat flight maneuvers in a flight simulator. Physiol. Behav. 238, 113483. doi: 10.1016/j.physbeh.2021.113483 34097973

[B307] Wang H. Jiang N. Pan T. Si H. Li Y. Zou W. (2020). Cognitive load identification of pilots based on physiological‐psychological characteristics in complex environments. J. Adv. Transp. 2020, 5640784. doi: 10.1155/2020/5640784

[B308] Wang L. Yang C. Yan D. Ye L. Chen X. Ma S. (2025b). The effects of flight training on flying cadets' brain structure. PloS One 20, e0313148. doi: 10.1371/journal.pone.0313148 39928587 PMC11809809

[B309] Wang H. Zhao Y.-Y. Wu T.-Y. Wang X. Zhao C.-F. Lu C. . (2025a). Visual fatigue factors associated with night-vision training among military pilots. Aerosp. Med. Hum. Perform. 96, 1050–1055. doi: 10.3357/amhp.6307.2025 41360145

[B310] Wehrwein E. A. Orer H. S. Barman S. M. (2016). Overview of the anatomy, physiology, and pharmacology of the autonomic nervous system. Compr. Physiol. 6, 1239–1278. doi: 10.1002/j.2040-4603.2016.tb00714.x 27347892

[B311] Weigand M. Embacher S. (2024). “ The next generation cockpit: Requirements of fighter pilots in a highly automated environment”, in: Proceedings of the 2nd International Conference on Cognitive Aircraft Systems (ICCAS 2024) (Setubal: SciTePress), 43–50.

[B312] Whinnery J. Murray D. (1990). Enhancing tolerance to acceleration (+ Gz) stress. “HB” Maneuver Final Report Naval Air Dev. Center Warminsterpa Air Vehicle Crew Syst. Technol. Department.

[B313] Wilson G. F. (1992). Applied use of cardiac and respiration measures: practical considerations and precautions. Biol. Psychol. 34, 163–178. doi: 10.1016/0301-0511(92)90014-l 1467392

[B314] Wittels H. L. Wishon M. J. Davilla H. McDonald S. M. Temme L. A. Wittels S. H. (2026). Autonomic nervous system responses to aviation live-fire combat training: An effective tool for operator state monitoring of United States warfighter aircrew. Mil. Med. 191, e641–e647. doi: 10.1093/milmed/usaf295 40511733

[B315] Wittels H. L. Wittels S. H. Wishon M. J. Vogl J. St Onge P. McDonald S. M. . (2024). Examining the influence of cognitive load and environmental conditions on autonomic nervous system response in military aircrew: A hypoxia-normoxia study. Biol. (Basel) 13, 343. doi: 10.3390/biology13050343 38785825 PMC11118911

[B316] Woltjer R. Ramberg R. Artman H. Aronsson S. Mitchell M. Oskarsson P.-A. (2024). The future of fighter pilot training? Live virtual constructive in large force exercises: perceived and expected training value. Int. J. Aerosp. Psychol. 34, 20–41. doi: 10.1080/24721840.2023.224744437339054

[B317] Wu L. Chen Y. Liu X. Fang P. Feng T. Sun K. . (2022). The influence of job burnout on the attention ability of army soldiers and officers: Evidence from ERP. Front. Neurosci. 16, 992537. doi: 10.3389/fnins.2022.992537 36419460 PMC9676458

[B318] Wu Z. Qiao Z. Chen S. Fan S. Liu Y. Qi J. . (2024). Interstitial fluid-based wearable biosensors for minimally invasive healthcare and biomedical applications. Commun. Mater. 5, 33. doi: 10.1038/s43246-024-00468-637880705

[B319] Yang W. Li Y. Liu L. Si H. Wang H. Pan T. . (2026a). Research on pilot workload identification based on EEG time domain and frequency domain. Aerospace 13, 114. doi: 10.3390/aerospace1302011430654563

[B320] Yang Z. Li C. Yao Y. (2026b). Research progress on surface electromyography in monitoring G-induced loss of consciousness. J. Clin. Med. Res. 6, 645. doi: 10.32629/jcmr.v6i4.4809

[B321] Yıldız E. (2024). Advancing aviation through human-computer interaction: A focus on safety, efficiency, and performance. J. Aerospace Sci. Manage. 2, 81–95.

[B322] Zhang H. Wang C. Wang P. (2026). An intelligent evaluation algorithm for pilot flight training ability based on multimodal information fusion. Sensors (Basel) 26, 2245. doi: 10.3390/s26072245 41978030 PMC13075279

[B323] Zhang Y. Zhang Y. Peng F. Liao Y. Deng X. Song H. . (2020). “ Study on autonomic nervous stability training of military pilots”, in: International Conference on Man-Machine-Environment System Engineering (Singapore: Springer Nature Singapore).

[B324] Zhao Y. Gao D. Liu Y. B. Xue J. J. Lu X. Dong J. J. . (2024). Spectra of intracranial diseases in Chinese military pilots (cadets) unqualified for transfer to pilot modified high performance aircraft. World J. Radiol. 16, 638–643. doi: 10.4329/wjr.v16.i11.638 39635313 PMC11612804

[B325] Zhao Y. Zhu K. Guo W. Xu H. Zhang J. Zou J. . (2025). Differentiating pilot distress and eustress via multimodal physiology: towards enhanced human-system integration in intelligent cockpits. Ergonomics, 1–23. doi: 10.1080/00140139.2025.2553131 40878643

[B326] Zhou W. G. Yu P. P. Wu L. H. Cao Y. F. Zhou Y. Yuan J. J. (2024). Pilot turning behavior cognitive load analysis in simulated flight. Front. Neurosci. 18, 1450416. doi: 10.3389/fnins.2024.1450416 39376543 PMC11456565

